# Persistence of surrogates for high consequence viral and bacterial pathogens in a pilot-scale activated sludge treatment system

**DOI:** 10.1371/journal.pone.0275482

**Published:** 2022-10-07

**Authors:** Donald A. Schupp, Adam C. Burdsall, Rendahandi G. Silva, John Lee Heckman, E. Radha Krishnan, Jeffrey G. Szabo, Matthew Magnuson

**Affiliations:** 1 APTIM Federal Services, Cincinnati, Ohio, United States of America; 2 Homeland Security and Materials Management Division, Center for Environmental Solutions and Emergency Response, Office of Research and Development, U.S. Environmental Protection Agency, Cincinnati, Ohio, United States of America; Fuzhou University, CHINA

## Abstract

The persistence of high consequence public health pathogens in a wastewater treatment system can significantly impact worker safety, as well as the public and downstream water bodies, particularly if the system is forced to shut down the treatment processes. This study utilizes organism viability to compare the persistence of three pathogen surrogates in wastewater using a pilot-scale activated sludge treatment (AST) system, operated to mimic treatment processes of large-scale plants. *Bacillus globigii* spores, surrogate for *Bacillus anthracis*, persisted in the AST system for at least a 50-day observation period leading to a possible steady condition far beyond the solid retention time for sludge particles. MS2 bacteriophage, surrogate for Poliovirus and other non-enveloped enteric viruses, was observed for up to 35 days after introduction, which largely and expectedly correlated to the measured solid retention time. Phi-6 bacteriophage, a surrogate for Ebola virus and other enveloped viruses, was detected for no more than 4 days after introduction, even though the AST system was operated to provide three times slower solids removal than for the other surrogates. This suggests Phi-6 is subject to inactivation under AST conditions rather than physical removal. These results may suggest similar persistence for the surrogated pathogens, leading to appropriate consequence management actions.

## 1. Introduction

Wastewater treatment facilities (i.e., water resource recovery facilities [WRRFs]) are engineered to protect human health and the environment from a range of pathogenic micro-organisms, and treatment practices are usually based on micro-organisms that normally occur in the wastewaters received by the facility. However, novel pathogens with potentially high consequences emerge for which the protection afforded by WRRFs is unknown. Despite engineering controls, wastewater plant workers and the public can be exposed to untreated wastewater from both ingestion and airborne exposure [1].

The anthrax terror attacks of 2001 brought particular attention to disposal methods of impacted decontamination wash waters. Federal guidance was developed but not finalized [2] and interest in the topic waned. Then, Ebola outbreaks in 2014 piqued international interest in providing the wastewater industry with vital information to support ongoing wastewater treatment services and to prevent pathogen spread to wastewater workers, the public, and the environment. Guidance was developed again [2], but the process took time, a luxury WWRFs may not necessarily have. Pathogens can quickly enter the wastewater system in numerous ways, sometimes without operators’ knowledge, given the limits of some of the standard detection methods [3]. Entry can result from hospital and household wastes, precipitation events, and aqueous wastes from decontamination activities.

This strengthened the resolve of the wastewater industry and associated stakeholders to be prepared for the next high consequence pathogen—which could come in unanticipated forms (e.g., SARS-CoV-2). The risks posed by the passage of SARS-CoV-2 and other viruses through the wastewater treatment plants are still unclear [4, 5]. The result was a workshop representing a partnership between the Water Environment Research Foundation (now known as the Water Research Foundation), the US National Science Foundation, and the US Environmental Protection Agency (EPA). In the workshop report [6], experts from these groups and WWRF stakeholders identified several topics, including pathogen persistence in Activated Sludge Treatment (AST) systems, as prominent gaps in our knowledge of high consequence pathogens in wastewater collection and WWRFs. The AST system is a vital unit process in WRRFs. If the AST system is not properly managed, pathogens may spread via aerosolization and discharge to receiving waters if secondary effluent disinfection is ineffective, or if the potentially overwhelming mass of waste sludge that is produced daily is not disposed of properly.

*Bacillus* (*B*.) *globigii* spores were utilized as a surrogate for *Bacillus* (*B*.) *anthracis* and other sporulating bacteria. MS2 bacteriophage (hereafter “MS2”) is a surrogate for non-enveloped enteric viruses (e.g., poliovirus). Phi-6 bacteriophage (hereafter “Phi-6”) is a surrogate for enveloped viruses like Ebola. These three surrogates cover three of the major classes of high consequence pathogens that might persist in the treatment pathways of a WRRF either due to mismanagement or insufficient secondary treatment.

The purpose of this paper is to infer the persistence of novel, high consequence pathogens in the AST system. Because full or pilot scale systems often have inadequate biosafety measures for conducting research directly with hazardous pathogens, using similar representative organisms as “surrogates” for testing the fate, transport, and persistence of high consequence pathogens in various environments is common [7–10]. In this study, the fate of a broad group of surrogate pathogens entering an AST system was evaluated through pilot-scale studies that enabled the persistence of the viable pathogen surrogates in different parts of the AST system to be studied over the course of days or weeks.

## 2. Materials and methods

### 2.1 Pilot-scale AST system operation

**Fig 1:**
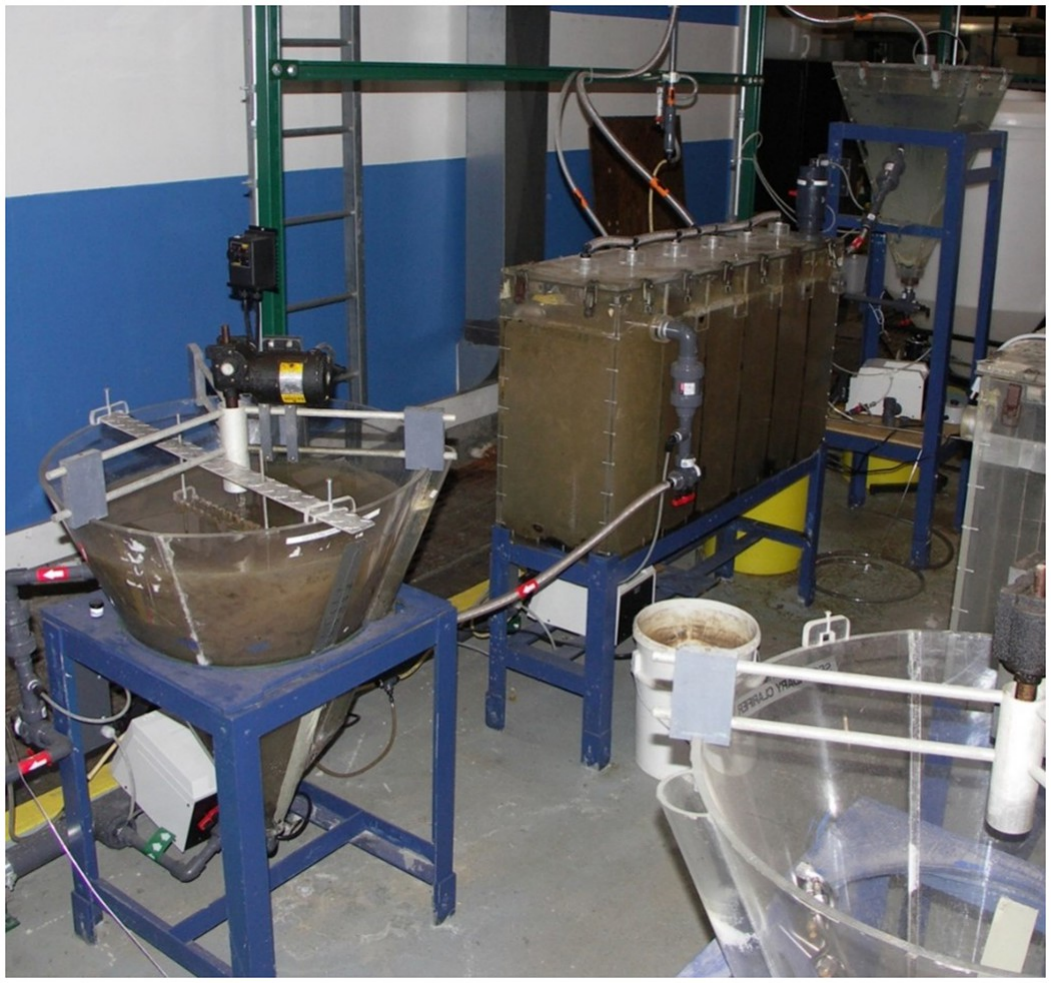
Photograph of the pilot-scale AST system. Right/rear: Primary clarifier; middle: aeration basin; left/front: secondary clarifier.

**Fig 2:**
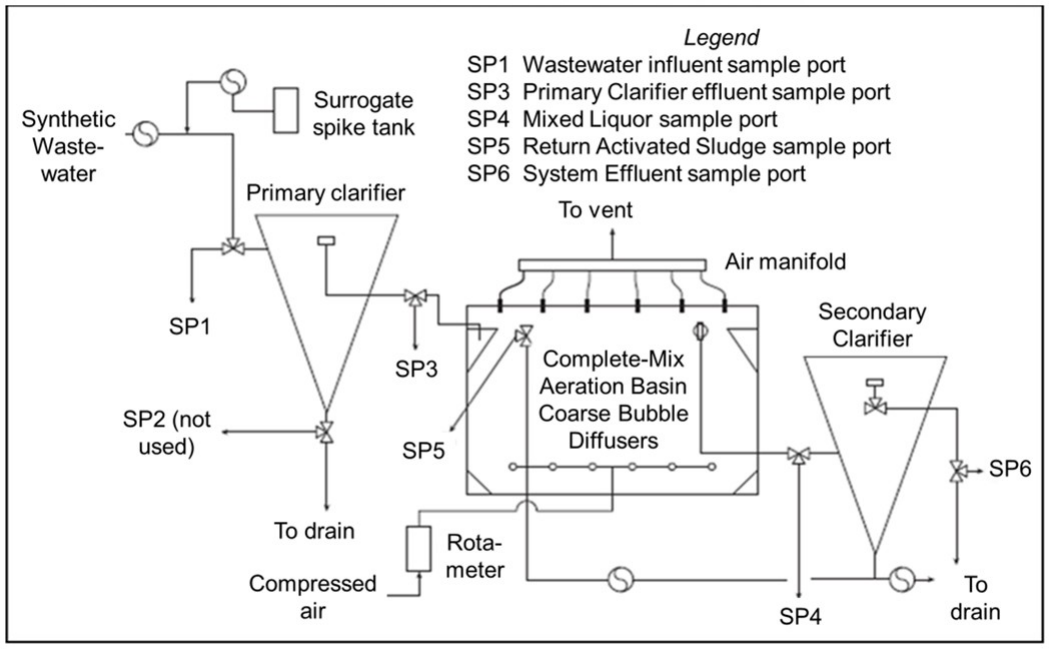
Schematic diagram of the pilot-scale AST system. Note that sample port 2 (SP2) is not used for this study.

Experiments were performed in a pilot-scale conventional AST system consisting of a poly (methyl methacrylate) 25 L primary clarifier, 213 L aeration basin, and 52 L secondary clarifier (Fig 1) located at the US EPA’s Test and Evaluation (T&E) Facility in Cincinnati, Ohio. The AST system was seeded using activated sludge collected from the adjacent Metropolitan Sewer District of Greater Cincinnati (MSDGC), Mill Creek Wastewater plant. Fig 2 is a schematic of the AST system, labeled with various sampling ports from which samples were withdrawn to study the transport of the surrogates through the system.

Before each experiment, the AST was disassembled and thoroughly sanitized, reassembled, reseeded using sludge from the MSDGC plant, and allowed to stabilize. A more thorough description of this is available in Section 2 of S1 Text. Briefly, the system was allowed to stabilize, after which grab samples were collected from the AST system prior to surrogate injection to verify a starting concentration of zero and specified nutrient removal status. Five sample ports were used to collect grab samples in the pilot-scale AST system: SP1-system influent; SP3-primary clarifier effluent/aeration tank influent; SP4-aeration tank effluent; SP5-return activated sludge (RAS); and SP6-system effluent (Fig 2).

Six experiments were run in this study with two experiments for each surrogate. Most of the experiments had samples collected at t = 0, 0.67 (~4 hours), 1, 7, 14, 21, 28, 35, 42, and 48 days. The first *B*. *globigii* experiment had more samples collected. Experimental conditions for the surrogate stock suspensions are summarized in Table 1. As evidenced by the *B*. *globigii* experiments, the AST system was stable and repeatable, so the second MS2 experiment was conducted using synthetic wastewater instead of dechlorinated tap water to examine the effects of ionic strength and organic matter composition. The synthetic wastewater composition is found in the A Table of the S1 Text. Also note that Phi-6 concentration was lower (Table 1) due to a difficulty achieving similar concentrations to that of MS2. To compensate for this lower concentration, injection period and injection rate were changed to increase detection.

**Table 1:** Experimental conditions for all experiments.

	*B*. *globigii*		MS2		Phi-6	
Experiment:	1	2	1	2	1	2
**Suspension composition**	Dechlorinated tap water		Dechlorinated tap water	Synthetic wastewater	Dechlorinated tap water	
**Surrogate injection period**	72 hours		72 hours		24 hours	
**Influent flow rate**	0.5 L/min		0.5 L/min		0.25 L/min	
**Target Surrogate Concentration**	10^7^ CFU/100 mL		10^7^ PFU/100 mL		10^6^ PFU/100 mL	

Grab samples were taken to monitor the AST’s stability and conditions for standard water quality parameters such as chemical oxygen demand (COD) [11], ammonia [12], and total suspended solids (TSS) [13]. Temperature and dissolved oxygen (DO) levels were monitored in the system aeration tank when grab samples were taken. Temperature and DO were monitored using a YSI Sonde 600 series. Air flow to the aeration tank was maintained at approximately 25 L/min to ensure DO was greater than 2.0 mg/L.

To apply the results of this study more broadly to AST systems, this paper relates the findings with the surrogates to two fundamental properties of an AST system that can govern a solid or liquid contaminant’s persistence are the hydraulic retention time (HRT) and the solid retention time (SRT)/cell retention time. Hydraulic retention time is defined as the average length of time a particle of liquid stays within a reactor of constant volume when a constant flow rate and liquid density are maintained [14]. Solid retention time is the length of time a particulate tends to stay in a bioreactor and can be defined as the mass of a particulate constituent divided by the mass discharged over time [14].

HRT was calculated using the following equation: HRT = reactor and settling tank volume / influent flow rate [14, 15]. SRT was calculated according to Eq 1 for SRT described in Grady et al. [14]. These values are averaged over the course of each experiment with error of one standard deviation.

$$SRT=\;\left(\frac{V\times MLSS}{W\times RAS}\right)$$ (1)

where V = volume of aeration tank, W = waste rate (L/min), MLSS = TSS (in mg/L) from the aeration tank outlet (a.k.a. Mixed Liquor Suspended Solids), determined according to an SOP based on American Public Health Association (1998) [13]. RAS (Return Activated Sludge) = concentration of TSS (in mg/L) out of the secondary clarifier and recycled to the aeration chamber. These measured values and the resulting SRT calculations can be found in the B Table in S1 Text.

### 2.2 Surrogate pathogens and viability detection

*B*. *globigii* cultivation and sampling was completed according to methods previously reported in Szabo et al. [16]. A *B*. *globigii* stock suspension like the stock that Szabo et al. [16] used was grown in generic sporulation medium [16, 17] for 5 days at 35°C in a shaking (145 rpm) incubator. The enumeration of spores is described in the references above according to the methods described by the American Public Health Association [18] (1999). In brief, a sub-sample of the spore-containing broth was heat-shocked for 10 minutes at 80°C to kill vegetative cells and analyzed to determine the spore concentration. Surviving bacterial spores in the sample were filter-captured using a 47 mm diameter 0.45-μm pore mixed ester cellulose membrane filter and analyzed by culturing on tryptic soy agar (TSA), permitting the spores to germinate and produce vegetative cells. Samples obtained from each sampling point of the AST system were pasteurized to kill vegetative cells and contaminant cells, leaving only spores, and serially diluted. Diluted samples were analyzed in duplicate using membrane filtration as described above after overnight incubation on TSA at 35°C. Concentrations were back calculated in colony forming units (CFU)/100 mL. The procedure also included both positive and negative controls to ensure that contamination would not skew results.

*Escherichia coli* bacteriophage MS2 (ATCC^®^ 15597-B1^™^), host *E*. *coli* (ATCC^®^ 15597^™^) and *Pseudomonas syringae* bacteriophage Phi 6 (ATCC^®^ 21781-B1^™^) were obtained from American Type Culture Collection, ATCC (Manassas, VA). Leonard Mindich of the Public Health Research Institute (Newark, NJ) provided the host *P*. *syringae* (LM2489). EPA Method 1602 [19] (USEPA, 2001) was used to cultivate the bacteriophage and analyze MS2 samples. In brief, samples (100 mL) were mixed with 10 mL *E*. *coli* host from a log-phase culture and 0.5 mL 1 M MgCl_2_ without antibiotics. This sample/host mixture was kept at 36°C for 5 min in a water bath prior to being maintained at 48°C for 4 min in another water bath. The sample/host mixture was combined with 100 mL molten, double strength TSA without antibiotics, also kept at 48°C in a water bath. Twenty mL of the final mixture was pipetted into each of ten 10 cm diameter Petri plates. Plaques on each TSA plate were counted after overnight incubation at 37°C. A method blank is used in this method to test for contamination. Bacteriophage samples, unlike spores, cannot be pasteurized to eliminate all contamination and no antibiotics were used. Sampling relied upon dilution to produce quantifiable results, but too little dilution resulted in prohibitive amounts of contamination.

Phi-6 was analyzed using an adaptation of EPA method 1602 [19]. The cultivation methods of MS2 and Phi-6 were similar with a few minor adjustments. The method used bacterial host *P*. *syringae* (LM 2489). Also, in the procedure, 100 mL samples were combined with 20 mL of an overnight culture of host and 0.1 mL ampicillin sodium solution (20 g/L). The sample/host mixture was combined with 100 mL of molten, double-strength Tryptone agar, and 20 mL was pipetted into each of ten 10 cm diameter Petri plates. As with the MS2 samples, samples could not be pasteurized. Plaques on each Tryptone agar plate were counted after overnight incubation at 28°C. Results for both bacteriophages were expressed in plaque forming units (PFU)/100 mL.

In all three surrogate pathogen assays, negative controls were run using sterile phosphate buffer with magnesium. The *B*. *globigii* negative controls were spiked with 1.0 mL *E*. *coli* culture to confirm the success of pasteurization. In both bacteriophage assays, negative controls were prepared using reagent water or sterile dilution buffer and analyzed by single agar layer method for every batch of samples. For the bacteriophages, according to method 1602, a series of ongoing precision and recovery samples are required [19]. One is sampled for each analytical batch, which serves as a positive control [19].

### 2.3 Data analysis

**Fig 3:**
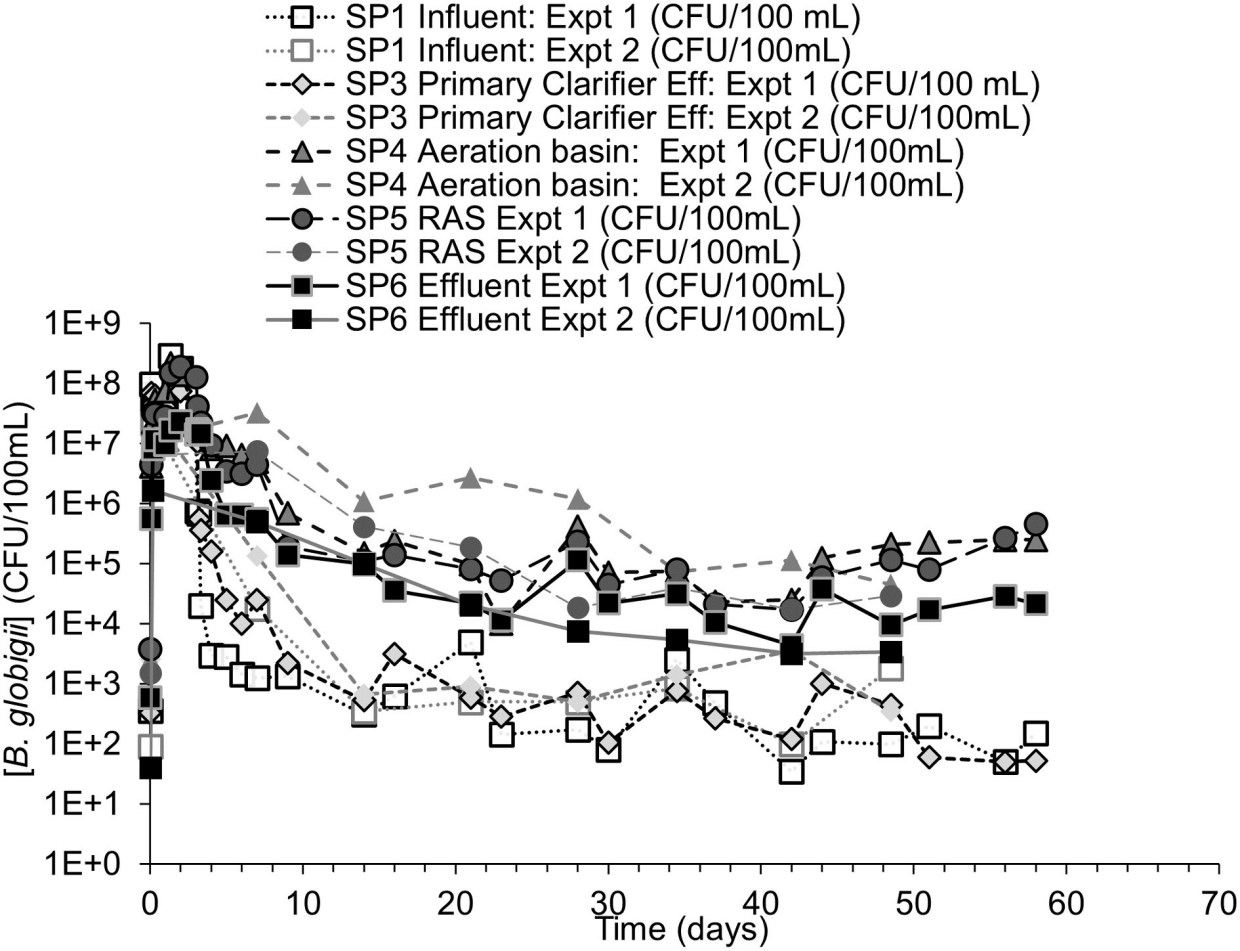
*globigii* concentration response curves for Test 1 and 2. ***B***. Concentration-time plot of individual sample ports over time for experiment 1 and experiment 2.

**Fig 4:**
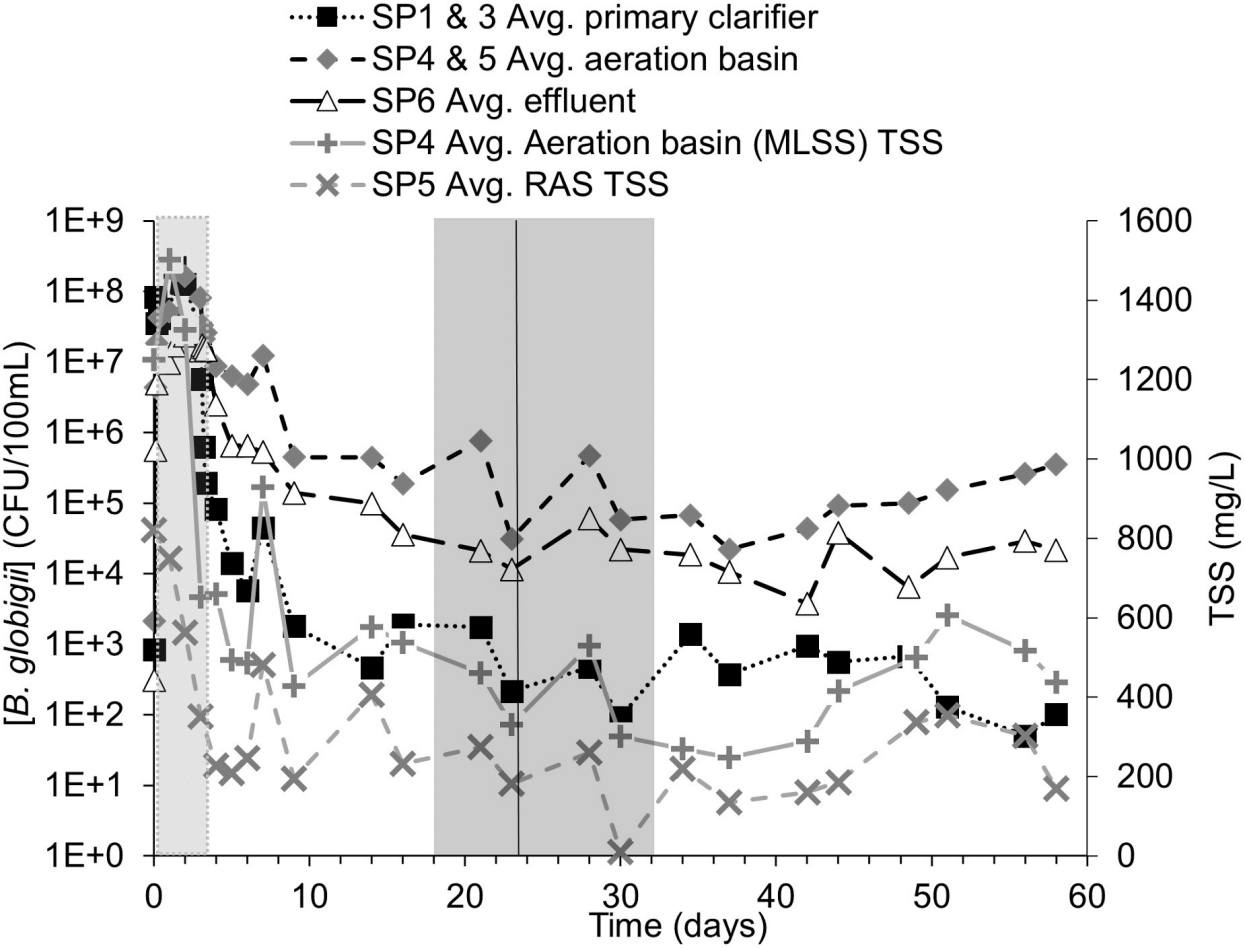
Averaged *B*. *globigii* concentrations and TSS in the RAS and aeration basin effluent. The light gray block shows the possible HRT range of 0.375 to 3.375 days, a black vertical line is the average calculated SRT of 23.6±5.6 days, and the darker gray block represents the possible SRT range of 18–32.1 days. SP1 and SP3 from both experiments were averaged for “average primary clarifier,” and likewise for SP4 and SP5 for “Average aeration basin.” The “+” and “x” series show the TSS in the aeration basin effluent and RAS, respectfully. Error bars were removed for visual clarity. Original data in S2 Text.

**Fig 5:**
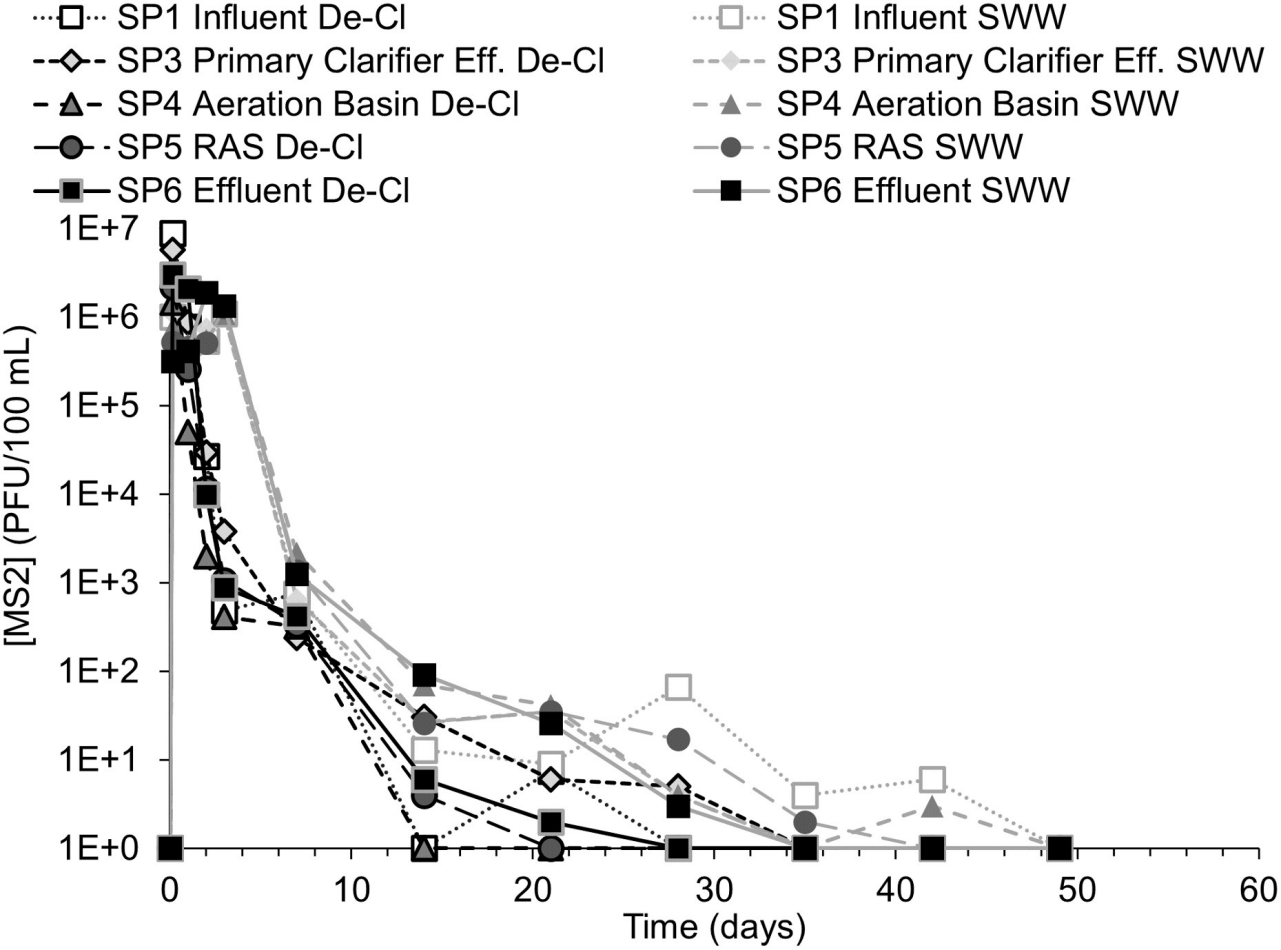
MS2 concentration response curves for Test 1 and 2. Concentration-time plot of individual sample ports over time for experiment 1 and experiment 2.

**Fig 6:**
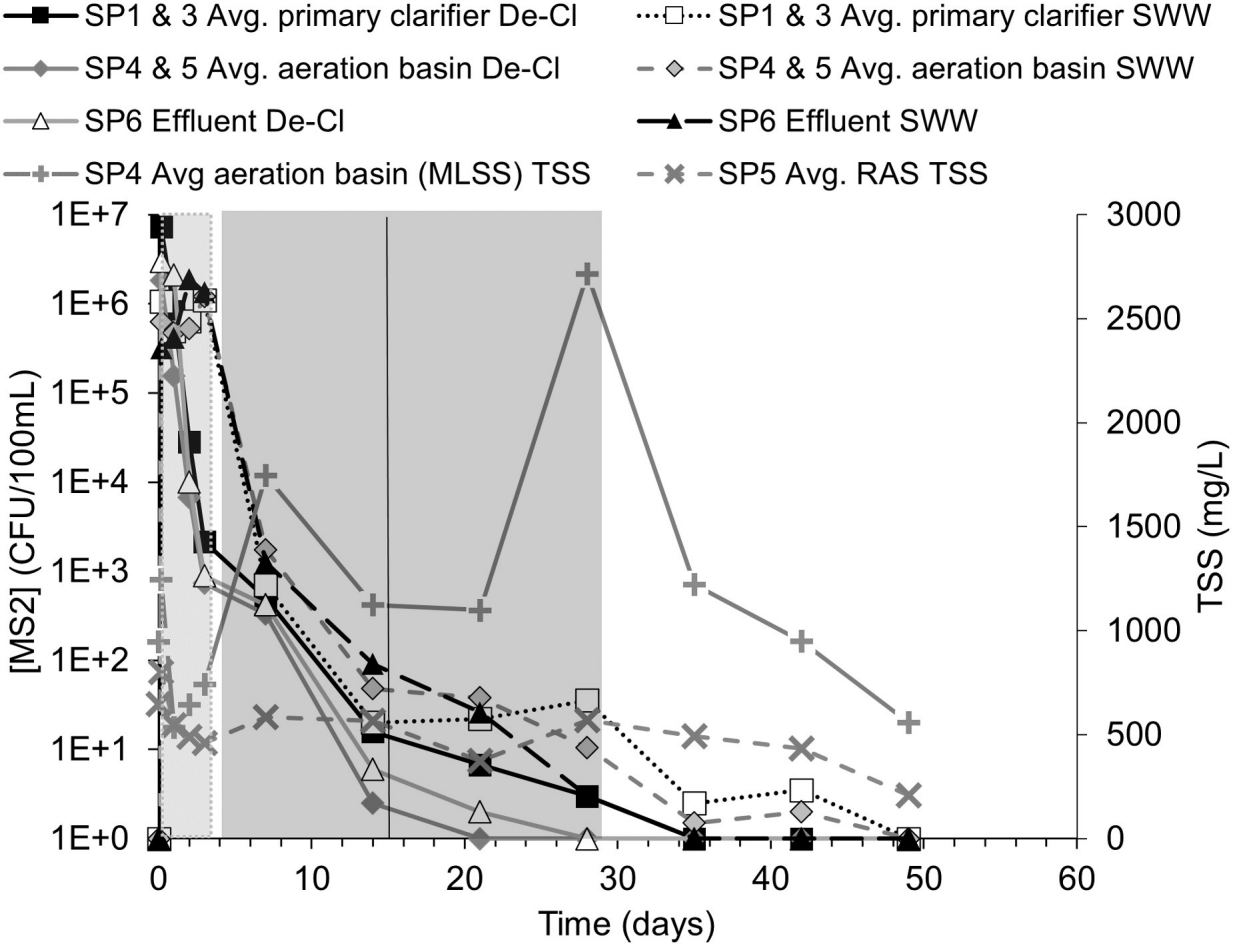
Averaged MS2 concentrations and TSS in the RAS and aeration basin effluent. De-Cl = “dechlorinated water” and SWW = “synthetic wastewater.” The light gray block shows the possible HRT range of 0.375 to 3.375 days. The black vertical line is the average calculated SRT of 15.0±10.9 days and the darker gray bar represents the SRT possible range of 4.1–28.9 days, respectively. SP1 and SP3 were averaged for “average primary clarifier” series. SP4 and SP5 were averaged for “average aeration basin” series. The “+” and “x” series show the TSS in the aeration basin effluent and RAS, respectfully. Error bars were removed for visual clarity. Original data in S2 Text.

**Fig 7:**
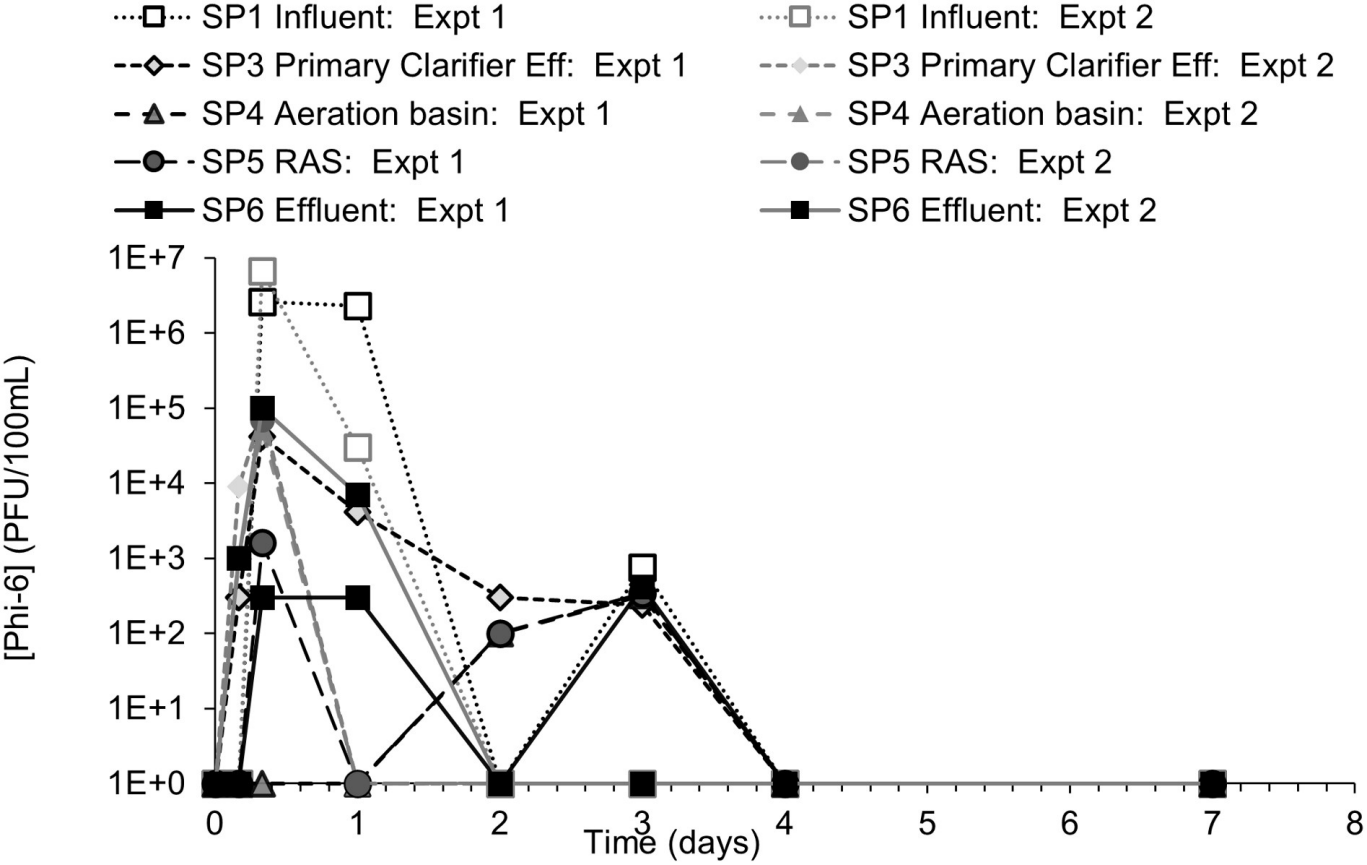
Phi-6 concentration response curves for Tests 1 and 2. Concentration-time plot of individual sample ports over time for experiment 1 and experiment 2.

**Fig 8:**
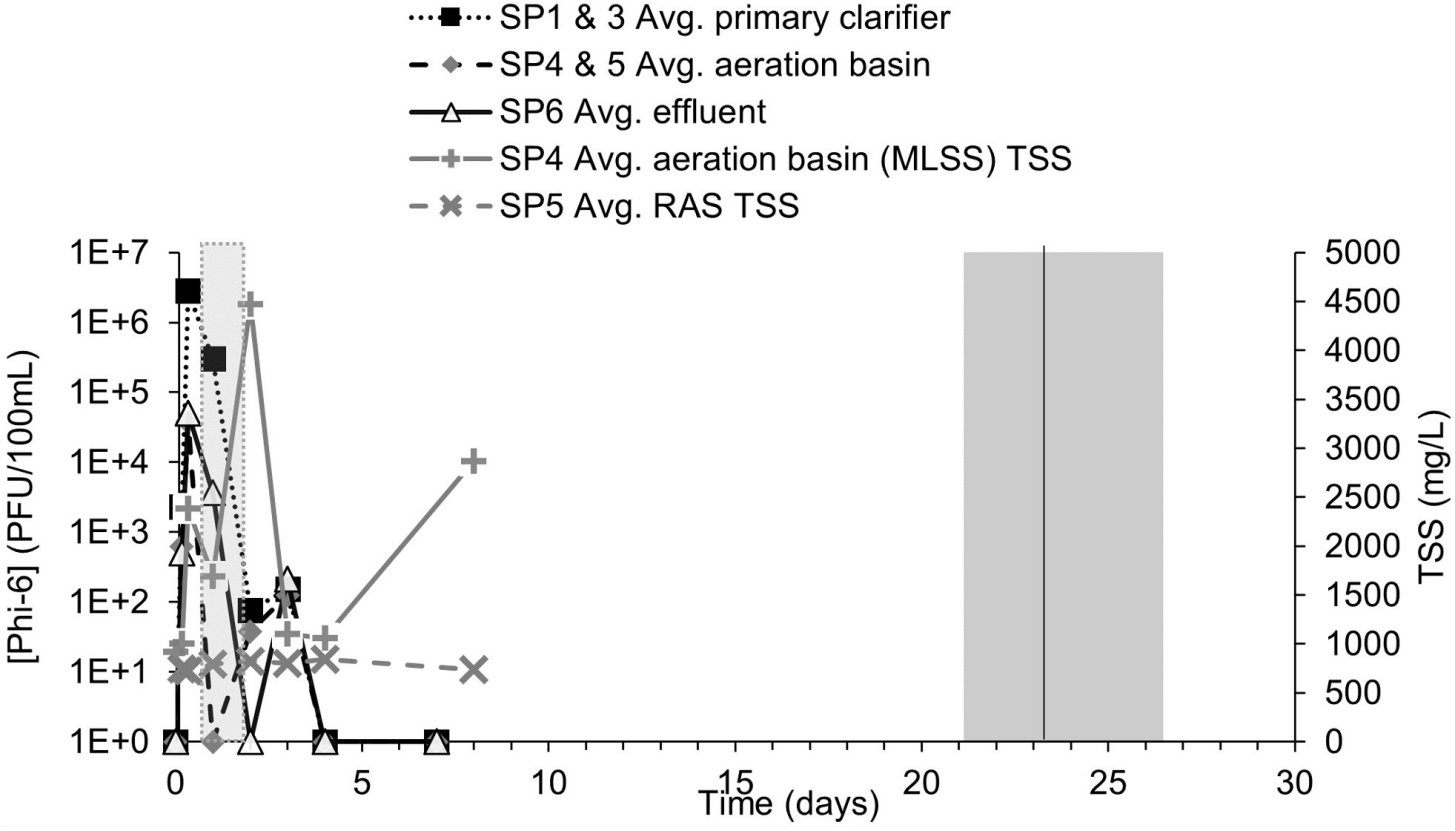
Averaged MS2 concentrations and TSS in the RAS and aeration basin effluent. The light gray block shows the HRT range of 0.75 to 1.75 days. The black vertical line is the average calculated SRT of 23±2 days and the darker gray bar represents the SRT possible range of 21–26.4 days. SP1 and SP3 were averaged for “Average primary clarifier.” SP4 and SP5 were averaged for “Average aeration basin.” The “+” and “x” series show the TSS in the aeration basin effluent and RAS, respectfully. Error bars removed for clarity (Original data in S2 Text).

During each test, surrogate organism counts equal or greater than one were normalized to a volume of 100 mL and presented via log units to better compare the surrogate pathogen behavior. When surrogate counts were non-detect (i.e., zero), the value of one was used in the figures to make log_10_-based plotting possible. The detection limits for *B*. *globigii*, MS2, and Phi-6 were 50 CFU/100mL, 1 PFU/100mL, and 100 PFU/100mL, respectively. These values and their associated time points (i.e., days, weeks) were used in response curves. *B*. *globigii* samples consisted of 3 different dilution levels for samples of duplicate plates. Dilution levels that had too little/no CFU and too many CFU to count were not counted. MS2 and Phi-6 samples consisted of at least 1 level of dilution consisting of 10 plates. Sometimes 2 or 3 dilution levels were used when it was unclear what level of dilution would leave sufficient PFU that can be counted. Data points in Figs 3 to 8 are calculated from averages of those plates.

Due to variability in number of time points among tests and limited number of experimental tests (i.e., duplicates) possible, there were few descriptive statistical tests that could be performed for data analysis, although some 2-variable t-tests (assuming unequal variances) were performed in Excel. Two-variable t-tests were performed to confirm some of the qualitative results using surrogate concentrations data. Data collected after the calculated SRT for *B*. *globigii* and MS2 were assumed to be mostly representative of a steady state condition. These data were compared between individual sample ports to confirm whether the observed differences between sample port concentrations were significant (an α-value of 0.05 was selected, so a p<0.05 was statistically different; Section 5 in S1 Text). Because sample ports 1 and 3 were associated with the primary clarifier, they were averaged together for the “primary clarifier” data. Likewise, sample ports 4 and 5 were averaged for the “aeration basin,” and SP6 was summarized as the “secondary clarifier.” T-tests were conducted to compare the averages of the data associated with the three major parts of the treatment system to help identify what parts of the system showed high persistence of a surrogate. However, because the tests also included so few samples, tests for normality were inconclusive.

Excel’s linear regression function and correlation function were used to analyze the correlation between the concentrations of the surrogates with total suspended solids to examine whether surrogate persistence was dependent on the concentration of suspended solids (Section 6 in S1 Text). The surrogate concentration at a particular location in the system was compared to its corresponding TSS concentration at the same location.

## 3. Results and discussion

### 3.1 AST system performance verification

Overall, the physicochemical water quality parameters among surrogate pathogen tests were within performance expectations. Differences may have arisen due to (i) random experimental variation in the measurement of water quality parameters, (ii) temperature (between 20–27 °C), (iii) airflow changes that were needed to maintain DO target levels, (iv) the complex nature of the sample matrices, (v) physical limitations of the size of the AST system contributing to bulking of the sludge, and (vi) variable inflow and outflow to maintain stable AST system conditions. The AST system removed representative water quality parameters in line with the performance of large-scale wastewater treatment systems [20] (A–C Figs in S1 Text).

The pilot-scale AST system in this study most closely resembled a full-scale modified aeration system, described by Tchobanoglous and Burton (1991) as being like a conventional plug-flow system [15]. These modified aeration plug-flow systems are expected to have MLSS values between 200 and 1000 mg/L and use shorter aeration times and lower BOD like the AST system used in this study [15]. The MLSS in this pilot-scale system dropped as low as 248 mg/L near the end of a *B*. *globigii* experiment but stayed mostly within the range of 300–700 mg/L in *B*. *globigii* and MS2 experiments. MLSS remained between 835 and 875 mg/L in Phi-6 experiments (B Table in S1 Text).

In *B*. *globigii* and MS2 experiments, the HRT was ~9 hours. Since Phi-6 stock concentrations were lower, liquid feed rate was reduced by half, HRT was 18 hours. Note that because of the gradual addition of surrogate, HRT was expressed as a range, beginning at the calculated HRT and extending to the end of the injection period + the calculated HRT. The SRT range was calculated as an average over the course of the experiment within one standard deviation. Thus, the maximum SRT was the end of the injection period + average SRT + standard deviation. SRT in *B*. *globigii* experiments was 18–32.1 days. SRT in MS2 experiments was 4.1–28.9 days. SRT in Phi-6 experiments was 21.2–26.4 days (B Table in S1 Text). Brady [21] indicated that the desirable SRT for conventional wastewater treatment plants was 5 to 15 days. Tchobanoglous and Burton (1991) [15] suggested that times of 3 to 15 days produce a stable, high-quality effluent with good sludge settling characteristics. Full scale systems can have SRTs and TSS concentrations greater and less than those in this pilot scale experiment. The range of SRTs in the current study are near that of several designs for activated sludge processes [15]. In all, the AST system performance is expected to be sufficient to not affect the overall discussion (below) of the surrogate viability/persistence.

### 3.2 Fate of *Bacillus globigii* in AST system

The response curves of measured *B*. *globigii* show the target surrogate pathogen concentration in the system influent (SP1) and aeration tank influent (SP3) during both duplicate tests peaked above 10^8^ CFU/100 mL (Fig 3). An intensive sampling schedule was followed for the first *B*. *globigii* experiment, collecting a higher density of samples, during the first test for: 1) studying the system behavior closely, and 2) economizing on the second *B*. *globigii* test via a less intense sampling schedule. Fig 4 shows details of the persistence of this surrogate pathogen beyond the range of possible SRT. In all of the figures that follow, SP1 = system influent; SP3 = primary clarifier effluent/aeration tank influent; SP4 = aeration tank effluent/MLSS; SP5 = RAS/waste; SP6 = system effluent.

Fig 4 shows averages of the data from the two experiments and groups data by part of the AST system. Fig 4 also shows TSS values in the return activated sludge and aeration basin effluent. There is also a dip in *B*. *globigii* that coincided with a drop in TSS near the beginning of the possible SRT range at ~22 days. *B*. *globigii* persists in the AST at detectable levels and visually appeared to rise beyond the end of the possible SRT range (~32 days) in SP4, 5, and 6 (Fig 4). *B*. *globigii* was not present before the spiking period.

The *B*. *globigii* concentration increased to the target levels in the system influent (SP1) and primary clarifier effluent (SP3) ports within 4 hours and peaked around day 2 (Fig 3). Surrogate concentrations in SP1 decreased to approximately 10^2^−10^3^ CFU/100 mL after day 3 when the injection period ended. *B*. *globigii* concentrations at ports SP1 and SP3 were nearly negligible after day 10 (Figs 3 and 4), showing that the organisms mostly vacated the primary clarifier before the SRT range. Concentrations in aeration tank effluent (SP4) and RAS (SP5) were higher than in SP1 and SP3 after the three-day injection period and remain at least 2 log_10_ units higher through the rest of the experiment (Fig 4). *B*. *globigii* concentrations decreased in the aeration basin and effluent slightly during the SRT range, but visually began rising slightly afterward (Fig 3).

Several lines of evidence suggest that *B*. *globigii* has colonized components of the AST. (i) Concentrations of *B*. *globigii* were statistically significantly higher in the aeration basin, RAS, and secondary clarifier compared to sample ports at the primary clarifier (F Table in S1 Text). To summarize, concentrations reached a mostly steady state condition after the SRT range. These data were averaged and compared using 2-sample T-tests to see if the sustained levels of concentration were significantly different from each other because significant differences when comparing samples from different parts of the AST system could also pinpoint the stage of the apparatus that was colonized. F Table in S1 Text shows significant differences (p<0.05) between the sample ports associated with the primary clarifier (SP1 and SP3) and the aeration basin sample ports (SP4 and SP5) and between the aeration basin ports and the system effluent (SP6), suggesting that the apparent greater concentration of *B*. *globigii* persisting beyond the SRT in the aeration basin, RAS, and secondary clarifier effluent is not a result of random chance. The higher concentrations of *B*. *globigii* at SP4 and SP5 suggest that the aeration basin and RAS are the likely origins of new *B*. *globigii* growth in the system. (ii) The concentration of *B*. *globigii* in the aeration basin and RAS visually increased slightly after the latest possible SRT (Fig 3, after t = 23 days, in experiment 1). This suggests that the rate of surrogate replacement in those locations matches or exceeds its rate of removal. (iii) System effluent (SP6) surrogate concentrations were slightly less than those in the aeration basin (SP4 and SP5) until the end of experiment monitoring.

The above data and discussion alone do not establish whether *B*. *globigii* persistence is a function of colonization or another mechanism. Since the RAS (SP5) was also significantly greater than the primary clarifier sample ports, accumulation in the recycled material and aeration basin cannot be ruled out. Sometimes higher TSS levels were observed in samples with greater persistence of *B*. *globigii* (Fig 4), but statistical analysis did not necessarily substantiate this trend.

If the *B*. *globigii* spores moved through the system without germination, colonization, or accumulation, there should be a time when SP6 surpasses the other sample ports for *B*. *globigii* concentration, but it remains consistently lower than concentrations in the aeration chamber and RAS (SP4 and SP5). There are several possible reasons for this. (i) Some of the surrogates may be retained and accumulate in the aeration chamber and RAS due to the system’s flow patterns. (ii) *B*. *globigii* could be germinating and reproducing in the aeration chamber and RAS, becoming a constant source for *B*. *globigii*. (iii) *B*. *globigii* is released from the aeration chamber in bioaerosols. Bioaerosol experiments conducted with the AST apparatus [22] indicated that this mechanism would make up an insignificant percentage of the difference between the aeration tank (SP4) and the secondary clarifier effluent (SP6). There is insufficient *B*. *globigii* data to alone determine whether the accumulation or germination are taking place.

### 3.3 Fate of MS2 in AST system

Figs 5 and 6 show the response data for MS2. No MS2 was detected before the start of the experiment. The response curves of measured MS2 followed a similar trend across all sampling ports with slight concentration differences during the first MS2 study, which could have been a result of random variability (Fig 5). As indicated in Table 1, the MS2 suspension was prepared in dechlorinated tap water for the first experiment and in synthetic wastewater (SWW) for the second experiment. This was to account for potential changes resulting from water quality characteristics that may change MS2’s adsorption behavior to colloids [23]. Fig 6 shows the persistence of MS2 relative to SRT and the TSS levels.

The variances in MS2 concentration among the sampling ports were diminished during the second MS2 study. MS2 concentrations were at or below the limit of detection after the maximum possible SRT, so there were no significant differences (p-values > 0.05) revealed by the t-tests between sample port results (F Table in S1 Text). MS2 appeared to be more evenly distributed through the system than *B*. *globigii*, and it appeared to have mostly left the AST system by the end of the possible SRT. Preparing MS2 in the SWW from the rest of the system may have facilitated more rapid mixing with the rest of the SWW during the first 5 days, but there was no significant difference in surrogate persistence after SRT.

MS2 concentrations in the AST system increased to around the target concentration of ~10^7^ PFU/100 mL within four hours (Figs 5 and 6). MS2 concentrations dropped sharply after the first day even though MS2 stock was still being added. In Fig 6, the SP1 and SP3 ports are averaged as the “primary clarifier” series and SP4 and SP5 are averaged as the “aeration basin.” MS2 experiments 1 and 2 results were not averaged because of the difference in surrogate preparation method from *B*. *globigii* and Phi-6. However, the RAS and aeration basin TSS values are averaged between experiment 1 and experiment 2 because the AST’s hydraulic and composition conditions remained constant between the two experiments.

MS2 was at or near the detection limit by the end of the SRT range (28.9 days) in all parts of the AST in contrast to *B*. *globigii*’s long persistence beyond SRT. MS2 concentrations were negligible (10^0^−10^1^ PFU/100 mL) after 14 days during the first MS2 experiment and at 35 days in the second experiment (Fig 5). These time values are based on when negligible levels were visually judged to occur across all sampling ports, so the exact time is subject to interpretation. Levels were highest in the RAS (SP5) and system effluent (SP6) during the second MS2 test. The t-test showed that the use of synthetic wastewater instead of dechlorinated water had no significant effect on the concentrations of MS2 in the system after the SRT (P-value = 0.160; F Table in S1 Text).

TSS levels in the RAS and aeration basin were not observed to correlate with MS2 concentration in any part of the AST. TSS levels spiked in the aeration basin effluent in two places: once near the beginning and once at the end of the possible SRT range. No cause was determined and MS2 levels seemed unaffected. This suggests that MS2 may not adsorb to suspended solids, which fits with findings of Espinosa et al. (2021) [24]. Literature seems to be mixed depending on the types of solids being studied, however, as Xing et al. (2020) saw rapid adsorption of MS2 to kaolin colloids [23].

No significant difference was detected between the primary clarifier sample ports (SP1 and SP3) and the aeration basin sample ports (SP4 and SP5), suggesting that no colonization or accumulation was evident in the AST system.

### 3.4 Fate of Phi-6 in AST system

The Phi-6’s 10 times lower suspension titer, shorter inoculation period and slower hydraulic flow rate changed many operating parameters of the AST system. No Phi-6 was detected prior to its addition. Response curves of Phi-6 rose and fell over a shorter period than the previous surrogates (Fig 7). The averaged data shows the highest concentration of Phi-6 associated with the primary clarifier and that the levels dropped below the detection limit for all locations ~13 days before the calculated SRT range (Fig 8). The second test was finished after day 2. Spiked Phi-6 concentrations in the AST system reached the expected concentration of approximately 10^6^ PFU/100 mL within eight hours, but only at SP1 (Fig 7). Peak Phi-6 concentrations in other parts of the AST at the same time were two to three orders of magnitude lower than SP1.

Hydrophobic viruses like Phi-6 and other enveloped viruses are expected to have a stronger affinity for certain wastewater solids than other viruses like MS2 [24]. However, whether as a solid particle itself or associated with other solids, Phi-6 removal correlated poorly with HRT and SRT, suggesting that their persistence is not governed by those flow properties (Fig 8). There was no significant correlation with average TSS levels in the RAS or aeration basin effluent observed. Previous studies like Aquino de Carvalho et al. (2017) [8] suggest that the temperature may partly explain the faster Phi-6 removal. At room temperature T_90_ (time for 90% inactivation) for Phi-6 in autoclaved wastewater is about 2.5 days without other inactivation treatment.

If 90% of Phi-6 is inactivated within 2.5 days at room temperature, the population in the present experiments could reach undetectable levels from 10^7^ CFU/100 mL within about 12 days. With average recorded temperatures of 27.7°C and 25.3°C for experiments 1 and 2 respectively, the measured Phi-6 concentrations dropped more quickly than the expected rate of inactivation, being negligible mostly by day 4 (Figs 7 and 8).

### 3.5 Implications and factors influencing persistence among pathogen surrogates

#### 3.5.1 Implications

The main objective of this study was to determine and compare the persistence of *B*. *globigii*, MS2, and Phi-6 surrogates in AST systems. All surrogates persisted beyond the hydraulic retention time of the AST system, indicating HRT does not govern pathogen persistence. Persistence of some spore forming bacteria beyond the SRT suggests either colonization or accumulation. Since MS2 did not also persist beyond SRT, accumulation seems a less likely explanation for *B*. *globigii* persistence.

#### 3.5.2 Effect of AST operational parameters

The concentration of *B*. *globigii* in the Aeration basin effluent (SP4; MLSS) and the system effluent (SP6) correlated to aeration basin effluent TSS (MLSS) with a linear regression R^2^ of 0.45 and a p-value of 3.2×10^−4^ (Section 6 of S1 Text). System effluent (SP6) *B*. *globigii* and TSS were also correlated (R^2^ = 0.19, p-value = 0.032). Although other correlation analyses between *B*. *globigii* concentration and TSS showed no significant relationship (p > 0.05) in other parts of the AST system, the results suggest some relationship between the two. Further, there were no such relationships found in the MS2 studies, perhaps suggesting a difference in the interaction mechanism between MS2 and solids which is not unexpected, but more research would be needed to be conclusive. Indeed, Espinosa et al. (2021) [24] notes that viruses can have high negative charge, potentially limiting their adsorption to some types of particles. There was insufficient data to attempt this analysis for Phi-6.

Temperature-based inactivation of these three surrogates is well documented. Anders and Chrysikopoulos [25] documented that ~90% MS2 inactivation happens after more than 120 days at 5°C and between 10 and 20 days at 30°C, which likely did not significantly affect MS2’s persistence in the pilot system. Lian et al. [26], showed that temperature modestly influenced MS2 inactivation by UVB light treatments. Temperature also inactivates *Bacillus* spores, but usually only at temperatures well above 30°C [27, 28]. Accordingly, it is generally understood that, for the temperature range in this study (20 to 27° C), only Phi-6 persistence should be significantly affected by the ambient temperature. This study’s observations are consistent with temperature dependence from other Phi-6 studies [8, 29].

#### 3.5.3 Bioaerosol formation

While an important pathway from a wastewater worker and surrounding area health and safety perspective, bioaerosol release likely accounts for an insignificant percentage of the surrogate population as was suggested in the results from Xing et al. (2021) [22]. Bioaerosols accounted for less than 1×10^−5^ to around 1% of the *B*. *globigii* in the entire system depending on potential bioaerosol sizes. Such airborne emissions from an aeration tank consist of both bioaerosols and droplets that are too large to be bioaerosols [30].

#### 3.5.4 Comparative persistence of bacterial and viral surrogates

*B*. *globigii* concentrations stabilized at concentrations between 10^5^ to 10^6^ CFU/100mL after the calculated SRT in the aeration tank (SP4) and return activated sludge (SP5). Alone, this suggests either accumulation or colonization, according to its ability to grow in AST systems [31]. MS2 at the SRT shows the more expected behavior of a surrogate that does not colonize and grow in the AST system, which is not unexpected since its host is not expected to be present in significant quantities. It also shows limited accumulation of MS2, which points to colonization as an explanation of *B*. *globigii*’s persistence. If this small system can be colonized, so can large-scale systems. At the system effluent port (SP6) for *B*. *globigii* (Figs 3 and 4), overall AST system removal was approximately 4 log_10_ units despite the persistence of higher concentrations of *B*. *globigii* in the aeration tank and RAS.

The TSS concentration remained higher in the RAS and aeration basin effluent than in the other three sample ports near the end of both MS2 experiments and Phi-6 experiments (G Fig in S1 Text). Had surrogates been attaching to those solids MS2 concentrations in SP4 would have remained higher than at other sample ports. This further supports the hypothesis that the *B*. *globigii* persistence is more likely from colonization of the AST system.

Despite experimental artifacts and uncertainties, Phi-6 experiments reached a point of zero net-change at ~7 days, while MS2 reached a point of zero net-change at ~14 days. There are several factors that may contribute to the persistence results for the viruses. First, while some viruses might be expected to occur naturally in the inoculum used to seed the AST system, it is understood that coliphages do not often replicate in the wastewater, explaining their steady decline to detection limits [32].

A second factor is the role of fate and transport mechanisms related to association of the viruses with particles in the wastewater. If association occurs, their transport out of the AST system would be expected to be related to the solid retention time. In the present study, Phi-6 concentration dropped to negligible levels faster than the other surrogates, despite a comparable SRT. The lower sludge wasting rates during the Phi-6 tests likely compensated for those experiments’ slower flow rates in the SRT calculations. Sludge may have been more likely to settle out during the Phi-6 test, as indicated by comparatively lower effluent TSS (G Fig in S1 Text), although the tendency of mixed liquor to settle out or sludge volume index was not determined during any of the tests.

It was apparent that another factor affected the persistence of Phi-6 besides SRT. Otherwise, its persistence would likely be around 20 days, dropping to negligible levels by approximately 30 days, reflecting its range of calculated SRT. However, even though the SRT range was somewhat longer in the Phi-6 experiments than MS2’s SRT range, Phi-6’s persistence was much shorter. Relatively low temperatures have been found to inactivate Phi-6 [8] and may explain the short-lived persistence in our study.

## 4. Conclusions

This study provides insights into appropriate management of liquid and solid waste streams from the activated sludge treatment process to prevent spread of high consequence pathogens in the environment and potential worker exposure, the main concerns of the WRF report [6]. This study demonstrated that even short-term exposure of sporulating aerobic bacteria like *B*. *anthracis* could lead to the aeration chamber and RAS becoming long-term sources of pathogenic spores. Those pathogens can re-enter the environment by contaminating biosolid wastes from the AST system and are recalcitrant to secondary disinfection prior to environmental discharge. Further work should develop approaches to decontaminate a colonized system and to extend these results to other species of sporulating bacteria.

Secondary wastewater treatments can be effective for enveloped viruses, and while non-enveloped viruses can survive such treatments [32], the likelihood of the AST system becoming a source of viral pathogens is lower than that for spore forming bacteria like *B*. *anthracis*. The results for MS2 suggest that any necessary supplemental disinfection treatment should be applied for a length of time greater than the SRT after a contamination incident. Phi-6 data suggests that some pathogens’ persistence may depend on other factors.

## Supporting information

S1 Text Supplemental methods, figures, photos, and statistical analysis data and output. (DOCX)

S2 Text Compilation of original data. (DOCX)
