# Supplementary material for: Persistence of surrogates for high consequence viral and bacterial pathogens in a pilot-scale activated sludge treatment system
Source: PLoS One. 2022 Oct 7;17(10):e0275482. doi: 10.1371/journal.pone.0275482 (PMC9543761; doi:10.1371/journal.pone.0275482)
Supplement: S1 Text — (DOCX) [file pone.0275482.s001.docx]

# S1 Text: Supplemental Methods, Figures, Photos, and Statistical Analysis Data and Output

**Persistence of Surrogates for High Consequence Viral and Bacterial Pathogens in a Pilot-Scale Activated Sludge Treatment System**

Donald A. Schupp^,1,‡^, Adam C. Burdsall*^2^, Rendahandi G. Silva^1,‡^, J. Lee Heckman^1^, E. Radha Krishnan^1^, Jeffrey G. Szabo^2^, and Matthew Magnuson^2^

1. APTIM Federal Services, 1600 Gest St., Cincinnati, OH 45204

2. U.S. Environmental Protection Agency, Office of Research and Development, Center for Environmental Solutions and Emergency Response, Homeland Security and Materials Management Division, 26 W. Martin Luther King Dr., Cincinnati, OH 45268

*Corresponding Author

‡Contributed equally

# Synthetic Wastewater Composition and use

The second MS2 experiment was conducted using a synthetic wastewater stock solution prepared in 3.2 L of 70°C deionized water with the composition below in A Table. This solution was mixed with 40.0 L of dechlorinated tap water, which could feed the Activated Sludge Treatment (AST) system for 5 days.

**A Table: Composition of Synthetic Wastewater Solution**

| **Material** | **Chemical Formula** | **Approximate Influent  Concentration (mg/l)** |
| --- | --- | --- |
| *Organic Compounds* |  |  |
| Casein | NA | 47.0 |
| Tryptone | NA | 47.0 |
| Starch | NA | 84.4 |
| Sodium Acetate | C_2_H_3_O_2_Na.3H_2_O | 31.9 |
| Glycerol | C_3_H_8_O_3_ | 12.0 |
| Caproic Acid | C_6_H_12_O_2_ | 11.6 |
| *Nutrients* |  |  |
| Ammonium Sulfate | (NH_4_)_2_SO_4_ | 116.0 |
| Magnesium Sulfate | MgSO_4_.7H_2_O | 69.6 |
| Calcium Chloride | CaCl_2_.2H_2_O | 22.5 |
| Potassium Phosphate | K_2_HPO_4_ | 27.6 |
| Iron Chloride | FeCl_3_ | 11.0 |
| Cupric Sulfate | CuSO_4_.5H_2_O | 0.09 |
| Sodium Molybdate | Na_2_MoO_4_.2H_2_O | 0.15 |
| Manganese Sulfate | MnSO_4_.H_2_O | 0.13 |
| Zinc Chloride | ZnCl_2_ | 0.23 |
| Cobalt Chloride | CoCl_2_.6H_2_O | 0.42 |

# AST system stability:

Before each experiment the AST system was decontaminated using a 25% bleach solution in tap water. The AST was disassembled, and each component was cleaned with tap water followed by bleach solution. The AST was reassembled and drenched with bleach solution for three days prior to operating the system for thirty minutes. The AST was rinsed thoroughly with dechlorinated tap water and drained to remove residual bleach from the system. Prior to each experiment, the AST was reset to baseline. The AST system was colonized using return activated sludge (RAS) from the Metropolitan Sewer District of Greater Cincinnati Mill Creek wastewater plant. The AST system was operated for two to three weeks after colonization to stabilize and establish the baseline operating conditions. Operations utilized SWW, the composition of which can be found in A Table above.

The stability of the AST system was assessed by monitoring chemical oxygen demand (COD) and ammonia concentrations in the AST system influent and effluent during baseline operation. The target COD of the SWW influent was maintained at approximately 300 mg/L during each test. The pilot-scale AST system was considered to be operating efficiently when COD and/or ammonia influent concentrations were reduced by 70-90% in relation to the final effluent. The sludge recycling and wasting rates were adjusted for the different influent flow rates, depending on the system performance.

The performance of all AST systems is influenced by the activated sludge sample used to colonize the system. The characteristics of the colonizing sample change over time based on changes in the full-scale plant from which it was obtained. Thus, it is necessary to allow the pilot scale AST system to stabilize to equivalent, acceptable performance, despite differences in the colonizing sample. B Table documents the stability and acceptable performance of the AST system across the three surrogate conditions.

B Table: Sludge Retention Time calculations

| Time (d) | V (L) | RAS | MLSS mg/L | W (L/d) | SRT (d) | RAS | MLSS mg/L | W (L/d) | SRT (d) | RAS | MLSS mg/L | W (L/d) | SRT (d) |
| --- | --- | --- | --- | --- | --- | --- | --- | --- | --- | --- | --- | --- | --- |
|  | (Aeration basin + secondary clarifier) | (TSS (mg/L) *B. globigii* test 1) | | Wasting rate |  | (TSS (mg/L) MS2 test 2) | | Wasting rate |  | (TSS (mg/L) Phi-6 test 1) | | Wasting rate |  |
| 1 | 265 | 600 | 1548 | 43.2 | 15.8 | 647 | 946 | 28.8 | 13.5 | 873 | 1250 | 14.4 | 26.3 |
| 2 | 265 | 572 | 1702 | 43.2 | 18.3 | 789 | 822 | 43.2 | 6.4 | 840 | 1017 | 14.4 | 22.3 |
| 3 | 265 | 563 | 1306 | 28.8 | 21.3 | 637 | 818 | 72 | 4.7 | 848 | 955 | 14.4 | 20.7 |
| 4 | 265 | 552 | 652 | 28.8 | 10.9 | 517 | 840 | 28.8 | 15.0 | 859 | 1021 | 14.4 | 21.9 |
| 5 | 265 | 228 | 659 | 28.8 | 26.6 |  |  |  |  | 835 | 1135 | 14.4 | 25.0 |
| 6 | 265 | 208 | 493 | 21.6 | 29.1 |  |  |  |  |  |  |  |  |
| 7 | 265 | 246 | 486 | 21.6 | 24.2 |  |  |  |  |  |  |  |  |
| 8 | 265 | 222 | 647 | 21.6 | 35.8 | 460 | 757 | 57.6 | 7.6 | 852 | 1090 | 14.4 | 23.5 |
| 10 | 265 | 195 | 428 | 21.6 | 26.9 |  |  |  |  |  |  |  |  |
| 15 | 265 | 298 | 789 | 28.8 | 24.4 | 290 | 331 | 57.6 | 5.3 |  |  |  |  |
| 17 | 265 | 232 | 538 | 28.8 | 21.3 |  |  |  |  |  |  |  |  |
| 22 | 265 | 234 | 378 | 21.6 | 19.8 | 158 | 340 | 14.4 | 39.6 |  |  |  |  |
| 24 | 265 | 182 | 331 | 21.6 | 22.3 |  |  |  |  |  |  |  |  |
| 29 | 265 | 277 | 570 | 21.6 | 25.2 | 311 | 467 | 57.6 | 6.9 |  |  |  |  |
| 31 | 265 |  | 301 | 21.6 |  |  |  |  |  |  |  |  |  |
| 35 | 265 | 150 | 258 | 21.6 | 21.1 | 238 | 397 | 21.6 | 20.5 |  |  |  |  |
| 38 | 265 | 135 | 248 | 21.6 | 22.5 |  |  |  |  |  |  |  |  |
| 43 | 265 | 109 | 289 | 21.6 | 32.5 | 330 | 525 | 21.6 | 19.5 |  |  |  |  |
| 45 | 265 | 186 | 415 | 21.6 | 27.4 |  |  |  |  |  |  |  |  |
| 50 | 265 | 333 | 680 | 21.6 | 25.1 | 222 | 482 | 21.6 | 26.6 |  |  |  |  |
| 52 | 265 | 354 | 606 | 21.6 | 21.0 |  |  |  |  |  |  |  |  |

*Performance of the AST System*

The percent COD and ammonia removal efficiencies determined using system influent and the final effluent are presented in A, B, and C Figs. for *B. globigii*, MS2, and Phi-6 tests, respectively. The average COD removal efficiencies were approximately 70%-80% for the *B. globigii*, MS2, and Phi-6 tests. The percent ammonia removal efficiencies indicated that the average ammonia removal during the *B. globigii* experiments was approximately 35% and lower than the average ammonia removal of 50% during the MS2 and Phi-6 surrogate pathogen studies. Despite some random variabilities, there were no meaningful differences in COD removal between the two tests for a given surrogate pathogen compared to ammonia (A – C Figs.). In terms of ammonia removal efficiencies, the system performance was lower than anticipated except for the first MS2 surrogate pathogen test. The existing differences among tests could have resulted from different lengths of experiments, as well as varying sequences of sampling.


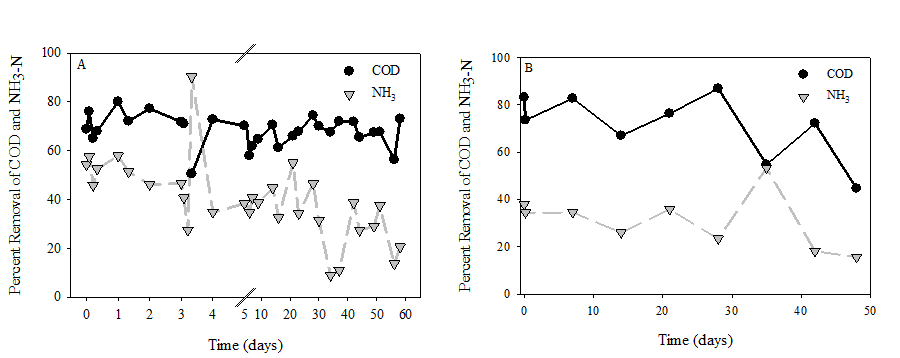


**A Fig. Percent COD and ammonia removal** during *Bacillus globigii* Test 1 (left) and Test 2 (right).


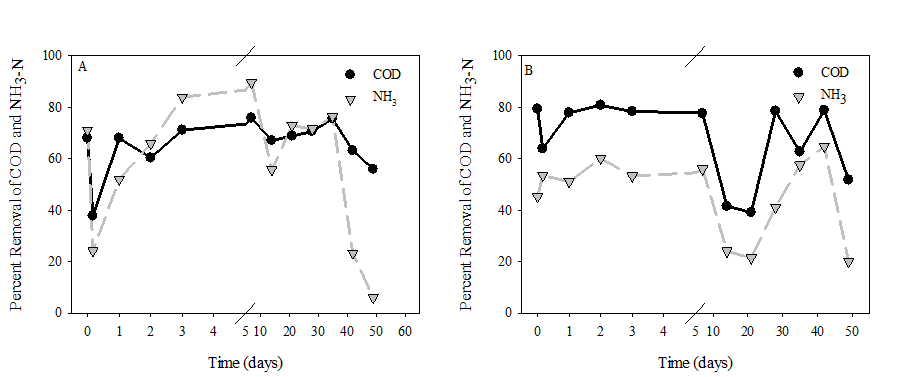


**B Fig.** **Percent COD and ammonia removal** during MS2 Test 1 (left) and Test 2 (right).


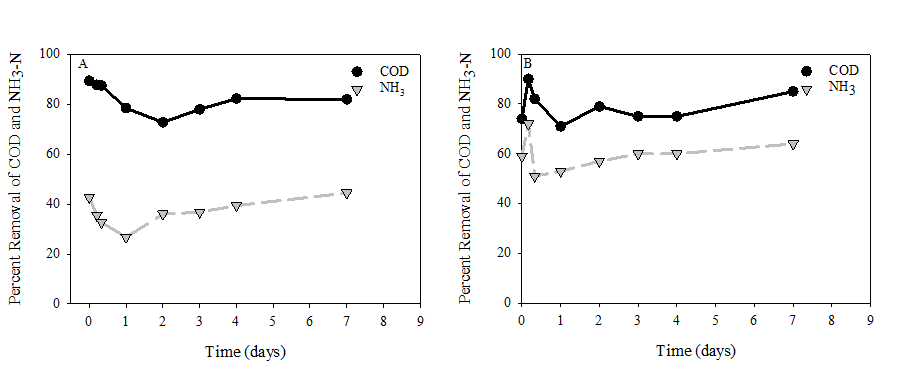


**C Fig.** **Percent COD and ammonia removal** during Phi-6 Test 1 (left) and Test 2 (right).

The TSS concentrations measured in both the mixed liquor suspended solids (SP4) and the waste (SP5) during each test are presented in B Table. Generally, the measured VSS concentrations were 80% – 85% of the TSS. As mentioned in the manuscript, *B. globigii* Test 1 had a frequent sampling schedule. Although the data in B Table have been presented on a sampling day basis, samples from the other three tests were collected on a weekly basis after the first week. The Phi-6 tests lasted only eight days since complete removal of Phi-6 was observed within four days.

The temperature fluctuation was between 20-27 ºC, depending on when the experiment took place. The average DO concentrations of the AST system were approximately 5.0 mg/L except during the first *B. globigii* (2.6 mg/L) test. Overall, the physicochemical water quality parameters among the surrogate pathogen tests were not meaningfully different despite some random variabilities (A – C Figs. and B Table). The AST system used SWW during these studies. The AST system, however, managed to efficiently remove representative water quality parameters, in line with large-scale wastewater treatment^2^.

We speculate that the slight variation in system water quality parameters during each experimental phase was due to the complex nature of the sample matrices and physical limitations, including occasional bulking of the sludge. The delivery of RAS and effluent through the peristaltic pump system were changed to meet experimental needs. The slightly lower airflow (20 L/min) maintained during the first test was somewhat lower than airflow (25 L/min) during other tests, to maintain appropriate DO concentrations. However, an important consequence of comparison of this duplicate surrogate pathogen test series is that the variable inflow and outflow did not meaningfully affect the performance of the activated sludge process. Thus, the different removal efficiencies of COD and ammonia, as well as concentrations of TSS and VSS, during various sampling stages of the activated sludge system were attributed to random sampling variability rather than system attributes. The ratio of VSS to TSS was comparable and consistent. Although environmental conditions (e.g., temperature) may play a role in activated sludge system performance, experimental conditions for each surrogate pathogen test were otherwise similar.

Solid retention time was calculated according to the formula for SRT described in Grady et al.^7^. The formula for SRT is as follows:

$SRT= \left( \frac{V\times MLSS}{W\times RAS} \right)$

where V = volume of aeration tank, W = waste rate L/min (B Table), MLSS = aeration tank outlet concentration of total suspended solids (TSS) into clarifiers (B Table), and RAS = Return sludge (waste sludge) concentration of TSS out of clarifiers (B Table). SRT ranges observed in several literature resources is in C Table.

**C Table:** Examples of other studies’ solid retention times to compare to those of the AST.

| **Study** | **Name/Characteristics of facility** | **Suspended Solid Concentration (mg/L)** | **Sludge retention time (days)** |
| --- | --- | --- | --- |
| [2] | Vroomshoop | 3-14 | 21 to 27 |
| [2] | Gamerwolde | 5-14 | 23 to >30 |
| [3] | Primary settling + Activated sludge system | 4000 | 2 |
| [3] | Activated sludge system + Anaerobic sludge digestion | 5900 | 19 |
| [3] | Primary settling + Activated sludge system + Anaerobic sludge digestion | 3100 | 48 |
| [4] | Trickling filter system with primary and secondary clarifiers | 133.3 influent,  46 effluent | 0.04 to 0.08 |
| [4] | Wastewater stabilization ponds with screening and grit removal | 111.3 influent,  53.5 effluent | 3.5 days in anaerobic ponds,  Other ponds: 1460 |
| [4] | Balancing tank, settling tank, and anaerobic baffled reactor with a constructed wetland | 139.3 influent,  72.8 effluent | 14 |
| [5] | Conventional plug flow  Modified aeration (similar to plug flow)  Step feed  Extended Aeration  High-rate aeration | 1000-3000 (MLSS)  200-1000 (MLSS)  1500-3500 (MLSS)  1500-5000 (MLSS)  3000-6000 (MLSS) | 3-15^a^  0.2-0.5  3-15  20-30  5-10 |
| [6] | Desired SRT for a conventional WWTP | 1000-4500 (MLSS) | 5-15 days |

^a^SRT of 3 to 15 days produces a stable effluent and sludge with favorable settling characteristics

# Color figure versions of Manuscript figures with plots of their derivatives

**
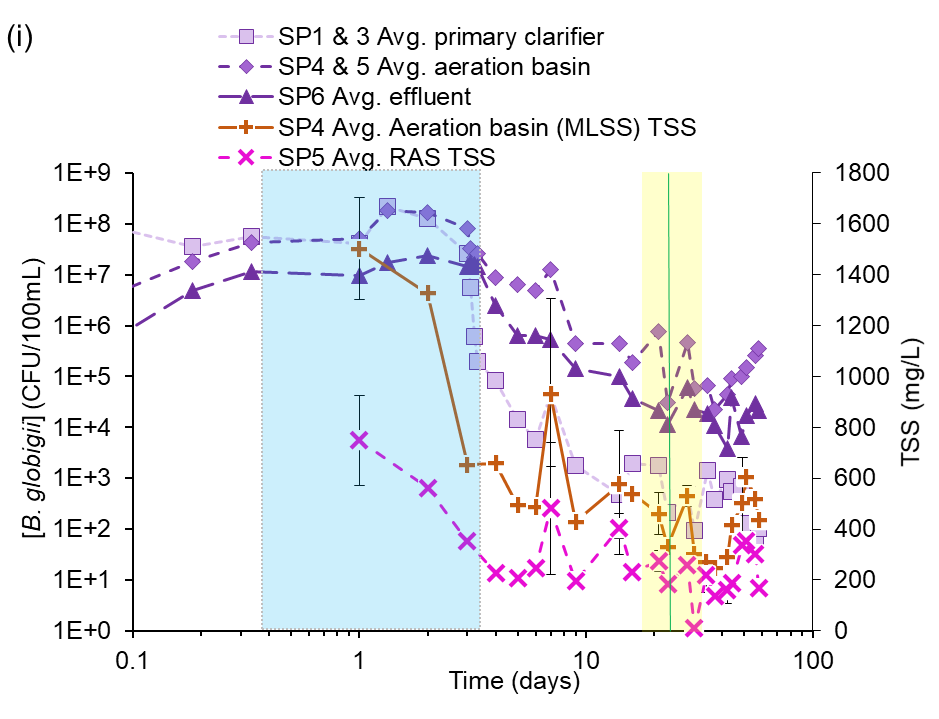
**

**
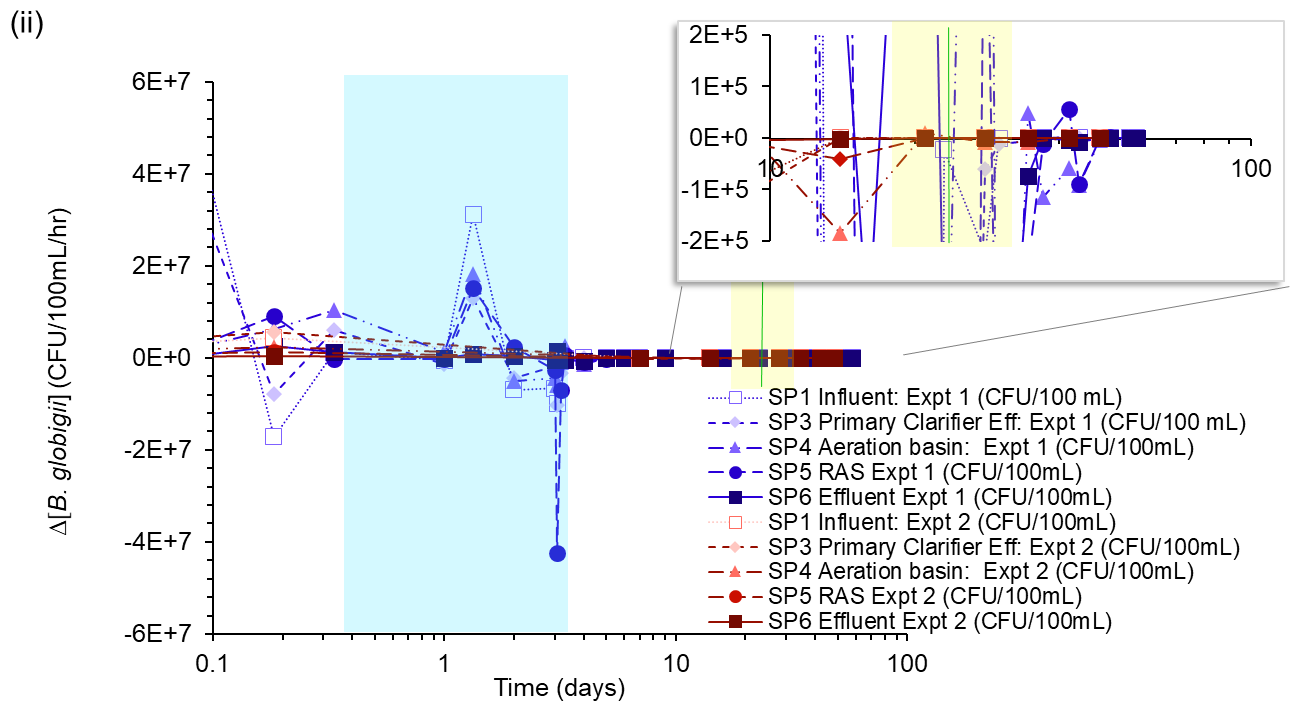
**

**D Fig. Uncropped color version of Fig. 3 and derivative graph.** Part i shows the individual sample port *B. globigii* concentrations over time on a log-log plot. Part ii shows the derivative of the data shown in part i, depicted as a change (Δ) in *B. globigii* concentration. After 10 days, the Δ[*B. globigii*] is too small to be seen at the scale on the y-axis, so an inset magnifies the data after 10 days to show the small changes in surrogate concentration. HRT and SRT ranges are shown in both figures by the light blue and yellow bars, respectively. The green line is the specific calculated SRT estimate.


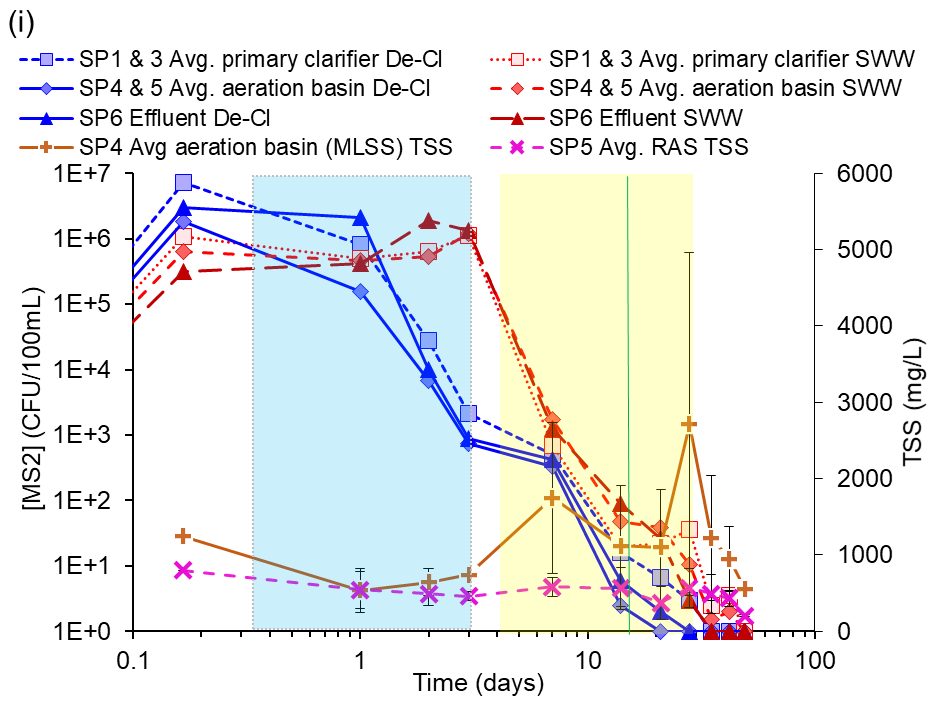


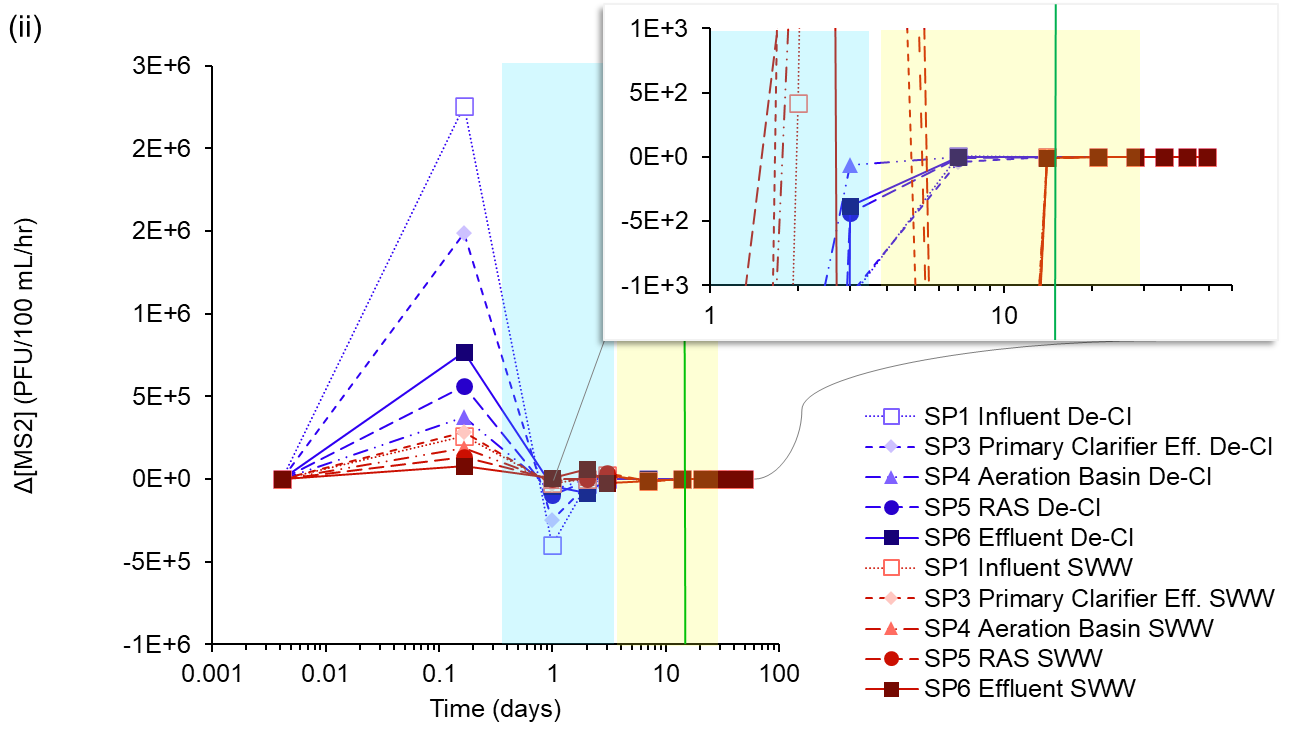


**E Fig. Uncropped color version of Fig. 5 and derivative graph.** Part i shows the individual sample port MS2 concentrations over time on a log-log plot. Part ii shows the derivative of the data shown in part i, depicted as a change (Δ) in MS2 concentration. After 10 days, the Δ[MS2] is too small to be seen at the scale on the y-axis, so an inset magnifies the data after 10 days to show the small changes in surrogate concentration. HRT and SRT ranges are shown in both figures by the light blue and yellow bars, respectively. The green line is the specific calculated SRT estimate.

When interpreting the Phi-6 results, please note that influent Phi-6 concentrations were an order of magnitude lower than the influent concentrations of the other two surrogates and that since the maximum possible stock concentration of Phi-6 was 108 PFU/100 mL, Phi-6 was directly injected from the stock solution in which it was prepared.


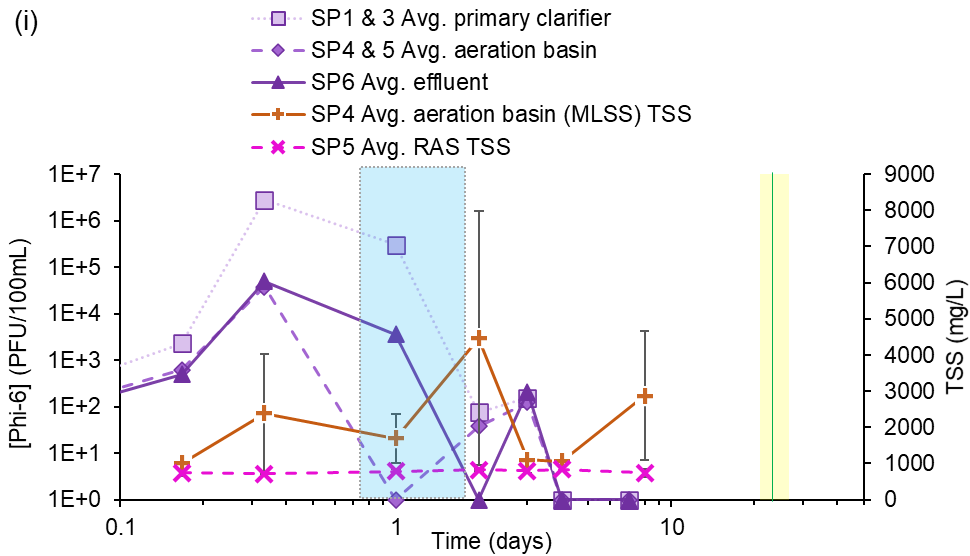


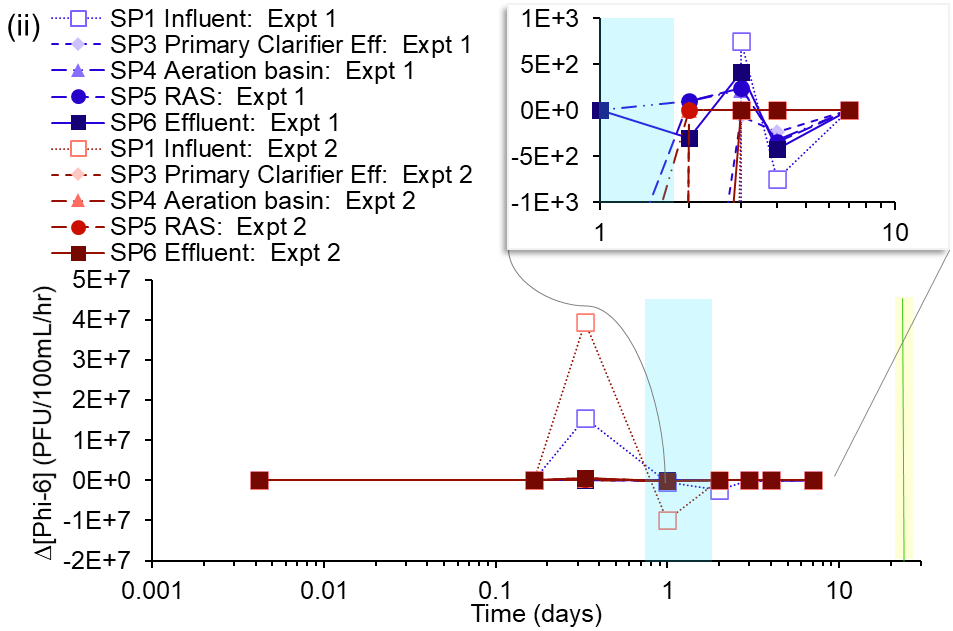


**F Fig. Uncropped version of Fig. 7 and derivative graph:**  Part i shows the individual sample port Phi-6 concentrations over time on a log-log plot. Part ii shows the derivative of the data shown in part i, depicted as a change (Δ) in Phi-6 concentration. After 10 days, the Δ[Phi-6] is too small to be seen at the scale on the y-axis, so an inset magnifies the data after 10 days to show the small changes in surrogate concentration. HRT and SRT ranges are shown in both figures by the light blue and yellow bars, respectively. The green line is the specific calculated SRT estimate.

# Total Suspended Solids Temporal Variation: Comparison among Surrogates


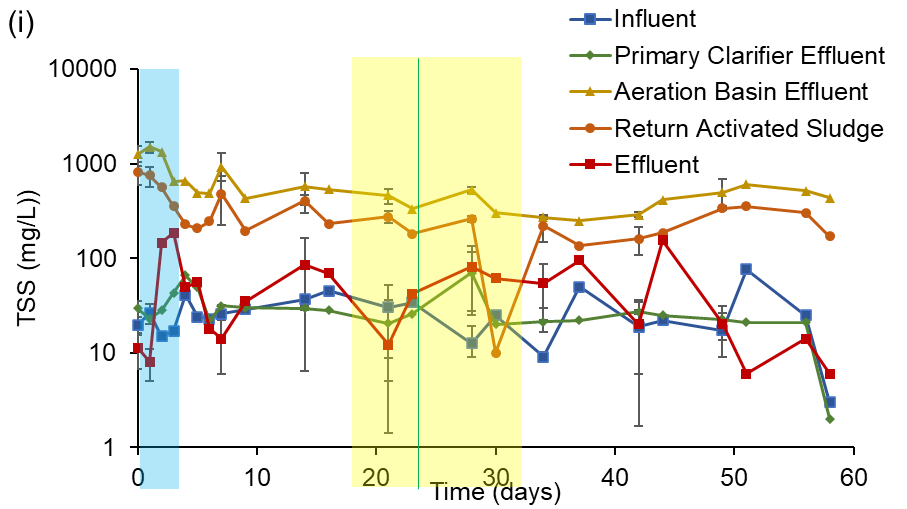


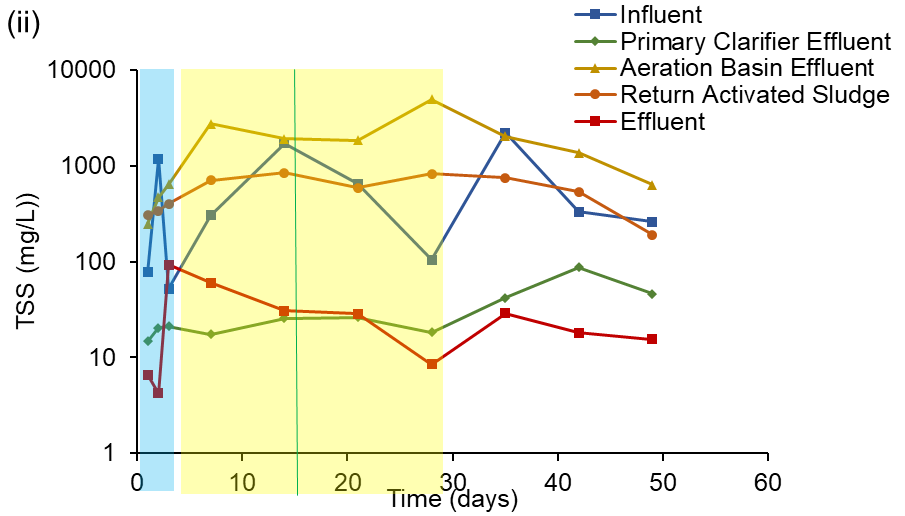


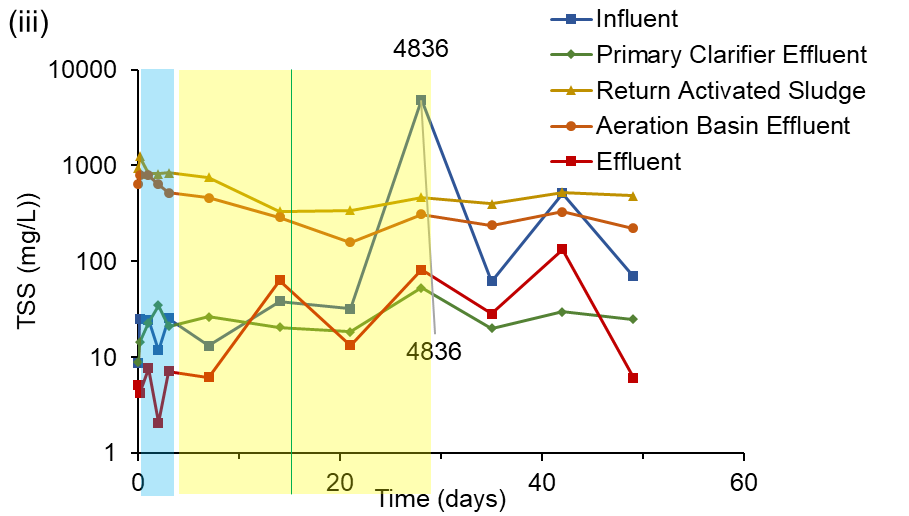


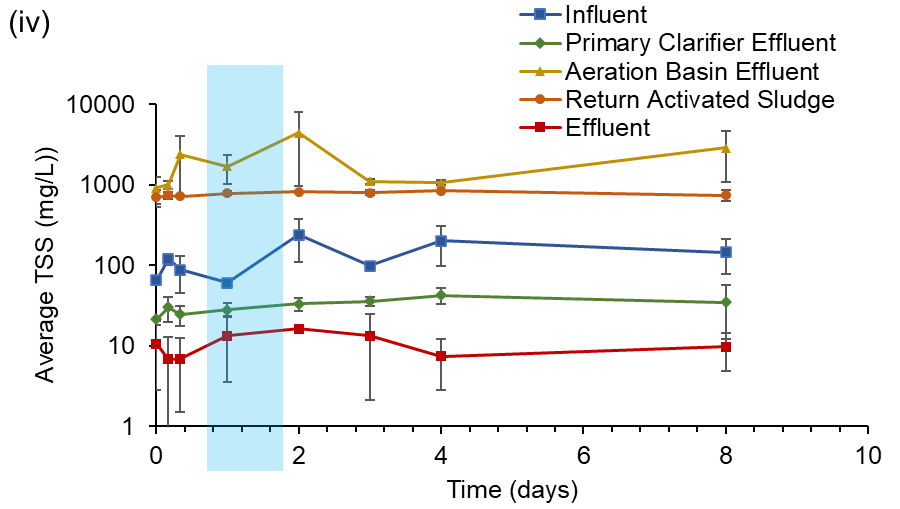


**G Fig. TSS concentrations** showing (i) average TSS at each time point from two *B. globigii* experiments, (ii) TSS during MS2 experiment 1 using dechlorinated tap water in the MS2 stock, (iii) TSS during MS2 experiment 2 using AST wastewater in the MS2 stock, and (iv) average TSS at each time point from two Phi-6 experiments. Error bars represent the range of the data when data was the average of 2 or more samples or experiments. There are no error bars on (ii) and (iii) because each data point represents one sample. The HRT range is represented by the light blue block. The calculated SRT value is represented by a green vertical line. The possible range is a yellow block.

# Testing the statistical significance of the data

The concentrations of surrogates eventually reached a steady state condition. The steady state condition was reached, usually during the possible range of the SRT for the *B. globigii* and MS2 experiments. The data points that were collected at each location after the latest possible SRT for all experiments (except Phi-6 experiments) were used for the data analysis. A 2-sample T-test as implemented in Excel was used to determine whether the average concentration of surrogate at that location after the end of the possible SRT was significantly different from the same range of data collected from another location in the AST system. The summary of the analysis is shown in the data below and summarized in F Table at the end of the section.

**D Table: Raw data from the first *B. globigii* experiment for Significance Tests.**  This data consists of the *B. globigii* concentrations, in CFU/100mL, data collected after the SRT’s possible range (t=32 days). P values used to determine the significance of the data are highlighted. Non-significant results (p > 0.05) are highlighted in red. Significant results at the 95% confidence level are highlighted in green. Results that are not statistically significant at the 95% confidence level, but would be significant at a lower confidence level (0.05 <p < 0.10) are highlighted in yellow.

| time d | SP1 Avg. Primary clarifier influent | SP3 Avg. Primary clarifier effluent | SP4 Avg. Aeration Basin effluent | SP5 Avg. RAS | SP6 Avg. Secondary Clarifier effluent |
| --- | --- | --- | --- | --- | --- |
| 34.5 | 2550 | 762.5 | 75500 | 80000 | 31500 |
| 37 | 480 | 270 | 23000 | 22000 | 10600 |
| 42 | 35 | 120 | 25000 | 18000 | 4366.7 |
| 44 | 110 | 1005 | 125000 | 59500 | 38000 |
| 48.5 | 100 | 450 | 210000 | 115000 | 9533.3 |
| 51 | 200 | 60 | 225000 | 80000 | 17000 |
| 56 | 50 | 50 | 250000 | 270000 | 29000 |
| 58 | 150 | 52.5 | 250000 | 460000 | 21500 |

**D Table : 2-sample T-tests between sample ports:** Experiment 1 Calculations, Part 2

| 2-sample T-tests assuming unequal variances: Testing for significance between sample ports | | | | | | | | | | | | | | | | | | |  | | |  |
| --- | --- | --- | --- | --- | --- | --- | --- | --- | --- | --- | --- | --- | --- | --- | --- | --- | --- | --- | --- | --- | --- | --- |
|  | *SP1* | *SP3* | |  | | *SP4* | | *SP5* | | |  | | | | *SP4* | | *SP6* | | | |  |  |
| Mean | 459.4 | | 346.3 | | Mean | | 1.5E+05 | | 1.4E+05 | | |  | | Mean | | 1.5E+05 | | 2.0E+04 | | | |  |
| Variance | 7.3E+05 | | 1.3E+05 | | Variance | | 9.6E+09 | | 2.3E+10 | | |  | | Variance | | 9.6E+09 | | 1.4E+08 | | | |  |
| Observations | 8 | 8 | | Observations | | 8 | | 8 | |  | | | Observations | | | 8 | | | | 8 | | |
| Hypothesized Mean Difference | 0 |  | | Hypothesized Mean Difference | | 0 | |  | |  | | | Hypothesized Mean Difference | | | 0 | | | |  | | |
| df | 9 |  | | df | | 12 | |  | |  | | | Df | | | 7 | | | |  | | |
| t Stat | 0.34 |  | | t Stat | | 0.15 | |  | |  | | | t Stat | | | 3.6631 | | | |  | | |
| P(T<=t) one-tail | 0.37 |  | | P(T<=t) one-tail | | 0.44 | |  | |  | | | P(T<=t) one-tail | | | 0.0040 | | | |  | | |
| t Critical one-tail | 1.83 |  | | t Critical one-tail | | 1.78 | |  | |  | | | t Critical one-tail | | | 1.8946 | | | |  | | |
| P(T<=t) two-tail | 0.74 |  | | P(T<=t) two-tail | | 0.88 | |  | |  | | | P(T<=t) two-tail | | | 0.0080 | | | |  | | |
| t Critical two-tail | 2.26 |  | | t Critical two-tail | | 2.18 | |  | |  | | | t Critical two-tail | | | 2.3646 | | | |  | | |

**D Table: 2-sample T-tests between sample ports:** Experiment 1 Calculations, Part 3

| 2-sample T-tests assuming unequal variances: Testing for significance between sample ports | | | | | | | | |
| --- | --- | --- | --- | --- | --- | --- | --- | --- |
|  | *SP5* | *SP6* |  | *SP1* | *SP4* |  | *SP1* | *SP5* |
| Mean | 1.4E+05 | 2.0E+04 | Mean | 459.4 | 1.5E+05 | Mean | 459.4 | 1.4E+05 |
| Variance | 2.3E+10 | 1.4E+08 | Variance | 7.3E+05 | 9.6E+09 | Variance | 7.3E+05 | 2.3E+10 |
| Observations | 8 | 8 | Observations | 8 | 8 | Observations | 8 | 8 |
| Hypothesized Mean Difference | 0 |  | Hypothesized Mean Difference | 0 |  | Hypothesized Mean Difference | 0 |  |
| df | 7 |  | df | 7 |  | df | 7 |  |
| t Stat | 2.182 |  | t Stat | -4.2597 |  | t Stat | -2.555 |  |
| P(T<=t) one-tail | 0.033 |  | P(T<=t) one-tail | 0.0019 |  | P(T<=t) one-tail | 0.019 |  |
| t Critical one-tail | 1.895 |  | t Critical one-tail | 1.8946 |  | t Critical one-tail | 1.895 |  |
| P(T<=t) two-tail | 0.065 |  | P(T<=t) two-tail | 0.0037 |  | P(T<=t) two-tail | 0.038 |  |
| t Critical two-tail | 2.365 |  | t Critical two-tail | 2.3646 |  | t Critical two-tail | 2.365 |  |

**D Table: 2-sample T-tests between sample ports:** Experiment 1 Calculations, Part 4

| 2-sample T-tests assuming unequal variances: Testing for significance between sample ports | | | | | | | | |
| --- | --- | --- | --- | --- | --- | --- | --- | --- |
|  | *SP1* | *SP6* |  | *SP3* | *SP4* |  | *SP3* | *SP5* |
| Mean | 459.4 | 2.0E+04 | Mean | 346.3 | 1.5E+05 | Mean | 346.3 | 1.4E+05 |
| Variance | 7.3E+05 | 1.4E+08 | Variance | 1.3E+05 | 9.6E+09 | Variance | 1.3E+05 | 2.3E+10 |
| Observations | 8 | 8 | Observations | 8 | 8 | Observations | 8 | 8 |
| Hypothesized Mean Difference | 0 |  | Hypothesized Mean Difference | 0 |  | Hypothesized Mean Difference | 0 |  |
| df | 7 |  | df | 7 |  | df | 7 |  |
| t Stat | -4.6787 |  | t Stat | -4.2631 |  | t Stat | -2.557 |  |
| P(T<=t) one-tail | 0.0011 |  | P(T<=t) one-tail | 0.0019 |  | P(T<=t) one-tail | 0.019 |  |
| t Critical one-tail | 1.8946 |  | t Critical one-tail | 1.8946 |  | t Critical one-tail | 1.895 |  |
| P(T<=t) two-tail | 0.0023 |  | P(T<=t) two-tail | 0.0037 |  | P(T<=t) two-tail | 0.038 |  |
| t Critical two-tail | 2.3646 |  | t Critical two-tail | 2.3646 |  | t Critical two-tail | 2.365 |  |

**D Table: 2-sample T-tests between sample ports:** Experiment 1 Calculations, Part 5

| 2-sample T-tests assuming unequal variances: Testing for significance between sample ports | | |
| --- | --- | --- |
|  | *SP3* | *SP6* |
| Mean | 346.3 | 2.0E+04 |
| Variance | 1.3E+05 | 1.4E+08 |
| Observations | 8 | 8 |
| Hypothesized Mean Difference | 0 |  |
| df | 7 |  |
| t Stat | -4.7155 |  |
| P(T<=t) one-tail | 0.0011 |  |
| t Critical one-tail | 1.8946 |  |
| P(T<=t) two-tail | 0.0022 |  |
| t Critical two-tail | 2.3646 |  |

Sample ports associated with the primary clarifier were SP1 (influent) and SP3 (effluent) were combined for the analysis of data for the “primary clarifier.” SP2 is a port on the apparatus that is not used in this investigation. SP4 and SP5 are the aeration chamber effluent and recycled activated sludge (RAS), respectively and have been combined for this analysis to be associated with the “aeration chamber.” SP6 alone is the secondary clarifier effluent and is the only sample port’s data that is used in the heading “secondary clarifier.

**D Table: 2-sample T-tests between AST components:** Experiment 1 Calculations part 6

| 2-sample T-tests assuming unequal variances: Testing for significance between sample ports | | | | | | | |  |
| --- | --- | --- | --- | --- | --- | --- | --- | --- |
|  | *Primary clarifier* | *Aeration chamber* |  | *Primary clarifier* | *Secondary clarifier* |  | *Aeration chamber* | *Secondary clarifier* |
| Mean | 402.8 | 1.4E+05 | Mean | 402.8 | 2.0E+04 | Mean | 1.4E+05 | 2.0E+04 |
| Variance | 4.1E+05 | 1.5E+10 | Variance | 4.1E+05 | 1.4E+08 | Variance | 1.5E+10 | 1.4E+08 |
| Observations | 16 | 16 | Observations | 16 | 8 | Observations | 16 | 8 |
| Hypothesized Mean Difference | 0 |  | Hypothesized Mean Difference | 0 |  | Hypothesized Mean Difference | 0 |  |
| df | 15 |  | df | 7 |  | df | 16 |  |
| t Stat | -4.61 |  | t Stat | -4.70 |  | t Stat | 3.9320 |  |
| P(T<=t) one-tail | 0.00017 |  | P(T<=t) one-tail | 0.0011 |  | P(T<=t) one-tail | 0.0006 |  |
| t Critical one-tail | 1.75 |  | t Critical one-tail | 1.89 |  | t Critical one-tail | 1.7459 |  |
| P(T<=t) two-tail | 0.00034 |  | P(T<=t) two-tail | 0.0022 |  | P(T<=t) two-tail | 0.0012 |  |
| t Critical two-tail | 2.13145 |  | t Critical two-tail | 2.3646 |  | t Critical two-tail | 2.1199 |  |

**D Table: 2-sample T-tests between AST components:** Experiment 2 Calculations part 7

| t-Test: Two-Sample Assuming Unequal Variances | | | | | | | | |
| --- | --- | --- | --- | --- | --- | --- | --- | --- |
|  | *SP1* | *SP3* |  | *SP4* | *SP5* |  | *SP4* | *SP6* |
| Mean | 916.7 | 1.8E+03 | Mean | 7.3E+04 | 3.0E+04 | Mean | 7.3E+04 | 4.0E+03 |
| Variance | 7.3E+05 | 2.7E+06 | Variance | 7.9E+08 | 1.3E+08 | Variance | 7.9E+08 | 1.6E+06 |
| Observations | 3 | 3 | Observations | 3 | 3 | Observations | 3 | 3 |
| Hypothesized Mean Difference | 0 |  | Hypothesized Mean Difference | 0 |  | Hypothesized Mean Difference | 0 |  |
| df | 3 |  | df | 3 |  | df | 2 |  |
| t Stat | -0.800 |  | t Stat | 2.474 |  | t Stat | 4.250 |  |
| P(T<=t) one-tail | 0.241 |  | P(T<=t) one-tail | 0.045 |  | P(T<=t) one-tail | 0.026 |  |
| t Critical one-tail | 2.353 |  | t Critical one-tail | 2.353 |  | t Critical one-tail | 2.920 |  |
| P(T<=t) two-tail | 0.482 |  | P(T<=t) two-tail | 0.090 |  | P(T<=t) two-tail | 0.051 |  |
| t Critical two-tail | 3.182 |  | t Critical two-tail | 3.182 |  | t Critical two-tail | 4.303 |  |

**D Table: 2-sample T-tests between AST components:** Experiment 2 Calculations part 8

| t-Test: Two-Sample Assuming Unequal Variances | | | | | | | | |
| --- | --- | --- | --- | --- | --- | --- | --- | --- |
|  | *SP5* | *SP6* |  | *SP1* | *SP4* |  | *SP1* | *SP5* |
| Mean | 3.0E+04 | 4.0E+03 | Mean | 916.7 | 7.3E+04 | Mean | 916.7 | 3.0E+04 |
| Variance | 1.3E+08 | 1.6E+06 | Variance | 7.3E+05 | 7.9E+08 | Variance | 7.3E+05 | 1.3E+08 |
| Observations | 3 | 3 | Observations | 3 | 3 | Observations | 3 | 3 |
| Hypothesized Mean Difference | 0 |  | Hypothesized Mean Difference | 0 |  | Hypothesized Mean Difference | 0 |  |
| df | 2 |  | df | 2 |  | df | 2 |  |
| t Stat | 3.856 |  | t Stat | -4.442 |  | t Stat | -4.33 |  |
| P(T<=t) one-tail | 0.031 |  | P(T<=t) one-tail | 0.024 |  | P(T<=t) one-tail | 0.025 |  |
| t Critical one-tail | 2.92 |  | t Critical one-tail | 2.92 |  | t Critical one-tail | 2.92 |  |
| P(T<=t) two-tail | 0.061 |  | P(T<=t) two-tail | 0.047 |  | P(T<=t) two-tail | 0.049 |  |
| t Critical two-tail | 4.303 |  | t Critical two-tail | 4.303 |  | t Critical two-tail | 4.303 |  |

**D Table: 2-sample T-tests between AST components:** Experiment 2 Calculations part 9

| t-Test: Two-Sample Assuming Unequal Variances | | | | | |
| --- | --- | --- | --- | --- | --- |
|  | *SP1* | *SP6* |  | *SP3* | *SP4* |
| Mean | 916.7 | 4.0E+03 | Mean | 1.8E+03 | 7.3E+04 |
| Variance | 7.3E+05 | 1.6E+06 | Variance | 2.7E+06 | 7.9E+08 |
| Observations | 3 | 3 | Observations | 3 | 3 |
| Hypothesized Mean Difference | 0 |  | Hypothesized Mean Difference | 0 |  |
| df | 4 |  | df | 2 |  |
| t Stat | -3.540 |  | t Stat | -4.384 |  |
| P(T<=t) one-tail | 0.012 |  | P(T<=t) one-tail | 0.024 |  |
| t Critical one-tail | 2.132 |  | t Critical one-tail | 2.920 |  |
| P(T<=t) two-tail | 0.024 |  | P(T<=t) two-tail | 0.048 |  |
| t Critical two-tail | 2.776 |  | t Critical two-tail | 4.303 |  |

**D Table: 2-sample T-tests between AST components:** Experiment 2 Calculations part 10

| t-Test: Two-Sample Assuming Unequal Variances | | | | | |
| --- | --- | --- | --- | --- | --- |
|  | *SP3* | *SP5* |  | *SP3* | *SP6* |
| Mean | 1.8E+03 | 3.0E+04 | Mean | 1.8E+03 | 4.0E+03 |
| Variance | 2.7E+06 | 1.3E+08 | Variance | 2.7E+06 | 1.6E+06 |
| Observations | 3 | 3 | Observations | 3 | 3 |
| Hypothesized Mean Difference | 0 |  | Hypothesized Mean Difference | 0 |  |
| df | 2 |  | df | 4 |  |
| t Stat | -4.174 |  | t Stat | -1.887 |  |
| P(T<=t) one-tail | 0.026 |  | P(T<=t) one-tail | 0.066 |  |
| t Critical one-tail | 2.920 |  | t Critical one-tail | 2.132 |  |
| P(T<=t) two-tail | 0.053 |  | P(T<=t) two-tail | 0.132 |  |
| t Critical two-tail | 4.303 |  | t Critical two-tail | 2.776 |  |

**D Table: 2-sample T-tests between AST components:** Experiment 2 Calculations part 11

| t-Test: Two-Sample Assuming Unequal Variances | | | | | | | | |
| --- | --- | --- | --- | --- | --- | --- | --- | --- |
|  | *Primary Clarifier* | *Aeration Basin* |  | *Primary Clarifier* | *Secondary Clarifier* |  | *Aeration Basin* | *Secondary Clarifier* |
| Mean | 1.3E+03 | 5.1E+04 | Mean | 1.3E+03 | 4.0E+03 | Mean | 5.1E+04 | 4.0E+03 |
| Variance | 1.6E+06 | 9.3E+08 | Variance | 1.6E+06 | 1.6E+06 | Variance | 9.3E+08 | 1.6E+06 |
| Observations | 6 | 6 | Observations | 6 | 3 | Observations | 6 | 3 |
| Hypothesized Mean Difference | 0 |  | Hypothesized Mean Difference | 0 |  | Hypothesized Mean Difference | 0 |  |
| df | 5 |  | df | 4 |  | df | 5 |  |
| t Stat | -4.008 |  | t Stat | -3.013 |  | t Stat | 3.792 |  |
| P(T<=t) one-tail | 0.005 |  | P(T<=t) one-tail | 0.020 |  | P(T<=t) one-tail | 0.006 |  |
| t Critical one-tail | 2.015 |  | t Critical one-tail | 2.132 |  | t Critical one-tail | 2.015 |  |
| P(T<=t) two-tail | 0.010 |  | P(T<=t) two-tail | 0.039 |  | P(T<=t) two-tail | 0.013 |  |
| t Critical two-tail | 2.571 |  | t Critical two-tail | 2.776 |  | t Critical two-tail | 2.571 |  |

Finally, the corresponding data collected after the calculated range of the possible SRT from all locations during experiment 1 was combined and compared to the same data collected in the duplicate experiment. In the *B. globigii* experiments, this compared two experiments that were identical in setup, but the second experiment, as can be seen in the “number of observations” field, consisted of fewer data points. The MS2 data was compared using a similar set of analyses. The MS2 experiment’s comparison between experiments 1 and 2, however, was used to compare whether growing the MS2 in the synthetic wastewater or dechlorinated tap water made a significant difference in the persistence of MS2 in the AST system.

**D Table: 2-sample T-tests between *B. globigii* Experiments:** Calculations, Part 12

| 2-sample T-test comparing *B. globigii data between the two experiments* | | |
| --- | --- | --- |
|  | *experiment 1* | *Experiment 2* |
| Mean | 6.1E+04 | 2.2E+04 |
| Variance | 1.1E+10 | 9.6E+08 |
| Observations | 40 | 15 |
| Hypothesized Mean Difference | 0 |  |
| df | 52 |  |
| t Stat | 2.185 |  |
| P(T<=t) one-tail | 0.017 |  |
| t Critical one-tail | 1.675 |  |
| P(T<=t) two-tail | 0.033 |  |
| t Critical two-tail | 2.007 |  |

**E1 Table: Raw data from the first MS2 experiments for Significance Tests.**  This data consists of the data collected at and beyond the SRT’s possible range (t=~28 days). P values used to determine the significance of the data are highlighted. Non-significant results (p > 0.05) are highlighted in red. Significant results (p < 0.05) are highlighted in green. Results that are not statistically significant, but that would be significant if a slightly higher α value (0.05 < p < 0.10) were selected are highlighted in yellow.

| With dechlorinated water | | | | | |  | With SWW | | | | |
| --- | --- | --- | --- | --- | --- | --- | --- | --- | --- | --- | --- |
| time | sp1 | sp3 | sp4 | sp5 | sp6 |  | sp1 | sp3 | sp4 | sp5 | sp6 |
| 28 | 1 | 5 | 1 | 1 | 1 |  | 67 | 4 | 4 | 17 | 3 |
| 35 | 1 | 1 | 1 | 1 | 1 |  | 4 | 1 | 1 | 2 | 1 |
| 42 | 1 | 1 | 1 | 1 | 1 |  | 6 | 1 | 3 | 1 | 1 |
| 49 | 1 | 1 | 1 | 1 | 1 |  | 1 | 1 | 1 | 1 | 1 |

**E Table: Significance test between the sample ports of the MS2 experiment with dechlorinated water.** Part 2. Since most of the recorded values are “1’s”, only one test was needed to test one sample port against the rest of the sample ports. The 1’s represent a reading below the detection limit.

| t-Test: Two-Sample Assuming Unequal Variances | | |
| --- | --- | --- |
|  | *SP1, 4, 5, & 6* | *SP3* |
| Mean | 1 | 2 |
| Variance | 0 | 4 |
| Observations | 4 | 4 |
| Hypothesized Mean Difference | 0 |  |
| df | 3 |  |
| t Stat | -1 |  |
| P(T<=t) one-tail | 0.196 |  |
| t Critical one-tail | 2.353 |  |
| P(T<=t) two-tail | 0.391 |  |
| t Critical two-tail | 3.182 |  |

**E Table: Significance test between the sample ports of the MS2 experiment with synthetic wastewater.**  Part 3

| t-Test: Two-Sample Assuming Unequal Variances | | | t-Test: Two-Sample Assuming Unequal Variances | | | t-Test: Two-Sample Assuming Unequal Variances | | |
| --- | --- | --- | --- | --- | --- | --- | --- | --- |
|  |  |  |  |  |  |  |  |  |
|  | *SP1* | *SP3* |  | *SP4* | *SP5* |  | *SP4* | *SP6* |
| Mean | 19.5 | 1.75 | Mean | 2.25 | 5.25 | Mean | 2.25 | 1.5 |
| Variance | 1007 | 2.25 | Variance | 2.25 | 61.58 | Variance | 2.25 | 1 |
| Observations | 4 | 4 | Observations | 4 | 4 | Observations | 4 | 4 |
| Hypothesized Mean Difference | 0 |  | Hypothesized Mean Difference | 0 |  | Hypothesized Mean Difference | 0 |  |
| df | 3 |  | df | 3 |  | df | 5 |  |
| t Stat | 1.117 |  | t Stat | -0.751 |  | t Stat | 0.832 |  |
| P(T<=t) one-tail | 0.173 |  | P(T<=t) one-tail | 0.254 |  | P(T<=t) one-tail | 0.222 |  |
| t Critical one-tail | 2.353 |  | t Critical one-tail | 2.353 |  | t Critical one-tail | 2.015 |  |
| P(T<=t) two-tail | 0.345 |  | P(T<=t) two-tail | 0.507 |  | P(T<=t) two-tail | 0.443 |  |
| t Critical two-tail | 3.182 |  | t Critical two-tail | 3.182 |  | t Critical two-tail | 2.571 |  |

**E Table: Significance test between the sample ports of the MS2 experiment with synthetic wastewater.**  Part 4

| t-Test: Two-Sample Assuming Unequal Variances | | | t-Test: Two-Sample Assuming Unequal Variances | | | t-Test: Two-Sample Assuming Unequal Variances | | |
| --- | --- | --- | --- | --- | --- | --- | --- | --- |
|  |  |  |  |  |  |  |  |  |
|  | *SP5* | *SP6* |  | *SP1* | *SP4* |  | *SP1* | *SP5* |
| Mean | 5.25 | 1.5 | Mean | 19.5 | 2.25 | Mean | 19.5 | 5.25 |
| Variance | 61.58 | 1 | Variance | 1007 | 2.25 | Variance | 1007 | 61.58 |
| Observations | 4 | 4 | Observations | 4 | 4 | Observations | 4 | 4 |
| Hypothesized Mean Difference | 0 |  | Hypothesized Mean Difference | 0 |  | Hypothesized Mean Difference | 0 |  |
| df | 3 |  | df | 3 |  | df | 3 |  |
| t Stat | 0.948 |  | t Stat | 1.086 |  | t Stat | 0.872 |  |
| P(T<=t) one-tail | 0.207 |  | P(T<=t) one-tail | 0.178 |  | P(T<=t) one-tail | 0.224 |  |
| t Critical one-tail | 2.353 |  | t Critical one-tail | 2.353 |  | t Critical one-tail | 2.353 |  |
| P(T<=t) two-tail | 0.413 |  | P(T<=t) two-tail | 0.357 |  | P(T<=t) two-tail | 0.447 |  |
| t Critical two-tail | 3.182 |  | t Critical two-tail | 3.182 |  | t Critical two-tail | 3.182 |  |

**E Table: Significance test between the sample ports of the MS2 experiment with synthetic wastewater.**  Part 5

| t-Test: Two-Sample Assuming Unequal Variances | | | t-Test: Two-Sample Assuming Unequal Variances | | | t-Test: Two-Sample Assuming Unequal Variances | | |
| --- | --- | --- | --- | --- | --- | --- | --- | --- |
|  |  |  |  |  |  |  |  |  |
|  | *1* | *6* |  | *3* | *4* |  | *3* | *5* |
| Mean | 19.5 | 1.5 | Mean | 1.75 | 2.25 | Mean | 1.75 | 5.25 |
| Variance | 1007 | 1 | Variance | 2.25 | 2.25 | Variance | 2.25 | 61.58 |
| Observations | 4 | 4 | Observations | 4 | 4 | Observations | 4 | 4 |
| Hypothesized Mean Difference | 0 |  | Hypothesized Mean Difference | 0 |  | Hypothesized Mean Difference | 0 |  |
| df | 3 |  | df | 6 |  | df | 3 |  |
| t Stat | 1.134 |  | t Stat | -0.471 |  | t Stat | -0.876 |  |
| P(T<=t) one-tail | 0.170 |  | P(T<=t) one-tail | 0.327 |  | P(T<=t) one-tail | 0.223 |  |
| t Critical one-tail | 2.353 |  | t Critical one-tail | 1.943 |  | t Critical one-tail | 2.353 |  |
| P(T<=t) two-tail | 0.339 |  | P(T<=t) two-tail | 0.654 |  | P(T<=t) two-tail | 0.445 |  |
| t Critical two-tail | 3.182 |  | t Critical two-tail | 2.447 |  | t Critical two-tail | 3.182 |  |

**E Table: Significance test between the AST Components in the MS2 experiment with synthetic wastewater.**  Part 6. Grouping the data according to AST components improves the significance, but it still shows no significant difference in the concentrations between the three compartments of the AST.

| t-Test: Two-Sample Assuming Unequal Variances | | | t-Test: Two-Sample Assuming Unequal Variances | | | t-Test: Two-Sample Assuming Unequal Variances | | |
| --- | --- | --- | --- | --- | --- | --- | --- | --- |
|  |  |  |  |  |  |  |  |  |
|  | *Primary clarifier* | *aeration basin* |  | *Primary clarifier* | *secondary clarifier* |  | *aeration basin* | *secondary clarifier* |
| Mean | 10.62 | 3.75 | Mean | 10.62 | 1.5 | Mean | 3.75 | 1.5 |
| Variance | 522.6 | 29.9 | Variance | 522.6 | 1 | Variance | 29.93 | 1 |
| Observations | 8 | 8 | Observations | 8 | 4 | Observations | 8 | 4 |
| Hypothesized Mean Difference | 0 |  | Hypothesized Mean Difference | 0 |  | Hypothesized Mean Difference | 0 |  |
| df | 8 |  | df | 7 |  | df | 8 |  |
| t Stat | 0.827 |  | t Stat | 1.127 |  | t Stat | 1.126 |  |
| P(T<=t) one-tail | 0.216 |  | P(T<=t) one-tail | 0.148 |  | P(T<=t) one-tail | 0.146 |  |
| t Critical one-tail | 1.860 |  | t Critical one-tail | 1.895 |  | t Critical one-tail | 1.86 |  |
| P(T<=t) two-tail | 0.432 |  | P(T<=t) two-tail | 0.297 |  | P(T<=t) two-tail | 0.293 |  |
| t Critical two-tail | 2.306 |  | t Critical two-tail | 2.365 |  | t Critical two-tail | 2.306 |  |

**E Table: Significance test between the Dechlorinated water and synthetic wastewater experiments with MS2.**  Part 7. Comparing all measurements taken in the dechlorinated water experiment to all measurements taken in synthetic wastewater still showed a p-value indicating no significant difference between the two experiments due to the change in media.

| t-Test: Two-Sample Assuming Unequal Variances | | |
| --- | --- | --- |
|  | *deCl* | *SWW* |
| Mean | 1.2 | 6.05 |
| Variance | 0.8 | 219.1026 |
| Observations | 20 | 20 |
| Hypothesized Mean Difference | 0 |  |
| df | 19 |  |
| t Stat | -1.46 |  |
| P(T<=t) one-tail | 0.080 |  |
| t Critical one-tail | 1.729 |  |
| P(T<=t) two-tail | 0.160 |  |
| t Critical two-tail | 2.093 |  |

**F Table: T-test analysis of variance in *B. globigii* and MS2 experiments after the latest possible SRT:** Summary of D and E Table data analyses.

|  | B. globigii in de-Cl water (Expt 1) | | B. globigii in de-Cl water (Expt 2) | | MS2 in de-Cl water (Expt 1) | | MS2 in SWW (Expt 2) | |
| --- | --- | --- | --- | --- | --- | --- | --- | --- |
| Sample ports compared | P-value | significant difference | P-value | significant difference | P-value | significant difference | P-value | significant difference |
| 1 and 3 | 0.74 | x | 0.48 | x | 0.39 | x | 0.35 | x |
| 4 and 5 | 0.88 | x | 0.090 | x | ^b^N/A | x | 0.51 | x |
| 4 and 6 | 0.0080 | * | 0.051 | x | ^b^N/A | x | 0.44 | x |
| 5 and 6 | 0.065 | x | 0.061 | x | ^b^N/A | x | 0.41 | x |
| 1 and 4 | 0.0037 | ** | 0.047 | * | ^b^N/A | x | 0.36 | x |
| 1 and 5 | 0.038 | * | 0.049 | * | ^b^N/A | x | 0.45 | x |
| 1 and 6 | 0.0023 | ** | 0.024 | * | ^b^N/A | x | 0.34 | x |
| 3 and 4 | 0.0037 | ** | 0.048 | * | 0.39 | x | 0.65 | x |
| 3 and 5 | 0.038 | * | 0.053 | x | 0.39 | x | 0.45 | x |
| 3 and 6 | 0.0022 | ** | 0.132 | x | 0.39 | x | 0.79 | x |
| Primary Clarifier (1 & 3) and Aeration chamber (4 & 5) | 0.00034 | *** | 0.010 | * | 0.35 | x | 0.43 | x |
| Primary clarifier (1 & 3) and Secondary clarifier (6) | 0.0022 | ** | 0.039 | * | 0.35 | x | 0.30 | x |
| Aeration chamber (4 & 5) and Secondary clarifier (6) | 0.0012 | ** | 0.013 | * | 0.37 | x | 0.29 | x |
| ^a^Expt 1 and Expt 2 | 0.033 | * |  |  | 0.16 | x |  |  |

Note: X =p>0.05, * = 0.05>p>0.005, ** = 0.0049>p>0.0005, *** = p<0.00049

^a^ This row compares the first and second experiments using the data collected after 20 days of operation from all sample ports.

^b^ In the MS2 investigation, nearly all MS2 was not detected for most samples. In these instances, a low value of 1 was used as a place holder to aid in graphing the data and using it in statistical analysis. In this case, the data after the SRT for several sample ports were identical values of 1, leading to many similar p-values in the MS2 analysis.

# Statistical comparisons of Surrogates to Total Suspended Solids

**H Table: Average data for *B. globigii* and Total Suspended Solids**

| Sample Time (days) | Avg. Influent BG | Avg. Primary clarifier effluent BG | Avg. Aeration basin effluent BG | Avg. RAS BG | Avg. Effluent BG | Avg. Influent TSS | Avg. Primary clarifier effluent TSS | Avg. Aeration basin effluent TSS | Avg. RAS TSS | Avg. Effluent TSS |
| --- | --- | --- | --- | --- | --- | --- | --- | --- | --- | --- |
| 0 | 223 | 1465 | 1600 | 2650 | 312 | 21 | 30 | 1251 | 822 | 11 |
| 0.18 | 21666667 | 28000000 | 11666667 | 6450000 | 1666667 | 20 | 21 | 1302 | 926 | 11 |
| 1 | 40500000 | 42000000 | 75000000 | 28000000 | 9600000 | 33 | 27 | 1702 | 572 | 5 |
| 2 | 179500000 | 75000000 | 140000000 | 188000000 | 23500000 | 15 | 28 | 1326 | 563 | 146 |
| 3 | 20500000 | 31000000 | 36500000 | 126500000 | 14333333 | 17 | 43 | 652 | 352 | 185 |
| 4 | 3000 | 160000 | 8100000 | 9466667 | 2450000 | 41 | 66 | 659 | 228 | 49 |
| 5 | 2833 | 25000 | 9250000 | 3433333 | 650000 | 24 | 49 | 493 | 208 | 56 |
| 6 | 1450 | 10000 | 6500000 | 3133333 | 650000 | 23 | 22 | 486 | 246 | 18 |
| 7 | 9625 | 80000 | 18700000 | 6000000 | 525000 | 26 | 32 | 859 | 482 | 14 |
| 9 | 1350 | 2233 | 700000 | 193333 | 140000 | 29 | 30 | 428 | 195 | 35 |
| 14 | 330 | 605 | 625000 | 262500 | 99167 | 37 | 30 | 576 | 379 | 85 |
| 16 | 620 | 3175 | 240000 | 140000 | 36000 | 45 | 28 | 538 | 232 | 69 |
| 21 | 2750 | 745 | 1398750 | 134583 | 21250 | 30 | 21 | 461 | 274 | 17 |
| 23 | 145 | 285 | 10000 | 52500 | 11500 | 34 | 26 | 331 | 182 | 42 |
| 28 | 337 | 608 | 807500 | 126750 | 61000 | 14 | 70 | 529 | 260 | 81 |
| 30 | 80 | 105 | 71500 | 45000 | 22000 | 25 | 20 | 301 | 10 | 61 |
| 35 | 1700 | 1081 | 74500 | 60000 | 18467 | 9 | 23 | 270 | 219 | 55 |
| 37 | 480 | 270 | 23000 | 21000 | 10600 | 50 | 22 | 248 | 135 | 95 |
| 42 | 68 | 1835 | 70000 | 17167 | 3758 | 19 | 27 | 289 | 160 | 20 |
| 44 | 110 | 1005 | 125000 | 59500 | 38000 | 22 | 25 | 415 | 186 | 155 |
| 49 | 74 | 1125 | 105175 | 79875 | 19433 | 28 | 16 | 353 | 327 | 175 |
| 51 | 200 | 60 | 225000 | 80000 | 17000 | 76 | 21 | 606 | 354 | 6 |
| 56 | 50 | 50 | 250000 | 270000 | 29000 | 25 | 21 | 517 | 302 | 14 |
| 58 | 150 | 53 | 250000 | 460000 | 21500 | 3 | 2 | 437 | 170 | 6 |

**H Table: Correlation analysis of *B. globigii* and Total Suspended Solids:** Part 2. Correlation analysis in Excel displays R values for comparing each column of data to one another to help identify strong relationships for further investigation using the Excel Regression function.

|  | Sample Time (days) | Avg. Influent BG | Avg. Primary clarifier effluent BG | Avg. Aeration basin effluent BG | Avg. RAS BG | Avg. Effluent BG | Avg. Influent TSS | Avg. Primary clarifier effluent TSS | Avg. Aeration basin effluent TSS | Avg. RAS TSS | Avg. Effluent TSS |
| --- | --- | --- | --- | --- | --- | --- | --- | --- | --- | --- | --- |
| Sample Time (days) | 1.00 |  |  |  |  |  |  |  |  |  |  |
| Avg. Influent BG | -0.32 | 1.00 |  |  |  |  |  |  |  |  |  |
| Avg. Primary clarifier effluent BG | -0.44 | 0.92 | 1.00 |  |  |  |  |  |  |  |  |
| Avg. Aeration basin effluent BG | -0.43 | 0.95 | 0.95 | 1.00 |  |  |  |  |  |  |  |
| Avg. RAS BG | -0.37 | 0.87 | 0.87 | 0.87 | 1.00 |  |  |  |  |  |  |
| Avg. Effluent BG | -0.42 | 0.90 | 0.94 | 0.94 | 0.97 | 1.00 |  |  |  |  |  |
| Avg. Influent TSS | 0.28 | -0.07 | -0.09 | -0.09 | -0.08 | -0.09 | 1.00 |  |  |  |  |
| Avg. Primary clarifier effluent TSS | -0.43 | -0.01 | 0.01 | 0.05 | 0.12 | 0.12 | -0.18 | 1.00 |  |  |  |
| Avg. Aeration basin effluent TSS | -0.60 | 0.56 | 0.72 | 0.67 | 0.43 | 0.56 | -0.15 | 0.07 | 1.00 |  |  |
| Avg. RAS TSS | -0.51 | 0.36 | 0.51 | 0.39 | 0.26 | 0.32 | 0.00 | -0.03 | 0.86 | 1.00 |  |
| Avg. Effluent TSS | 0.03 | 0.31 | 0.29 | 0.27 | 0.52 | 0.44 | 0.44 | 0.18 | -0.17 | -0.16 | 1.00 |

**H Table: Regression correlating *B. globigii* to TSS.** Part 3

| SP1 BG to SP1 TSS | |  |  |  |  |  |  | | |  |
| --- | --- | --- | --- | --- | --- | --- | --- | --- | --- | --- |
|  |  |  |  |  |  |  |  | | |  |
| *Regression Statistics* | |  |  |  |  |  |  | | |  |
| Multiple R | 7.1E-02 |  |  |  |  |  |  | | |  |
| R Square | 5.0E-03 |  |  |  |  |  |  | | |  |
| Adjusted R Square | -4.0E-02 |  |  |  |  |  |  | | |  |
| Standard Error | 3.8E+07 |  |  |  |  |  |  | | |  |
| Observations | 24 |  |  |  |  |  |  | | |  |
|  |  |  |  |  |  |  |  | | |  |
| ANOVA |  |  |  |  |  |  |  | | |  |
|  | *df* | *SS* | *MS* | *F* | *Significance F* |  |  | | |  |
| Regression | 1 | 1.6E+14 | 1.6E+14 | 0.11 | 0.74 |  |  | | |  |
| Residual | 22 | 3.2E+16 | 1.4E+15 |  |  |  |  | | |  |
| Total | 23 | 3.2E+16 |  |  |  |  |  | | |  |
|  |  |  |  |  |  |  |  | | |  |
|  | *Coefficients* | *Standard Error* | *t Stat* | *P-value* | *Lower 95%* | *Upper 95%* | *Lower 95.0%* | *Upper 95.0%* | | |
| Intercept | 1.2E+07 | 8.1E+06 | 1.4E+00 | 1.6E-01 | -5.1E+06 | 2.8E+07 | -5.1E+06 | | 2.8E+07 | |
| X Variable 1 | -7.6E+03 | 2.3E+04 | -3.3E-01 | 7.4E-01 | -5.5E+04 | 4.0E+04 | -5.5E+04 | | 4.0E+04 | |

**H Table: Regression correlating *B. globigii* to TSS.** Part 4

| SP3 BG to TSS | |  |  |  |  |  |  |  |
| --- | --- | --- | --- | --- | --- | --- | --- | --- |
|  |  |  |  |  |  |  |  |  |
| *Regression Statistics* | |  |  |  |  |  |  |  |
| Multiple R | 5.3E-03 |  |  |  |  |  |  |  |
| R Square | 2.8E-05 |  |  |  |  |  |  |  |
| Adjusted R Square | -4.5E-02 |  |  |  |  |  |  |  |
| Standard Error | 1.9E+07 |  |  |  |  |  |  |  |
| Observations | 24 |  |  |  |  |  |  |  |
|  |  |  |  |  |  |  |  |  |
| ANOVA |  |  |  |  |  |  |  |  |
|  | *df* | *SS* | *MS* | *F* | *Significance F* |  |  |  |
| Regression | 1 | 2.2E+11 | 2E+11 | 6.1E-04 | 9.8E-01 |  |  |  |
| Residual | 22 | 7.8E+15 | 4E+14 |  |  |  |  |  |
| Total | 23 | 7.8E+15 |  |  |  |  |  |  |
|  |  |  |  |  |  |  |  |  |
|  | *Coefficients* | *Standard Error* | *t Stat* | *P-value* | *Lower 95%* | *Upper 95%* | *Lower 95.0%* | *Upper 95.0%* |
| Intercept | 7.2E+06 | 8.6E+06 | 8.3E-01 | 4.2E-01 | -1.1E+07 | 2.5E+07 | -1.1E+07 | 2.5E+07 |
| X Variable 1 | 6.5E+03 | 2.6E+05 | 2.5E-02 | 9.8E-01 | -5.4E+05 | 5.6E+05 | -5.4E+05 | 5.6E+05 |

**H Table: Regression correlating *B. globigii* to TSS.** Part 5

| SP4 BG to TSS | |  |  |  |  |  |  |  |
| --- | --- | --- | --- | --- | --- | --- | --- | --- |
|  |  |  |  |  |  |  |  |  |
| *Regression Statistics* | |  |  |  |  |  |  |  |
| Multiple R | 0.67 |  |  |  |  |  |  |  |
| R Square | 0.45 |  |  |  |  |  |  |  |
| Adjusted R Square | 0.43 |  |  |  |  |  |  |  |
| Standard Error | 2.4E+07 |  |  |  |  |  |  |  |
| Observations | 24 |  |  |  |  |  |  |  |
|  |  |  |  |  |  |  |  |  |
| ANOVA |  |  |  |  |  |  |  |  |
|  | *df* | *SS* | *MS* | *F* | *Significance F* |  |  |  |
| Regression | 1 | 1.0E+16 | 1.0E+16 | 18.1 | 3.2E-04 |  |  |  |
| Residual | 22 | 1.3E+16 | 5.8E+14 |  |  |  |  |  |
| Total | 23 | 2.3E+16 |  |  |  |  |  |  |
|  |  |  |  |  |  |  |  |  |
|  | *Coefficients* | *Standard Error* | *t Stat* | *P-value* | *Lower 95%* | *Upper 95%* | *Lower 95.0%* | *Upper 95.0%* |
| Intercept | -2.2E+07 | 9.5E+06 | -2.3E+00 | 3.2E-02 | -4.1E+07 | -2.0E+06 | -4.1E+07 | -2.0E+06 |
| X Variable 1 | 5.5E+04 | 1.3E+04 | 4.3E+00 | 3.2E-04 | 2.8E+04 | 8.2E+04 | 2.8E+04 | 8.2E+04 |

**H Table: Regression correlating *B. globigii* to TSS.** Part 6

| SP5 BG to TSS | |  |  |  |  |  |  |  |
| --- | --- | --- | --- | --- | --- | --- | --- | --- |
|  |  |  |  |  |  |  |  |  |
| *Regression Statistics* | |  |  |  |  |  |  |  |
| Multiple R | 0.26 |  |  |  |  |  |  |  |
| R Square | 0.07 |  |  |  |  |  |  |  |
| Adjusted R Square | 0.03 |  |  |  |  |  |  |  |
| Standard Error | 4.4E+07 |  |  |  |  |  |  |  |
| Observations | 24 |  |  |  |  |  |  |  |
|  |  |  |  |  |  |  |  |  |
| ANOVA |  |  |  |  |  |  |  |  |
|  | *df* | *SS* | *MS* | *F* | *Significance F* |  |  |  |
| Regression | 1 | 3.2E+15 | 3.2E+15 | 1.64 | 0.21 |  |  |  |
| Residual | 22 | 4.3E+16 | 2.0E+15 |  |  |  |  |  |
| Total | 23 | 4.7E+16 |  |  |  |  |  |  |
|  |  |  |  |  |  |  |  |  |
|  | *Coefficients* | *Standard Error* | *t Stat* | *P-value* | *Lower 95%* | *Upper 95%* | *Lower 95.0%* | *Upper 95.0%* |
| Intercept | -2.4E+06 | 1.7E+07 | -1.5E-01 | 8.9E-01 | -3.7E+07 | 3.2E+07 | -3.7E+07 | 3.2E+07 |
| X Variable 1 | 5.5E+04 | 4.3E+04 | 1.3E+00 | 2.1E-01 | -3.4E+04 | 1.4E+05 | -3.4E+04 | 1.4E+05 |

**H Table: Regression correlating *B. globigii* to TSS.** Part 7

| SP6 BG to TSS | |  |  |  |  |  |  |  |
| --- | --- | --- | --- | --- | --- | --- | --- | --- |
|  |  |  |  |  |  |  |  |  |
| *Regression Statistics* | |  |  |  |  |  |  |  |
| Multiple R | 0.44 |  |  |  |  |  |  |  |
| R Square | 0.19 |  |  |  |  |  |  |  |
| Adjusted R Square | 0.16 |  |  |  |  |  |  |  |
| Standard Error | 5.2E+06 |  |  |  |  |  |  |  |
| Observations | 24 |  |  |  |  |  |  |  |
|  |  |  |  |  |  |  |  |  |
| ANOVA |  |  |  |  |  |  |  |  |
|  | *df* | *SS* | *MS* | *F* | *Significance F* |  |  |  |
| Regression | 1 | 1.4E+14 | 1.4E+14 | 5.23 | 0.032 |  |  |  |
| Residual | 22 | 6.0E+14 | 2.7E+13 |  |  |  |  |  |
| Total | 23 | 7.4E+14 |  |  |  |  |  |  |
|  |  |  |  |  |  |  |  |  |
|  | *Coefficients* | *Standard Error* | *t Stat* | *P-value* | *Lower 95%* | *Upper 95%* | *Lower 95.0%* | *Upper 95.0%* |
| Intercept | -3.7E+05 | 1.6E+06 | -2.4E-01 | 8.2E-01 | -3.6E+06 | 2.9E+06 | -3.6E+06 | 2.9E+06 |
| X Variable 1 | 4.5E+04 | 1.9E+04 | 2.3E+00 | 3.2E-02 | 4.2E+03 | 8.5E+04 | 4.2E+03 | 8.5E+04 |

**H Table: Average data for MS2 and Total Suspended Solids.** Part 8

| Sample Time (days) | Average Influent MS2 | Average Primary clarifier MS2 | Average Aeration basin effluent MS2 | Average Return activated sludge MS2 | Average Effluent MS2 | Average Influent TSS | Average primary clarifier effluent TSS | Average Aeration basin effluent TSS | Average return activated sludge TSS | Average Effluent TSS |
| --- | --- | --- | --- | --- | --- | --- | --- | --- | --- | --- |
| 0 | 1.0E+00 | 1.0E+00 | 1.0E+00 | 1.0E+00 | 1.0E+00 | 8.7E+00 | 9.0E+00 | 9.5E+02 | 6.5E+02 | 5.1E+00 |
| 0.17 | 4.9E+06 | 3.5E+06 | 1.1E+06 | 1.4E+06 | 1.7E+06 | 2.5E+01 | 1.4E+01 | 1.2E+03 | 8.0E+02 | 4.2E+00 |
| 1.00 | 6.5E+05 | 6.7E+05 | 2.7E+05 | 3.6E+05 | 1.3E+06 | 5.1E+01 | 1.9E+01 | 5.3E+02 | 5.5E+02 | 7.1E+00 |
| 2.00 | 2.8E+05 | 3.8E+05 | 2.8E+05 | 2.6E+05 | 9.6E+05 | 6.0E+02 | 2.8E+01 | 6.4E+02 | 4.9E+02 | 3.2E+00 |
| 3.00 | 5.5E+05 | 5.5E+05 | 5.5E+05 | 6.5E+05 | 6.8E+05 | 3.9E+01 | 2.1E+01 | 7.4E+02 | 4.6E+02 | 5.0E+01 |
| 7.00 | 7.5E+02 | 4.5E+02 | 1.2E+03 | 8.5E+02 | 8.4E+02 | 1.6E+02 | 2.2E+01 | 1.7E+03 | 5.8E+02 | 3.3E+01 |
| 14.00 | 7.0E+00 | 2.9E+01 | 3.6E+01 | 1.5E+01 | 4.9E+01 | 8.8E+02 | 2.3E+01 | 1.1E+03 | 5.7E+02 | 4.7E+01 |
| 21.00 | 8.3E+00 | 2.1E+01 | 2.1E+01 | 1.8E+01 | 1.4E+01 | 3.4E+02 | 2.2E+01 | 1.1E+03 | 3.7E+02 | 2.1E+01 |
| 28.00 | 3.4E+01 | 4.5E+00 | 2.5E+00 | 9.0E+00 | 2.0E+00 | 2.5E+03 | 3.6E+01 | 2.7E+03 | 5.7E+02 | 4.5E+01 |
| 35.00 | 2.5E+00 | 1.0E+00 | 1.0E+00 | 1.5E+00 | 1.0E+00 | 1.1E+03 | 3.1E+01 | 1.2E+03 | 4.9E+02 | 2.9E+01 |
| 42.00 | 3.5E+00 | 1.0E+00 | 2.0E+00 | 1.0E+00 | 1.0E+00 | 4.2E+02 | 5.9E+01 | 9.5E+02 | 4.3E+02 | 7.6E+01 |
| 49.00 | 1.0E+00 | 1.0E+00 | 1.0E+00 | 1.0E+00 | 1.0E+00 | 1.7E+02 | 3.5E+01 | 5.6E+02 | 2.1E+02 | 1.1E+01 |

**H Table: Correlation analysis of MS2 and Total Suspended Solids:** Part 9. Correlation analysis in Excel displays R values for comparing each column of data to one another to help identify strong relationships for further investigation using the Excel Regression function.

|  | Sample Time (days) | Average Influent MS2 | Average Primary clarifier MS2 | Average Aeration basin effluent MS2 | Average Return activated sludge MS2 | Average Effluent MS2 | Average Influent TSS | Average primary clarifier effluent TSS | Average Aeration basin effluent TSS | Average return activated sludge TSS | Average Effluent TSS |
| --- | --- | --- | --- | --- | --- | --- | --- | --- | --- | --- | --- |
| Sample Time (days) | 1.00 |  |  |  |  |  |  |  |  |  |  |
| Average Influent MS2 | -0.38 | 1.00 |  |  |  |  |  |  |  |  |  |
| Average Primary clarifier MS2 | -0.42 | 1.00 | 1.00 |  |  |  |  |  |  |  |  |
| Average Aeration basin effluent MS2 | -0.52 | 0.92 | 0.94 | 1.00 |  |  |  |  |  |  |  |
| Average Return activated sludge MS2 | -0.51 | 0.93 | 0.95 | 1.00 | 1.00 |  |  |  |  |  |  |
| Average Effluent MS2 | -0.61 | 0.78 | 0.83 | 0.88 | 0.87 | 1.00 |  |  |  |  |  |
| Average Influent TSS | 0.36 | -0.27 | -0.30 | -0.35 | -0.35 | -0.36 | 1.00 |  |  |  |  |
| Average primary clarifier effluent TSS | 0.80 | -0.34 | -0.36 | -0.38 | -0.39 | -0.39 | 0.36 | 1.00 |  |  |  |
| Average Aeration basin effluent TSS | 0.11 | -0.02 | -0.06 | -0.17 | -0.15 | -0.32 | 0.76 | 0.11 | 1.00 |  |  |
| Average return activated sludge TSS | -0.68 | 0.62 | 0.61 | 0.53 | 0.54 | 0.47 | 0.03 | -0.52 | 0.36 | 1.00 |  |
| Average Effluent TSS | 0.42 | -0.34 | -0.36 | -0.30 | -0.29 | -0.47 | 0.34 | 0.67 | 0.32 | -0.17 | 1.00 |

Attempts to correlate Surrogate concentrations at each sample port to its corresponding TSS data to determine statistically whether surrogate concentrations associated with the suspended solids were inconclusive.

# Observations Regarding Aerobic Organisms During Surrogate Persistence Studies

A detailed characterization of the entire microbial population in the AST system is a daunting task and was not in the scope of this study. However, a wealth of literature exists on microbial communities and their structural changes in AST systems. For the present study, the activated sludge system density appeared to decrease within two weeks after the introduction of MS2 compared to *B. globigii*, based on visual observation (H and I Figs.). Even though the supply of COD was the same, the formation of filamentous organisms during the MS2 test, as indicated by white foam, may be a possible reason for the observed lower density (I Fig.). Such a system change was not observed during the Phi-6 tests (J Fig.) probably because (a) a low spiking volume of Phi-6 was employed for a shorter duration (1 day), and (b) the AST system was not operated as long because of the shorter observed persistence of Phi-6.


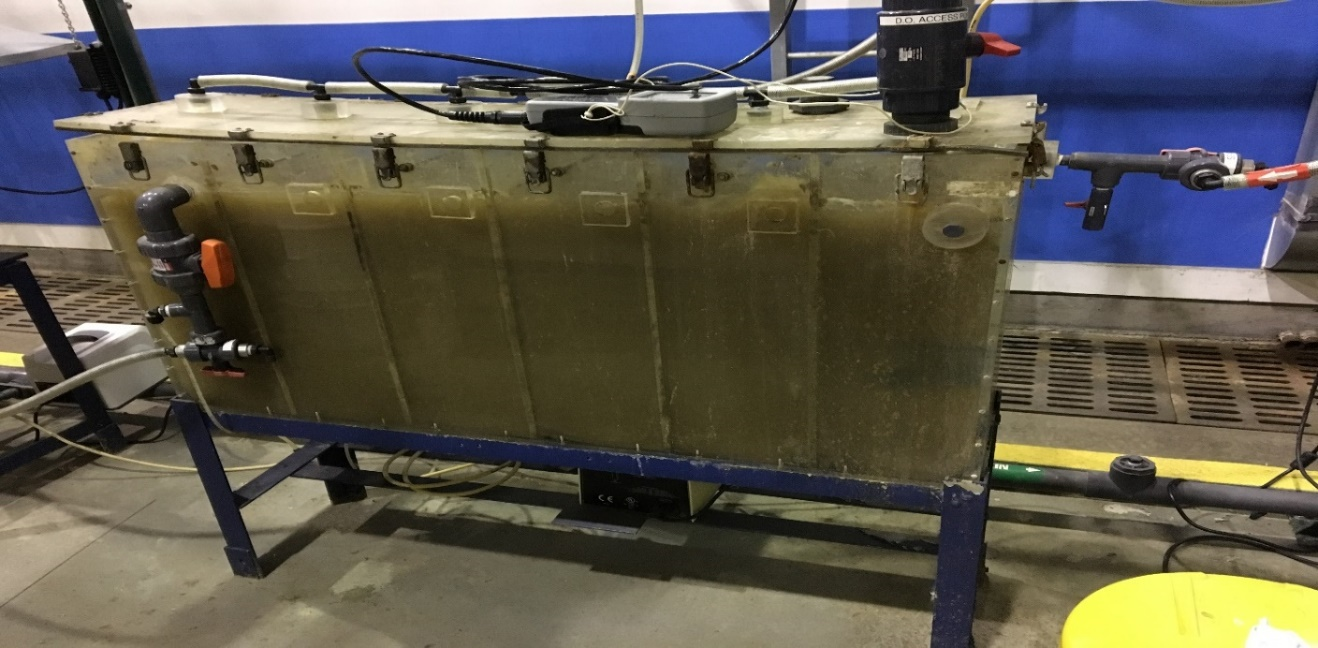


**H Fig.** **Photograph of the aeration tank of the pilot-scale activated sludge system** ~two weeks after injection of *Bacillus globigii*.


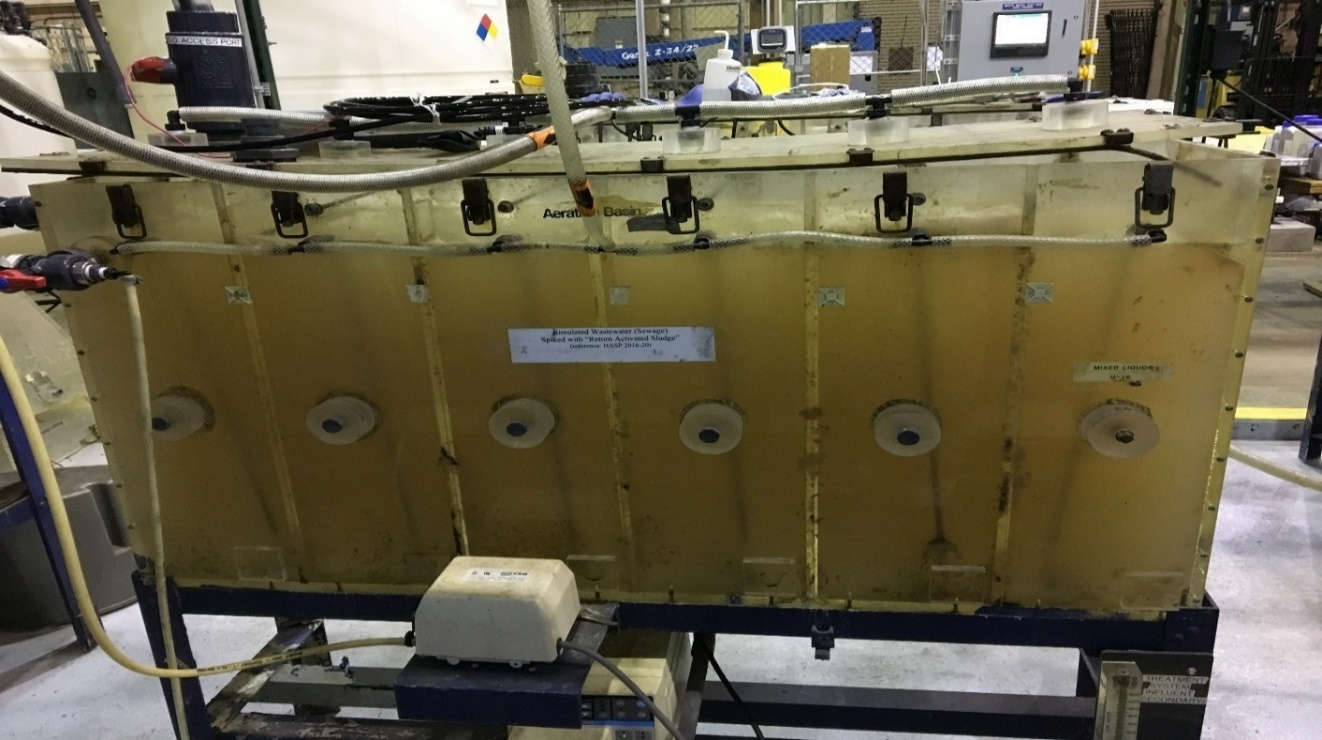


**I Fig. Photograph of the aeration tank of the pilot-scale activated sludge system** ~two weeks after injection of MS2.


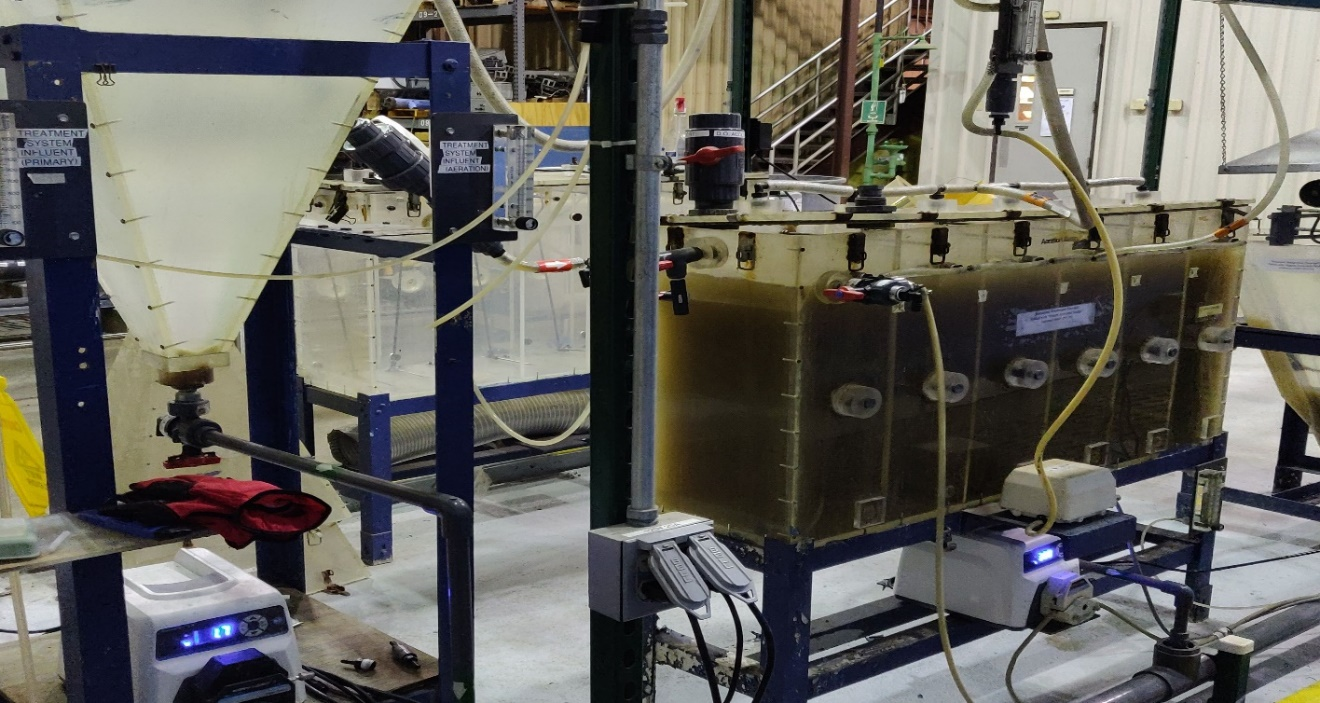


**J Fig.** **Photograph of the aeration tank of the pilot-scale activated sludge system** ~one week after injection of Phi-6.

**References**

1. Q. Zhang, M. T. Suidan, K. Zhang, D. B. Oerther, and A. D. Venosa, Comparison of Biomass Selection between a Novel Membrane Bioreactor and Activated Sludge Process, WEFTEC 06 proceedings, 2006.
2. M. L. Barrios-Hernandez, M. Pronk, H. Garcia, A. Boersma, D. Brdjanovic, M. C. M. van Loosdrecht. and C. M. Hooijmans, Removal of bacterial and viral indicator organisms in full-scale aerobic granular sludge and conventional activated sludge systems, *Water Research*, 2020, **6**, 100040.
3. M. Clara, N. Krauzinger, B. Strenn, O. Gans, H. Kroiss, The solids retention time—a suitable design parameter to evaluate the capacity of wastewater treatment plants to remove micropollutants, *Water Research*, 2005, **39**, 97-106.
4. C. Muriuki, P. Kairigo, P. Home, E. Ngumba, J. Raude, A. Gachanja, and T. Tuhkanen, Mass loading, distribution, and removal of antibiotics and antiretroviral drugs in selected wastewater treatment plants in Kenya, *Science of the Total Environment*, 2020, **743**, 13 p.
5. G. Tchobanoglous and F. L. Burton, Wastewater Engineering: Treatment, Disposal, and Reuse. Third Edition, Metcalf & Eddy Inc. Irwin/McGraw-Hill, 1991, ISBN 0-07-041690-7.
6. J. Brady, Activated Sludge. In Operation of Wastewater Treatment Plants: A Field Study Training Program. Prepared by California State University for USEPA, Sacramento, CA. 2nd Edn., 1980, Vol. 2.
7. L. Grady Jr., G. T. Daigger, and H. C. Lim, Biological Wastewater: Second Edition, Revised and Expanded, Marcel Dekker, Inc. New York, 1999, 1093p. ISBN: 0-8247-8919-9.
