# Supplementary material for: Persistence of surrogates for high consequence viral and bacterial pathogens in a pilot-scale activated sludge treatment system
Source: PLoS One. 2022 Oct 7;17(10):e0275482. doi: 10.1371/journal.pone.0275482 (PMC9543761; doi:10.1371/journal.pone.0275482)
Supplement: S2 Text — (DOCX) [file pone.0275482.s002.docx]

# S2 Text: Compilation of Original Data

**Persistence of Surrogates for High Consequence Viral and Bacterial Pathogens in a Pilot-Scale Activated Sludge Treatment System**

Donald A. Schupp^,1,‡^, Adam C. Burdsall*^2^, Rendahandi G. Silva^1,‡^, J. Lee Heckman^1^, E. Radha Krishnan^1^, Jeffrey G. Szabo^2^, and Matthew Magnuson^2^

1. APTIM Federal Services, 1600 Gest St., Cincinnati, OH 45204

2. U.S. Environmental Protection Agency, Office of Research and Development, Center for Environmental Solutions and Emergency Response, Homeland Security and Materials Management Division, 26 W. Martin Luther King Dr., Cincinnati, OH 45268

*Corresponding Author

‡Contributed equally

# Compilation of raw data from this research

Experiment 1 data with B. globigii: Note that concentrations expressed here are raw from the data collection (CFU/mL) and was converted to CFU/100 mL during analysis to compare to the bacteriophage results.

| *B. globigii* (CFU/mL) Experiment 1 | | | | | | | | | | | | | | | | | | | | | |
| --- | --- | --- | --- | --- | --- | --- | --- | --- | --- | --- | --- | --- | --- | --- | --- | --- | --- | --- | --- | --- | --- |
|  |  |  | SP-01 | | | | SP-03 | | | SP-04 | | | SP-05 | | | | SP-06 | | | |  |
| Stage | Sampling Date | Sample Time (days) | Influent | | | | Primary Clarifier Effluent | | | Aeration Basin Effluent | | | Return Activated Sludge | | | | Effluent | | | |  |
| Pre-Contam-ination | 9/13/16 | 0.0 | 3 | 4.1 |  | 4.5 | | 2.1 |  | 7 |  |  | 45 | 31 |  | 10 | | 5 | 2.5 |  |  |
| During Contam-ination | 9/13/16 | 0.1 | 1.3E+06 | 6.4E+05 |  | 9.0E+05 | | 4.9E+05 |  | 4.2E+04 |  |  | 4.5E+04 | 4.5E+04 |  | 6.0E+03 | | 5.3E+03 |  |  |  |
|  | 9/13/16 | 0.2 | 4.5E+05 | 4.6E+05 |  | 4.5E+05 | | 4.7E+05 |  | 2.3E+05 |  |  | 3.2E+05 |  |  | 8.1E+04 | |  |  |  |  |
|  | 9/13/16 | 0.3 | 4.0E+05 | 5.8E+05 |  | 7.0E+05 | | 5.8E+05 |  | 5.4E+05 |  |  | 3.1E+05 |  |  | 1.4E+05 | | 9.4E+04 |  |  |  |
|  | 9/14/16 | 1.0 | 3.0E+05 | 5.1E+05 |  | 4.0E+05 | | 4.4E+05 |  | 7.5E+05 |  |  | 2.8E+05 |  |  | 9.6E+04 | |  |  |  |  |
|  | 9/14/16 | 1.3 | 2.9E+06 |  |  | 2.3E+06 | | 6.3E+05 |  | 2.2E+06 |  |  | 1.5E+06 |  |  | 1.7E+05 | |  |  |  |  |
|  | 9/15/16 | 2.0 | 3.0E+06 | 5.9E+05 |  | 1.0E+06 | | 5.0E+05 |  | 1.4E+06 |  |  | 2.8E+06 | 9.6E+05 |  | 2.0E+05 | | 2.7E+05 |  |  |  |
|  | 9/16/16 | 3.0 | 2.5E+05 | 1.6E+05 |  | 4.0E+05 | | 2.2E+05 |  | 3.5E+05 | 3.8E+05 |  | 1.9E+06 | 6.3E+05 |  | 1.0E+05 | | 1.7E+05 | 1.6E+05 |  |  |
|  | 9/16/16 | 3.1 | 1.0E+04 | 5.5E+03 | 7.3E+03 | 1.1E+05 | | 1.0E+05 |  | 1.5E+05 | 3.4E+05 |  | 3.0E+05 | 5.3E+05 |  | 1.5E+05 | | 1.9E+05 | 1.8E+05 |  |  |
|  | 9/16/16 | 3.2 | 1.0E+04 | 6.5E+03 | 4.2E+03 | 5.5E+03 | | 5.5E+03 |  | 5.0E+04 | 4.0E+05 | 2.1E+05 | 1.0E+05 | 3.1E+05 |  | 2.0E+05 | | 1.5E+05 | 1.3E+05 |  |  |
|  | 9/16/16 | 3.3 | 200 |  |  | 3.5E+03 | | 3.8E+03 |  | 2.0E+05 | 4.0E+05 |  | 2.5E+05 | 2.0E+05 |  | 1.5E+05 | | 1.7E+05 | 1.2E+05 |  |  |
| Post-Contam-ination | 9/17/16 | 4.0 | 25 | 35 |  | 1.5E+03 | | 1.7E+03 |  | 1.0E+05 | 5.0E+04 | 9.3E+04 | 1.0E+05 | 1.0E+05 | 8.4E+04 | 1.5E+04 | | 3.4E+04 |  |  |  |
|  | 9/18/16 | 5.0 | 50 | 15 | 20 | 250 | |  |  | 4.5E+04 | 1.4E+05 |  | 5.0E+04 | 2.5E+04 | 2.8E+04 | 6.5E+03 | |  |  |  |  |
|  | 9/19/16 | 6.0 | 15 | 14 |  | 100 | |  |  | 1.0E+05 | 6.0E+04 | 3.5E+04 | 5.0E+04 | 1.5E+04 | 2.9E+04 | 6.5E+03 | |  |  |  |  |
|  | 9/20/16 | 7.0 | 5 | 20 |  | 200 | | 300 |  | 1.0E+05 | 1.0E+04 | 4.2E+04 | 5.0E+04 | 1.0E+04 | 7.5E+04 | 5.5E+03 | |  |  |  |  |
|  | 9/22/16 | 9.0 | 11 | 16 |  | 25 | | 23 | 19 | 7.0E+03 | 7.0E+03 |  | 1.5E+03 | 2.6E+03 | 1.7E+03 | 2.0E+03 | | 1.1E+03 | 1.1E+03 |  |  |
|  | 9/27/16 | 14.0 | 3.5 | 2.7 |  | 6.5 | | 4.2 |  | 1.5E+03 |  |  | 1.1E+03 |  |  | 990 | |  |  |  |  |
|  | 9/29/16 | 16.0 | 6.4 | 6 |  | 33.5 | | 30 |  | 2.4E+03 |  |  | 1.4E+03 |  |  | 420 | | 300 |  |  |  |
|  | 10/4/16 | 21.0 | 50 |  |  | 11 | | 0.8 |  | 750 | 1.2E+03 |  | 550 | 1100 |  | 220 | |  |  |  |  |
|  | 10/6/16 | 23.0 | 1.4 | 1.5 |  | 3.2 | | 2.5 |  | 100 | 100 |  | 500 | 550 |  | 130 | | 100 |  |  |  |
|  | 10/11/16 | 28.0 | 2 | 1.5 |  | 8 | | 6.3 |  | 6.0E+03 | 2.3E+03 |  | 3.1E+03 | 1.6E+03 |  | 1.3E+03 | | 990 |  |  |  |
|  | 10/13/16 | 30.0 | 1 | 0.6 |  | 1.5 | | 0.6 |  | 600 | 830 |  | 450 | 450 |  | 200 | | 240 |  |  |  |
|  | 10/17/16 | 34.0 | 25.5 |  |  | 15 | | 0.25 |  | 600 | 910 |  | 950 | 650 |  | 300 | | 330 |  |  |  |
|  | 10/20/16 | 37.0 | 7.5 | 2.1 |  | 4 | | 1.4 |  | 200 | 260 |  | 200 | 240 | 190 | 100 | | 120 | 98 |  |  |
|  | 10/25/16 | 42.0 | 0.5 | 0.2 |  | 1.5 | | 0.9 |  | 200 | 300 |  | 150 | 210 | 150 | 50 | | 30 | 51 |  |  |
|  | 10/27/16 | 44.0 | 1 | 1.2 |  | 10.5 | | 9.6 |  | 1.1E+03 | 1.4E+03 |  | 500 | 690 |  | 400 | | 360 |  |  |  |
|  | 11/1/16 | 49.0 | 1 |  |  | 4.5 | |  |  | 2.1E+03 |  |  | 1.2E+03 | 1.1E+03 |  | 100 | | 110 | 76 |  |  |
|  | 11/3/16 | 51.0 | 2 |  |  | 0.5 | | 0.7 |  | 2.4E+03 | 2.1E+03 |  | 600 | 1.0E+03 |  | 200 | | 180 | 130 |  |  |
|  | 11/8/16 | 56.0 | 0.5 |  |  | 0.5 | |  |  | 2.6E+03 | 2.4E+03 |  | 3.2E+03 | 2.2E+03 |  | 300 | | 320 | 250 |  |  |
|  | 11/10/16 | 58.0 | 1.5 |  |  | 0.05 | | 1 |  | 2.5E+03 |  |  | 4.6E+03 |  |  | 280 | | 150 |  |  |  |

| TSS (mg/L) in *B. globigii* Experiment 1 | | | | | | | |
| --- | --- | --- | --- | --- | --- | --- | --- |
|  |  |  | SP-01 | SP-03 | SP-04 | SP-05 | SP-06 |
| Stage | Sampling Date | Sample Time (days) | Influent | Primary Clarifier Effluent | Aeration Basin Effluent | Return Activated Sludge | Effluent |
| Pre-contamination | 9/13/2016 | 0.0 | 27 | 24 | 1548 | 600 | 16 |
| During contamination | 9/14/2016 | 1.0 | 33 | 27 | 1702 | 572 | 5 |
|  | 9/15/2016 | 2.0 | 15 | 28 | 1326 | 563 | 146 |
|  | 9/16/2016 | 3.0 | 17 | 43 | 652 | 352 | 185 |
| Post-Contamination | 9/17/2016 | 4.0 | 41 | 66 | 659 | 228 | 49 |
|  | 9/18/2016 | 5.0 | 24 | 49 | 493 | 208 | 56 |
|  | 9/19/2016 | 6.0 | 23 | 22 | 486 | 246 | 18 |
|  | 9/20/2016 | 7.0 | 25 | 32 | 647 | 222 | 22 |
|  | 9/22/2016 | 9.0 | 29 | 30 | 428 | 195 | 35 |
|  | 9/27/2016 | 14.0 | 37 | 28 | 789 | 298 | 164 |
|  | 9/29/2016 | 16.0 | 45 | 28 | 538 | 232 | 69 |
|  | 10/4/2016 | 21.0 | 52 | 36 | 378 | 234 | 33 |
|  | 10/6/2016 | 23.0 | 34 | 26 | 331 | 182 | 42 |
|  | 10/11/2016 | 28.0 | 19 | 25 | 570 | 277 | 134 |
|  | 10/13/2016 | 30.0 | 25 | 20 | 301 | 10 | 61 |
|  | 10/17/2016 | 34.0 | 8 | 30 | 258 | 150 | 87 |
|  | 10/20/2016 | 37.0 | 50 | 22 | 248 | 135 | 95 |
|  | 10/25/2016 | 42.0 | 36 | 28 | 289 | 109 | 6 |
|  | 10/27/2016 | 44.0 | 22 | 25 | 415 | 186 | 155 |
|  | 11/1/2016 | 49.0 | 21 | 19 | 680 | 333 | 9 |
|  | 11/3/2016 | 51.0 | 76 | 21 | 606 | 354 | 6 |
|  | 11/8/2016 | 56.0 | 25 | 21 | 517 | 302 | 14 |
|  | 11/10/2016 | 58.0 | 3 | 2 | 437 | 170 | 6 |

| COD (mg/L) in *B. globigii* experiment 1 | | | | | | | | |
| --- | --- | --- | --- | --- | --- | --- | --- | --- |
|  |  |  | SP-01 | SP-03 | SP-04 | SP-05 | SP-06 |  |
| Stage | Sampling Date | Sample Time (days) | Influent | Primary Clarifier Effluent | Aeration Basin Effluent | Return Activated Sludge | Effluent | COD Removal (%) |
| Pre-contamination | 9/13/2016 | 0.0 | 218 | 211 | 84 | 79 | 68 | 68.8 |
| During Contamination | 9/13/2016 | 0.1 | 234 | 218 | 192 | 68 | 56 | 76.1 |
|  | 9/13/2016 | 0.2 | 226 | 218 | 211 | 84 | 79 | 65.0 |
|  | 9/13/2016 | 0.3 | 197 | 198 | 122 | 96 | 63 | 68.0 |
|  | 9/14/2016 | 1.0 | 226 | 210 | 57 | 48 | 45 | 80.1 |
|  | 9/14/2016 | 1.3 | 244 | 257 | 90 | 72 | 68 | 72.1 |
|  | 9/15/2016 | 2.0 | 229 | 192 | 47 | 50 | 52 | 77.3 |
|  | 9/16/2016 | 3.0 | 259 | 281 | 79 | 80 | 73 | 71.8 |
|  | 9/16/2016 | 3.1 | 235 | 258 | 75 | 66 | 68 | 71.1 |
|  | 9/16/2016 | 3.2 | 238 | 240 | 69 |  |  |  |
|  | 9/16/2016 | 3.3 | 206 | 187 | 80 | 74 | 102 | 50.5 |
| Post-Contamination | 9/17/2016 | 4.0 | 199 | 194 | 61 | 55 | 54 | 72.9 |
|  | 9/18/2016 | 5.0 | 235 | 207 | 70 | 73 | 70 | 70.2 |
|  | 9/19/2016 | 6.0 | 219 | 224 | 88 | 91 | 92 | 58.0 |
|  | 9/20/2016 | 7.0 | 233 | 234 | 93 | 81 | 89 | 61.8 |
|  | 9/22/2016 | 9.0 | 213 | 217 | 60 | 73 | 75 | 64.8 |
|  | 9/27/2016 | 14.0 | 252 | 227 | 84 | 76 | 74 | 70.6 |
|  | 9/29/2016 | 16.0 | 230 | 238 | 93 | 87 | 89 | 61.3 |
|  | 10/4/2016 | 21.0 | 238 | 254 | 85 | 81 | 81 | 66.0 |
|  | 10/6/2016 | 23.0 | 230 | 240 | 75 | 81 | 74 | 67.8 |
|  | 10/11/2016 | 28.0 | 247 | 267 | 100 | 78 | 63 | 74.5 |
|  | 10/13/2016 | 30.0 | 244 | 183 | 74 | 71 | 73 | 70.1 |
|  | 10/17/2016 | 34.0 | 213 | 242 | 76 | 78 | 69 | 67.6 |
|  | 10/20/2016 | 37.0 | 218 | 231 | 54 | 76 | 61 | 72.0 |
|  | 10/25/2016 | 42.0 | 199 | 231 | 44 | 71 | 56 | 71.9 |
|  | 10/27/2016 | 44.0 | 246 | 244 | 98 | 75 | 85 | 65.4 |
|  | 11/1/2016 | 49.0 | 237 | 260 | 77 | 78 | 77 | 67.5 |
|  | 11/3/2016 | 51.0 | 248 | 242 | 72 | 73 | 80 | 67.7 |
|  | 11/8/2016 | 56.0 | 174 | 232 | 51 | 44 | 76 | 56.3 |
|  | 11/10/2016 | 58.0 | 197 | 228 | 63 | 74 | 53 | 73.1 |

| NH3 (mg/L) in *B. globigii* experiment 1 | | | | | | | | |
| --- | --- | --- | --- | --- | --- | --- | --- | --- |
|  |  |  | SP-01 | SP-03 | SP-04 | SP-05 | SP-06 |  |
| Stage | Sampling Date | Sample Time (days) | Influent | Primary Clarifier Effluent | Aeration Basin Effluent | Return Activated Sludge | Effluent | NH3 Removal (%) |
| Pre-contamination | 9/13/2016 | 0.0 | 20.4 | 22.3 | 10.1 | 9.22 | 9.33 | 54.3 |
| During Contamination | 9/13/2016 | 0.1 | 21.9 | 22.9 | 9.88 | 9.01 | 9.29 | 57.6 |
|  | 9/13/2016 | 0.2 | 18 | 21.9 | 10.1 | 9.86 | 9.75 | 45.8 |
|  | 9/13/2016 | 0.3 | 21 | 22.1 | 11.6 | 9.98 | 9.95 | 52.6 |
|  | 9/14/2016 | 1.0 | 24 | 23.3 | 10.8 | 9.95 | 10.1 | 57.9 |
|  | 9/14/2016 | 1.3 | 22 | 22.8 | 13.4 | 12.1 | 10.7 | 51.4 |
|  | 9/15/2016 | 2.0 | 21 | 23 | 11.8 | 10.9 | 11.3 | 46.2 |
|  | 9/16/2016 | 3.0 | 24.4 | 25.9 | 12.6 | 12.1 | 13 | 46.7 |
|  | 9/16/2016 | 3.1 | 21.1 | 26.4 | 12.5 | 12.8 | 12.5 | 40.8 |
|  | 9/16/2016 | 3.2 | 19.3 | 22.4 | 14.4 | 14.3 | 14 | 27.5 |
|  | 9/16/2016 | 3.3 | 20.6 | 21.9 | 14.1 | 14.7 | 2 | 90.3 |
| Post-Contamination | 9/17/2016 | 4.0 | 20.4 | 22 | 14.1 | 13.7 | 13.3 | 34.8 |
|  | 9/18/2016 | 5.0 | 21.6 | 20.9 | 12.8 | 13 | 13.3 | 38.4 |
|  | 9/19/2016 | 6.0 | 20.4 | 20.7 | 12.8 | 12.6 | 13.3 | 34.8 |
|  | 9/20/2016 | 7.0 | 20.1 | 20.8 | 11.7 | 11.3 | 11.9 | 40.8 |
|  | 9/22/2016 | 9.0 | 21.2 | 21.6 | 13.2 | 12.8 | 13 | 38.7 |
|  | 9/27/2016 | 14.0 | 23.2 | 21.8 | 12.9 | 12.8 | 12.8 | 44.8 |
|  | 9/29/2016 | 16.0 | 22.4 | 23.8 | 15.3 | 15.2 | 15.1 | 32.6 |
|  | 10/4/2016 | 21.0 | 21.8 | 22.4 | 12.2 | 12.8 | 9.8 | 55.0 |
|  | 10/6/2016 | 23.0 | 21.9 | 23.5 | 13.7 | 13.9 | 14.4 | 34.2 |
|  | 10/11/2016 | 28.0 | 21.7 | 26.1 | 12.1 | 11.3 | 11.6 | 46.5 |
|  | 10/13/2016 | 30.0 | 19.4 | 22.2 | 13.1 | 13.1 | 13.3 | 31.4 |
|  | 10/17/2016 | 34.0 | 21.2 | 23.4 | 17.3 | 18.8 | 19.3 | 9.0 |
|  | 10/20/2016 | 37.0 | 19.1 | 22.4 | 15.6 | 16 | 17 | 11.0 |
|  | 10/25/2016 | 42.0 | 25.1 | 26.1 | 15.4 | 15.8 | 15.4 | 38.6 |
|  | 10/27/2016 | 44.0 | 22.2 | 22.9 | 16.5 | 15.6 | 16.1 | 27.5 |
|  | 11/1/2016 | 49.0 | 23.4 | 22.3 | 16.2 | 16.2 | 16.6 | 29.1 |
|  | 11/3/2016 | 51.0 | 23.7 | 23 | 14.3 | 14.2 | 14.8 | 37.6 |
|  | 11/8/2016 | 56.0 | 16.8 | 24.9 | 14.2 | 14.6 | 14.5 | 13.7 |
|  | 11/10/2016 | 58.0 | 22.8 | 24.6 | 17.5 | 18.4 | 18.1 | 20.6 |

| DO & Temperature in *B. globigii* experiment 1 | | | | | |
| --- | --- | --- | --- | --- | --- |
| Stage | Sampling Date | Sample Time (days) | DO (mg/L) Aeration Basin | Temperature (°C) Aeration Basin |  |
| Pre-contamination | 9/13/2016 | 0.0 | 1.94 | 22.6 |  |
| During Contamination | 9/13/2016 | 0.1 | 1.88 | 23.0 |  |
|  | 9/13/2016 | 0.2 | 1.98 | 23.2 |  |
|  | 9/13/2016 | 0.3 | 2.01 | 23.4 |  |
|  | 9/14/2016 | 1.0 | 2.06 | 23.4 |  |
|  | 9/14/2016 | 1.3 | 2.21 | 23.3 |  |
|  | 9/15/2016 | 2.0 | 2.84 | 23.5 |  |
|  | 9/16/2016 | 3.0 | 3.42 | 24.2 |  |
|  | 9/16/2016 | 3.1 | 3.81 | 24.4 |  |
|  | 9/16/2016 | 3.2 | 3.84 | 24.5 |  |
|  | 9/16/2016 | 3.3 | 3.93 | 24.5 |  |
| Post-Contamination | 9/17/2016 | 4.0 | 2.76 | 23.9 |  |
|  | 9/18/2016 | 5.0 | 2.73 | 24.1 |  |
|  | 9/19/2016 | 6.0 | 2.43 | 24.3 |  |
|  | 9/20/2016 | 7.0 | 2.51 | 23.8 |  |
|  | 9/22/2016 | 9.0 | 2.64 | 24.0 |  |
|  | 9/27/2016 | 14.0 | 2.79 | 24.0 |  |
|  | 9/29/2016 | 16.0 | 2.86 | 23.4 |  |
|  | 10/4/2016 | 21.0 | 2.91 | 22.0 |  |
|  | 10/6/2016 | 23.0 | 2.78 | 22.4 |  |
|  | 10/11/2016 | 28.0 | 2.75 | 22.2 |  |
|  | 10/13/2016 | 30.0 | 2.63 | 22.0 |  |
|  | 10/17/2016 | 34.0 | 2.70 | 21.9 |  |
|  | 10/20/2016 | 37.0 | 2.54 | 21.9 |  |
|  | 10/25/2016 | 42.0 | 2.43 | 21.4 |  |
|  | 10/27/2016 | 44.0 | 2.47 | 21.3 |  |
|  | 11/1/2016 | 49.0 | 2.21 | 21.0 |  |
|  | 11/3/2016 | 51.0 | 2.06 | 21.6 |  |
|  | 11/8/2016 | 56.0 | 2.11 | 21.4 |  |
|  | 11/10/2016 | 58.0 | 2.23 | 21.1 |  |

Experiment 2 data with B. globigii:

| *B. globigii* (CFU/mL) Experiment 2 | | | | | | | | | | | | | | | | | | | | | |
| --- | --- | --- | --- | --- | --- | --- | --- | --- | --- | --- | --- | --- | --- | --- | --- | --- | --- | --- | --- | --- | --- |
|  |  |  | SP-01 | | | | SP-03 | | | SP-04 | | | | SP-05 | | | | SP-06 | | |  |
| Stage | Sampling Date | Sample Time (Days) | Influent | | | | Primary Clarifier Effluent | | | Aeration Basin Effluent | | | | Return Activated Sludge | | | | Effluent | | |  |
| Pre-Contam-ination | 3/13/17 | 0 | 0.5 | 1.3 |  | 26 | |  | 25 | |  |  |  | 15 |  |  |  | 0.4 |  |  |  |
| During Contam-ination | 3/13/17 | 0.167 | 2.0E+05 | 2.6E+05 | 1.9E+05 | 1.0E+05 | | 4.6E+05 | 1.0E+05 | | 1.4E+05 | 1.1E+05 |  | 7.0E+04 | 5.9E+04 |  |  | 1.5E+04 | 1.8E+04 | 1.7E+04 |  |
| Post-Contam-ination | 3/20/17 | 7 | 150 | 220 | 170 | 1.3E+03 | | 1.4E+03 | 5.7E+05 | | 2.1E+05 | 1.9E+05 |  | 5.0E+04 | 1.0E+05 |  |  | 5.0E+03 | 5.0E+03 | 5.0E+03 |  |
|  | 3/27/17 | 14 | 3.5 |  |  | 5 | | 8.5 | 1.1E+04 | |  |  |  | 3.5E+03 | 4.8E+03 |  |  | 500 | 480 | 2.0E+03 |  |
|  | 4/4/17 | 21 | 5 | 5 |  | 9 | |  | 2.7E+04 | |  |  |  | 1.5E+03 | 2.4E+03 | 1.7E+03 |  | 250 | 160 |  |  |
|  | 4/11/17 | 28 | <5 |  |  | 5 | |  | 1.2E+04 | |  |  |  | 200 | 170 |  |  | 75 |  |  |  |
|  | 4/17/17 | 35 | 10 | 7 |  | 15 | | 13 | 950 | | 520 |  |  | 450 | 350 |  |  | 50 | 55 | 58 |  |
|  | 4/24/17 | 42 | 1 |  |  | 45 | | 26 | 1.7E+03 | | 1.4E+03 | 350 | 590 | 150 | 210 | 160 |  | 30 | 33 |  |  |
|  | 5/1/17 | 48 | 10 | 26 |  | 3.5 | |  | 500 | | 395 |  |  | 250 | 280 | 350 | 390 | 30 | 38.5 |  |  |

Note: The notation “<5” signifies the detection limit.

| TSS (mg/L) in *B. globigii* experiment 2 | | | | | | | | | | | | |
| --- | --- | --- | --- | --- | --- | --- | --- | --- | --- | --- | --- | --- |
|  |  |  | SP-01 | | SP-03 | | SP-04 | | SP-05 | | SP-06 | |
| Stage | Sampling Date | Sample Time (Days) | Influent | | Primary Clarifier Effluent | | Aeration Basin Effluent | | Return Activated Sludge | | Effluent | |
| Pre-contamination | 3/13/17 | 0 | 15 | 16 | 35 |  | 954 |  | 1043 |  | 7 |  |
| During Contamination | 3/13/17 | 0.167 | 20 |  | 20 | 22 | 1302 |  | 926 |  | 11 |  |
| Post-Contamination | 3/20/17 | 7 | 27 |  | 31 |  | 835 | 1307 | 742 |  | 6 |  |
|  | 3/27/17 | 14 | 37 |  | 31 |  | 363 |  | 459 | 460 | 6 |  |
|  | 4/4/17 | 21 | 9 |  | 5 |  | 544 |  | 314 |  | 1 | 2 |
|  | 4/11/17 | 28 | 10 | 9 | 116 |  | 489 |  | 244 |  | 28 |  |
|  | 4/17/17 | 35 | 10 |  | 17 | 17 | 282 |  | 289 |  | 22 |  |
|  | 4/24/17 | 42 | 2 |  | 26 |  | 305 | 271 | 212 |  | 34 |  |
|  | 5/1/17 | 48 | 14 |  | 26 |  | 321 |  | 320 | 360 | 31 |  |

| COD (mg/L) in *B. globigii* experiment 2 | | | | | | | | | | | | | |
| --- | --- | --- | --- | --- | --- | --- | --- | --- | --- | --- | --- | --- | --- |
|  |  |  | SP-01 | | SP-03 | | SP-04 | | SP-05 | | SP-06 | |  |
| Stage | Sampling Date | Sample Time (Days) | Influent | | Primary Clarifier Effluent | | Aeration Basin Effluent | | Return Activated Sludge | | Effluent | | COD Removal (%) |
| Pre-contamination | 3/13/2017 | 0 | 221 | 244 | 261 |  | 37 |  | 40 |  | 37 |  | 83.3 |
| During Contamination | 3/13/2017 | 0.167 | 242 |  | 242 | 231 | 96 |  | 80 |  | 64 |  | 73.6 |
| Post-Contamination | 3/20/2017 | 7 | 249 |  | 255 |  | 73 | 61 | 55 |  | 43 |  | 82.7 |
|  | 3/27/2017 | 14 | 206 |  | 229 |  | 95 |  | 66 | 70 | 68 |  | 67.0 |
|  | 4/4/2017 | 21 | 203 |  | 182 |  | 90 |  | 65 |  | 48 | 50 | 76.4 |
|  | 4/11/2017 | 28 | 222 | 159 | 221 |  | 68 |  | 54 |  | 29 |  | 86.9 |
|  | 4/17/2017 | 35 | 199 |  | 301 | 303 | 122 |  | 42 |  | 90 |  | 54.8 |
|  | 4/24/2017 | 42 | 223 |  | 247 |  | 78 | 81 | 68 |  | 62 |  | 72.2 |
|  | 5/1/2017 | 48 | 112 |  | 225 |  | 69 |  | 18 | 23 | 62 |  | 44.6 |

| NH3 (mg/L) in *B. globigii* experiment 2 | | | | | | | | | | | | | | |
| --- | --- | --- | --- | --- | --- | --- | --- | --- | --- | --- | --- | --- | --- | --- |
|  |  |  | SP-01 | | SP-03 | | SP-04 | | SP-05 | | | SP-06 | |  |
| Stage | Sampling Date | Sample Time (Days) | Influent | | Primary Clarifier Effluent | | Aeration Basin Effluent | | Return Activated Sludge | | | Effluent | | NH3 Removal (%) |
| Pre-contamination | 3/13/2017 | 0 | 24 | 24.2 | 27.5 |  | 16.2 |  | | 15.7 |  | 14.9 |  | 37.9 |
| During Contamination | 3/13/2017 | 0.167 | 22.6 |  | 23.7 | 20.5 | 16.6 |  | | 15.7 |  | 14.8 |  | 34.5 |
| Post-Contamination | 3/20/2017 | 7 | 24 |  | 27 |  | 16.8 | 16.7 | | 14.9 |  | 15.7 |  | 34.6 |
|  | 3/27/2017 | 14 | 23.1 |  | 24.6 |  | 17 |  | | 15.7 | 16.4 | 17.1 |  | 26.0 |
|  | 4/4/2017 | 21 | 22.6 |  | 21.3 |  | 20.7 |  | | 14.8 |  | 14.5 | 14.6 | 35.8 |
|  | 4/11/2017 | 28 | 23.9 | 23 | 24.1 |  | 18 |  | | 17.7 |  | 18.3 |  | 23.4 |
|  | 4/17/2017 | 35 | 21.3 |  | 31.5 | 31.2 | 14.6 |  | | 12.2 |  | 9.93 |  | 53.4 |
|  | 4/24/2017 | 42 | 22.6 |  | 23.1 |  | 19.3 | 17.2 | | 18.7 |  | 18.5 |  | 18.1 |
|  | 5/1/2017 | 48 | 12.2 |  | 24.9 |  | 22.6 |  | | 22.5 | 22.1 | 21 |  | 15.7 |

| DO & Temperature in *B. globigii* experiment 2 | | | | |
| --- | --- | --- | --- | --- |
| Stage | Sampling Date | Sample Time (Days) | DO (mg/L) Aeration Basin | Temperature (°C) Aeration Basin |
| Pre-contamination | 3/13/2017 | 0 | 6.6 | 18.6 |
| During Contamination | 3/13/2017 | 0.167 | 6.6 | 18.6 |
| Post-Contamination | 3/20/2017 | 7 | 5.63 | 18.3 |
|  | 3/27/2017 | 14 | 6.93 | 20.5 |
|  | 4/4/2017 | 21 | 4.9 | 20.7 |
|  | 4/11/2017 | 28 | 4.35 | 21.1 |
|  | 4/17/2017 | 35 | 5.69 | 22 |
|  | 4/24/2017 | 42 | 4.41 | 21.5 |
|  | 5/1/2017 | 48 | 4.49 | 23.3 |

Experiment 1 data with MS2:

| MS2 (PFU/100 mL) Experiment 1 | | | | | | | | | | | | |  |
| --- | --- | --- | --- | --- | --- | --- | --- | --- | --- | --- | --- | --- | --- |
|  |  |  | SP-01 | | SP-03 | | SP-04 | | SP-05 | | SP-06 | | |
| Stage | Sampling Date | Sample Time (days) | Influent | | Primary Clarifier Effluent | | Aeration Basin Effluent | | Return Activated Sludge | | Effluent | | |
| Pre-contamination | 9/25/17 | 0 | 1 |  | 1 |  | 1 |  | 1 |  | 1 |  | |
| During Contamination | 9/25/17 | 0.17 | 6.6E+06 | 1.1E+07 | 5.6E+06 | 6.0E+06 | 1.9E+06 | 1.0E+06 | 3.4E+06 | 1.0E+06 | 3E+06 | 3E+06 | |
|  | 9/26/17 | 1.00 | 6.5E+05 | 9.0E+05 | 8.5E+05 | 9.0E+05 | 5.0E+04 |  | 2.6E+05 |  | 4E+06 | 2E+05 | |
|  | 9/27/17 | 2.00 | 3.4E+04 | 2.0E+04 | 4.9E+04 | 1.0E+04 | 2.0E+03 |  | 3.0E+03 | 2.0E+04 | 1E+04 |  | |
|  | 9/28/17 | 3.00 | 380 | 600 | 3.8E+03 |  | 320 | 500 | 920 | 1.2E+03 | 870 | 900 | |
| Post-Contamination | 10/2/17 | 7.00 | 750 |  | 280 | 200 | 320 |  | 340 |  | 420 |  | |
|  | 10/9/17 | 14.00 | 1 |  | 21 | 40 | 1 |  | 4 |  | 2 | 10 | |
|  | 10/16/17 | 21.00 | 5 | 10 | 2 | 10 | 1 |  | 1 |  | 2 |  | |
|  | 10/23/17 | 28.00 | 1 |  | 5 |  | 1 |  | 1 |  | 1 |  | |
|  | 10/30/17 | 35.00 | 1 |  | 1 |  | 1 |  | 1 |  | 1 |  | |
|  | 11/6/17 | 42.00 | 1 |  | 1 |  | 1 |  | 1 |  | 1 |  | |
|  | 11/13/17 | 49.00 | 1 |  | 1 |  | 1 |  | 1 |  | 1 |  | |

Note: MS2 counts of “1” denote levels below the detection limits. Values of “1” were placed for graphing purposes.

| TSS (mg/L) in MS2 experiment 1 | | | | | | |
| --- | --- | --- | --- | --- | --- | --- |
|  |  | SP-1 | SP-3 | SP-4 | SP-5 | SP-6 |
| Sampling Date | Sample time (d) | Influent TSS | Primary Clarifier Effluent TSS | Aeration Basin Effluent TSS | Return Activated Sludge TSS | Effluent TSS |
| 9/26/2017 | 1 | 77 | 15 | 248 | 304 | 7 |
| 9/27/2017 | 2 | 1188 | 20 | 469 | 341 | 4 |
| 9/28/2017 | 3 | 52 | 21 | 642 | 400 | 93 |
| 10/2/2017 | 7 | 303 | 17 | 2736 | 707 | 60 |
| 10/9/2017 | 14 | 1712 | 26 | 1914 | 841 | 31 |
| 10/16/2017 | 21 | 653 | 26 | 1856 | 592 | 29 |
| 10/23/2017 | 28 | 105 | 18 | 4961 | 829 | 8 |
| 10/30/2017 | 35 | 2206 | 42 | 2044 | 747 | 29 |
| 11/6/2017 | 42 | 331 | 87 | 1375 | 538 | 18 |
| 11/13/2017 | 49 | 260 | 46 | 633 | 191 | 15 |

| COD (mg/L) in MS2 experiment 1 | | | | | | | | |
| --- | --- | --- | --- | --- | --- | --- | --- | --- |
|  |  | SP-01 | SP-03 | SP-04 | SP-05 | SP-06 |  |  |
| Sampling Date | Sample Time (days) | Influent COD | Primary Clarifier Effluent COD | Aeration Basin Effluent COD | Return Activated Sludge COD | Effluent COD | COD Removal (%) |  |
| 9/25/2017 | 0 | 238 | 212 |  | 80 | 76 | 68 |  |
| 9/25/2017 | 0.17 | 180 | 213 | 69 | 60 | 112 | 38 |  |
| 9/26/2017 | 1.00 | 150 | 196 | 52 | 54 | 48 | 68 |  |
| 9/27/2017 | 2.00 | 202 | 217 | 90 | 95 | 80 | 60 |  |
| 9/28/2017 | 3.00 | 229 | 219 | 73 | 81 | 66 | 71 |  |
| 10/2/2017 | 7.00 | 290 | 213 | 79 | 81 | 70 | 76 |  |
| 10/9/2017 | 14.00 | 213 | 212 | 76 | 77 | 70 | 67 |  |
| 10/16/2017 | 21.00 | 244 | 207 | 121 | 77 | 76 | 69 |  |
| 10/23/2017 | 28.00 | 218 | 209 | 75 | 60 | 64 | 71 |  |
| 10/30/2017 | 35.00 | 299 | 250 | 66 | 67 | 73 | 76 |  |
| 11/6/2017 | 42.00 | 144 | 209 | 57 | 70 | 53 | 63 |  |
| 11/13/2017 | 49.00 | 159 | 203 | 71 | 72 | 70 | 56 |  |

| NH3 (mg/L) in MS2 experiment 1 | | | | | | | |
| --- | --- | --- | --- | --- | --- | --- | --- |
|  |  | SP-01 | SP-03 | SP-04 | SP-05 | SP-06 |  |
| Sampling Date | Sample Time (d) | Influent NH3 | Primary Clarifier Effluent NH3 | Aeration Basin Effluent NH3 | Return Activated Sludge NH3 | Effluent NH3 | NH3 Removal (%) |
| 9/25/2017 | 0 | 23.7 | 24.4 | 12.2 | 7.01 | 6.83 | 71 |
| 9/25/2017 | 0.17 | 18.2 | 23.8 | 14.1 | 14.3 | 13.8 | 24 |
| 9/26/2017 | 1 | 22.5 | 22.3 | 9.14 | 8.79 | 10.8 | 52 |
| 9/27/2017 | 2 | 20.1 | 22.4 | 5.93 | 5.75 | 6.85 | 66 |
| 9/28/2017 | 3 | 22.2 | 22.3 | 3.28 | 3.25 | 3.56 | 84 |
| 10/2/2017 | 7 | 27.2 | 23.8 | 2.65 | 3.05 | 2.81 | 90 |
| 10/9/2017 | 14 | 27.8 | 29 | 11.7 | 11.7 | 12.3 | 56 |
| 10/16/2017 | 21 | 25.9 | 24.9 | 7.19 | 7.33 | 7.01 | 73 |
| 10/23/2017 | 28 | 22.1 | 23.7 | 7.63 | 5.99 | 6.27 | 72 |
| 10/30/2017 | 35 | 25.6 | 23.3 | 5.74 | 5.92 | 6.04 | 76 |
| 11/6/2017 | 42 | 5.79 | 21.9 | 16.8 | 16.5 | 16.8 | 23 |
| 11/13/2017 | 49 | 14.1 | 21.8 | 19.8 | 19.3 | 20.5 | 6 |

| DO & Temperature in MS2 experiment 1 | | | | |
| --- | --- | --- | --- | --- |
| Sampling Date | Sample Time (d) | DO (mg/L) Aeration Basin | Temperature (°C) Aeration Basin |  |
| 9/25/2017 | 0.17 | 5.4 | 27.1 |  |
| 9/26/2017 | 1 | 4.6 | 27.7 |  |
| 9/27/2017 | 2 | 5.6 | 26.2 |  |
| 9/28/2017 | 3 | 4.9 | 26.8 |  |
| 10/2/2017 | 7 | 4.6 | 26.5 |  |
| 10/9/2017 | 14 | 4.2 | 27.0 |  |
| 10/16/2017 | 21 | 4.5 | 24.6 |  |
| 10/23/2017 | 28 | 4.9 | 25.4 |  |
| 10/30/2017 | 35 | 4.9 | 21.7 |  |
| 11/6/2017 | 42 | 5.0 | 23.2 |  |
| 11/13/2017 | 49 | 5.7 | 20.7 |  |

Experiment 2 data with MS2:

| MS2 (PFU/100 mL) experiment 2 | | | | | | | | |
| --- | --- | --- | --- | --- | --- | --- | --- | --- |
|  |  |  | SP-01 | SP-03 | SP-04 | SP-05 | SP-06 |  |
| Stage | Sampling Date | Sample Time (days) | Influent | Primary Clarifier Effluent | Aeration Basin Effluent | Return Activated Sludge | Effluent | Injection Suspension |
| Pre-contamination | 3/19/2018 | 0 | 1 | 1 | 1 | 1 | 1 | -- |
| During Contamination | 3/19/2018 | 0.17 | 1.0E+06 | 1.1E+06 | 7.4E+05 | 5.2E+05 | 3.2E+05 | 7.2E+08 |
|  | 3/20/2018 | 1.00 | 5.3E+05 | 4.6E+05 | 4.9E+05 | 4.6E+05 | 4.2E+05 | 3.5E+08 |
|  | 3/21/2018 | 2.00 | 5.4E+05 | 7.4E+05 | 5.5E+05 | 5.1E+05 | 1.9E+06 | 5.2E+09 |
|  | 3/22/2018 | 3.00 | 1.1E+06 | 1.1E+06 | 1.1E+06 | 1.3E+06 | 1.4E+06 | 4.5E+08 |
| Post-Contamination | 3/26/2018 | 7.00 | 7.5E+02 | 6.5E+02 | 2.2E+03 | 1.4E+03 | 1.3E+03 | -- |
|  | 4/2/2018 | 14.00 | 1.3E+01 | 2.7E+01 | 7.0E+01 | 2.6E+01 | 9.1E+01 | -- |
|  | 4/9/2018 | 21.00 | 9.0 | 3.5E+01 | 4.1E+01 | 3.5E+01 | 2.6E+01 | -- |
|  | 4/16/2018 | 28.00 | 6.7E+01 | 4.0E+00 | 4.0 | 1.7E+01 | 3 | -- |
|  | 4/23/2018 | 35.00 | 4.0 | 1 | 1 | 2 | 1 | -- |
|  | 4/30/2018 | 42.00 | 6.0 | 1 | 3.0 | 1 | 1 | -- |
|  | 5/7/2018 | 49.00 | 1 | 1 | 1 | 1 | 1 | -- |

Note: MS2 counts of “1” denote levels below the detection limits. Values of “1” were placed for graphing purposes.

| *TSS* (mg/L) in MS2 experiment 2 | | | | | | | |
| --- | --- | --- | --- | --- | --- | --- | --- |
|  |  | SP-01 | SP-03 | SP-04 | SP-05 | SP-06 |  |
| Sampling Date | sample time (days) | Influent | Primary Clarifier Effluent | Aeration Basin Effluent | Return Activated Sludge | Effluent |  |
| 3/13/2018 | 0 | 9 | 9 | 946 | 647 | 5 |  |
| 3/19/2018 | 0.1666667 | 25 | 14 | 1246 | 802 | 4 |  |
| 3/20/2018 | 1 | 25 | 23 | 822 | 789 | 8 |  |
| 3/21/2018 | 2 | 12 | 35 | 818 | 637 | 2 |  |
| 3/22/2018 | 3 | 25 | 21 | 840 | 517 | 7 |  |
| 3/26/2018 | 7 | 13 | 26 | 757 | 460 | 6 |  |
| 4/2/2018 | 14 | 39 | 21 | 331 | 290 | 64 |  |
| 4/9/2018 | 21 | 32 | 18 | 340 | 158 | 13 |  |
| 4/16/2018 | 28 | 4836 | 53 | 467 | 311 | 82 |  |
| 4/23/2018 | 35 | 62 | 20 | 397 | 238 | 29 |  |
| 4/30/2018 | 42 | 512 | 30 | 525 | 330 | 134 |  |
| 5/7/2018 | 49 | 70 | 25 | 482 | 222 | 6 |  |

| COD (mg/L) in MS2 experiment 2 | | | | | | | | |
| --- | --- | --- | --- | --- | --- | --- | --- | --- |
|  |  | SP-01 | SP-03 | SP-04 | SP-05 | SP-06 |  |  |
| Sampling Date | Sample Time (days) | Influent | Primary Clarifier Effluent | Aeration Basin Effluent | Return Activated Sludge | Effluent | COD Removal (%) |  |
| 3/19/2018 | 0 | 223 | 202 | 70 | 56 | 46 | 79 |  |
| 3/19/2018 | 0.17 | 147 | 189 | 68 | 43 | 53 | 64 |  |
| 3/20/2018 | 1.00 | 163 | 194 |  | 40 | 36 | 78 |  |
| 3/21/2018 | 2.00 | 183 | 180 | 50 | 36 | 35 | 81 |  |
| 3/22/2018 | 3.00 | 185 | 180 | 44 | 48 | 40 | 78 |  |
| 3/26/2018 | 7.00 | 179 | 218 | 78 | 41 | 40 | 78 |  |
| 4/2/2018 | 14.00 | 178 | 194 | 124 | 117 | 104 | 42 |  |
| 4/9/2018 | 21.00 | 230 | 226 | 147 | 121 | 140 | 39 |  |
| 4/16/2018 | 28.00 | 475 | 229 | 115 | 108 | 102 | 79 |  |
| 4/23/2018 | 35.00 | 263 | 223 | 146 | 96 | 98 | 63 |  |
| 4/30/2018 | 42.00 | 336 | 333 | 64 | 74 | 71 | 79 |  |
| 5/7/2018 | 49.00 | 195 | 238 | 108 | 86 | 94 | 52 |  |

| NH3 (mg/L) in MS2 experiment 2 | | | | | | | | |
| --- | --- | --- | --- | --- | --- | --- | --- | --- |
|  |  | SP-01 | SP-03 | SP-04 | SP-05 | SP-06 |  |  |
| Sampling Date | Sample Time (days) | Influent | Primary Clarifier Effluent | Aeration Basin Effluent | Return Activated Sludge | Effluent | NH3 Removal (%) |  |
| 3/19/2018 | 0 | 21.2 | 20.2 | 12 | 11.6 | 11.6 | 45 |  |
| 3/19/2018 | 0.17 | 13.5 | 19.1 | 10.4 | 8.77 | 8.87 | 54 |  |
| 3/20/2018 | 1.00 | 16.8 | 19.2 | 9.94 | 9.08 | 9.38 | 51 |  |
| 3/21/2018 | 2.00 | 21.4 | 19.6 | 10.3 | 8.8 | 8.51 | 60 |  |
| 3/22/2018 | 3.00 | 19.5 | 19.2 | 10.3 | 9.16 | 9.11 | 53 |  |
| 3/26/2018 | 7.00 | 18.6 | 24.1 | 11.4 | 9.54 | 8.19 | 56 |  |
| 4/2/2018 | 14.00 | 12.7 | 17 | 13.2 | 12.7 | 12.9 | 24 |  |
| 4/9/2018 | 21.00 | 19.6 | 19.9 | 16.4 | 15.6 | 15.4 | 21 |  |
| 4/16/2018 | 28.00 | 21.4 | 19.2 | 12.7 | 12.6 | 12.6 | 41 |  |
| 4/23/2018 | 35.00 | 23.2 | 18.8 | 10.7 | 10 | 9.84 | 58 |  |
| 4/30/2018 | 42.00 | 27.5 | 19.1 | 10.2 | 9.16 | 9.65 | 65 |  |
| 5/7/2018 | 49.00 | 14.1 | 18.9 | 16.2 | 14.8 | 15.1 | 20 |  |

Note, in NH_3_ removal column, yellow cells calculated NH_3_ removal using SP3 as the starting level, while the plain cells calculated removal using SP1 data as the starting level.

| DO & Temperature in MS2 experiment 2 | | | |
| --- | --- | --- | --- |
| Analysis Date | Sample Time (days) | DO (mg/L) | Temp C |
| 3/19/2018 | 0.17 | 3.6 | 20.9 |
| 3/20/2018 | 1.00 | 3.7 | 19.6 |
| 3/21/2018 | 2.00 | 4.2 | 20.3 |
| 3/22/2018 | 3.00 | 5.7 | 19.6 |
| 3/26/2018 | 7.00 | 8.0 | 19.9 |
| 4/2/2018 | 14.00 | 5.4 | 19.9 |
| 4/9/2018 | 21.00 | 5.9 | 19.5 |
| 4/16/2018 | 28.00 | 5.9 | 20.8 |
| 4/23/2018 | 35.00 | 5.1 | 23.4 |
| 4/30/2018 | 42.00 | 5.7 | 23.7 |
| 5/7/2018 | 49.00 | 5.0 | 22.9 |

Experiment 1 data with Phi-6:

| Phi-6 (PFU/100mL) Experiment 1 | | | | | | | | |
| --- | --- | --- | --- | --- | --- | --- | --- | --- |
|  |  |  | SP-01 | SP-03 | SP-04 | SP-05 | SP-06 |  |
| Stage | Sampling Date | Sampling Time (d) | Influent | Primary Clarifier Effluent | Aeration Basin Effluent | Return Activated Sludge | Effluent |  |
| Pre-contamination | 12/10/2018 | 0 | 1 | 1 | 1 | 1 | 1 |  |
| During Contamination | 12/10/2018 | 0.17 | 1.0E+00 | 3.0E+02 | 1 | 1 | 1 |  |
|  | 12/10/2018 | 0.33 | 2.6E+06 | 4.2E+04 | 1 | 1.6E+03 | 3.0E+02 |  |
|  | 12/11/2010 | 1 | 2.3E+06 | 4.1E+03 | 1 | 1 | 3.0E+02 |  |
| Post-Contamination | 12/12/2010 | 2 | 1 | 3.0E+02 | 1.0E+02 | 1.0E+02 | 1 |  |
|  | 12/13/2010 | 3 | 7.5E+02 | 2.4E+02 | 3.2E+02 | 3.4E+02 | 4.2E+02 |  |
|  | 12/14/2010 | 4 | 1 | 1 | 1 | 1 | 1 |  |
|  | 12/17/2018 | 7 | 1 | 1 | 1 | 1 | 1 |  |

Note: Phi-6 counts of “1” denote levels below the detection limits. Values of “1” were placed for graphing purposes.

| TSS (mg/L) in Phi-6 experiment 1 | | | | | | | |
| --- | --- | --- | --- | --- | --- | --- | --- |
|  |  | SP1 | SP3 | SP4 | SP5 | SP6 |  |
| Sampling Date | Sampling Time (d) | Influent | Primary Clarifier Effluent | Aeration Basin Effluent | Return Activated Sludge | Effluent |  |
| 12/10/2018 | 0 | 60.29 | 22.22 | 1250.00 | 873.17 | 2.84 |  |
| 12/10/2018 | 0.17 | 101.48 | 19.72 | 1107.32 | 823.81 | 0.75 |  |
| 12/10/2018 | 0.33 | 130.40 | 17.65 | 4021.05 | 711.90 | 1.48 |  |
| 12/11/2010 | 1 | 62.32 | 22.54 | 1017.07 | 840.38 | 3.52 |  |
| 12/12/2010 | 2 | 109.92 | 27.08 | 955.10 | 847.92 | 14.69 |  |
| 12/13/2010 | 3 | 103.52 | 31.47 | 1020.75 | 859.18 | 2.11 |  |
| 12/14/2010 | 4 | 97.20 | 33.09 | 1135.29 | 834.62 | 2.86 |  |
| 12/17/2018 | 7 | 213.87 | 57.64 | 1090.20 | 852.00 | 4.83 |  |

| COD (mg/L) in Phi-6 experiment 1 | | | | | | | | |
| --- | --- | --- | --- | --- | --- | --- | --- | --- |
|  |  | SP-01 | SP-03 | SP-04 | SP-05 | SP-06 |  |  |
| Sampling Date | Sampling Time (d) | Influent | Primary Clarifier Effluent | Aeration Basin Effluent | Return Activated Sludge | Effluent | % Removal (B/w SP3 & SP6) |  |
| 12/10/2018 | 0 | 219 | 179 | 26 | 23 | 19 | 89 |  |
| 12/10/2018 | 0.17 | 388 | 302 | 47 | 43 | 37 | 88 |  |
| 12/10/2018 | 0.33 | 414 | 336 | 66 | 56 | 42 | 88 |  |
| 12/11/2010 | 1 | 379 | 260 | 67 | 67 | 56 | 78 |  |
| 12/12/2010 | 2 | 279 | 180 | 45 | 43 | 49 | 73 |  |
| 12/13/2010 | 3 | 320 | 209 | 39 | 42 | 46 | 78 |  |
| 12/14/2010 | 4 | 328 | 214 | 62 | 45 | 38 | 82 |  |
| 12/17/2018 | 7 | 332 | 250 | 39 | 50 | 45 | 82 |  |

| NH3 (mg/L) in Phi-6 experiment 1 | | | | | | | |
| --- | --- | --- | --- | --- | --- | --- | --- |
|  |  | SP-01 | SP-03 | SP-04 | SP-05 | SP-06 | % Removal |
| Sampling Date | Sampling Time (d) | Influent | Primary Clarifier Effluent | Aeration Basin Effluent | Return Activated Sludge | Effluent | B/w SP3 & SP6 |
| 12/10/2018 | 0 | 22.1 | 24.6 | 14.1 | 14.6 | 14.1 | 43 |
| 12/10/2018 | 0.17 | 22.9 | 22.8 | 15 | 15.4 | 14.7 | 36 |
| 12/10/2018 | 0.33 | 23.5 | 22.6 | 17.8 | 16 | 15.2 | 33 |
| 12/11/2010 | 1 | 21.8 | 23.2 | 17.1 | 16.9 | 17 | 27 |
| 12/12/2010 | 2 | 22.9 | 23.6 | 15.1 | 15.1 | 15.1 | 36 |
| 12/13/2010 | 3 | 21.3 | 22.3 | 13.5 | 13.6 | 14.1 | 37 |
| 12/14/2010 | 4 | 26.4 | 22.8 | 13.5 | 13.6 | 13.8 | 39 |
| 12/17/2018 | 7 | 22.9 | 23.6 | 13 | 13 | 13.1 | 44 |

| DO and Temperature in Phi-6 experiment 1 | | | |
| --- | --- | --- | --- |
| Sampling Date | Time in hours | DO (mg/L) | Temp (°C) |
| 12/10/2018 | 0.17 | 5.0 | 28.5 |
| 12/11/2010 | 1 | 5.1 | 26.9 |
| 12/12/2010 | 2 | 4.9 | 27.3 |
| 12/13/2010 | 3 | 5.1 | 27.8 |
| 12/14/2010 | 4 | 5.0 | 28.1 |
| 12/17/2018 | 7 | 4.8 | 27.7 |

Experiment 2 data with Phi-6:

| Phi-6 (PFU/100mL) Experiment 2 | | | | | | | |
| --- | --- | --- | --- | --- | --- | --- | --- |
|  |  |  | SP-01 | SP-03 | SP-04 | SP-05 | SP-06 |
| Stage | Sampling Date | Sampling Time (d) | Influent | Primary Clarifier Effluent | Aeration Basin Effluent | Return Activated Sludge | Effluent |
| Pre-contamination | 5/6/2019 | 0 | 1 | 1 | 1 | 1 | 1 |
| During Contamination | 5/6/2019 | 0.17 | 1 | 8.9E+03 | 1.0E+03 | 1.0E+03 | 1.0E+03 |
|  | 5/6/2019 | 0.33 | 6.6E+06 | 8.7E+04 | 5.3E+04 | 7.0E+04 | 1.0E+05 |
|  | 5/7/2019 | 1 | 3.0E+04 | 1 | 1 | 1 | 7.0E+03 |
| Post-Contamination | 5/8/2019 | 2 | 1 | 1 | 1 | 1 | 1 |
|  | 5/9/2019 | 3 | 1 | 1 | 1 | 1 | 1 |
|  | 5/10/2019 | 4 | 1 | 1 | 1 | 1 | 1 |
|  | 5/13/2019 | 7 | 1 | 1 | 1 | 1 | 1 |

Note: Phi-6 counts of “1” denote levels below the detection limits. Values of “1” were placed for graphing purposes.

| TSS (mg/L) in Phi-6 experiment 2 | | | | | | |
| --- | --- | --- | --- | --- | --- | --- |
|  |  | SP1 | SP3 | SP4 | SP5 | SP6 |
| Sampling Date | Sampling Time (d) | Influent | Primary Clarifier Effluent | Aeration Basin Effluent | Return Activated Sludge | Effluent |
| 5/6/2019 | 0 | 71 | 21 | 585 | 532 | 18 |
| 5/6/2019 | 0.17 | 138 | 41 | 893 | 667 | 13 |
| 5/6/2019 | 0.33 | 45 | 32 | 746 | 728 | 12 |
| 5/7/2019 | 1 | 60 | 34 | 2369 | 740 | 23 |
| 5/8/2019 | 2 | 372 | 40 | 7984 | 801 | 18 |
| 5/9/2019 | 3 | 94 | 40 | 1183 | 743 | 25 |
| 5/10/2019 | 4 | 306 | 52 | 989 | 845 | 12 |
| 5/13/2019 | 7 | 77 | 12 | 4650 | 626 | 15 |

| COD (mg/L) in Phi-6 experiment 2 | | | | | | | |
| --- | --- | --- | --- | --- | --- | --- | --- |
|  |  | SP-01 | SP-03 | SP-04 | SP-05 | SP-06 | % Removal |
| Sampling Date | Sampling Time (d) | Influent | Primary Clarifier Effluent | Aeration Basin Effluent | Return Activated Sludge | Effluent | B/w SP3 & SP6 |
| 5/2/2019 | 0 | 285 | 190 | 95 | 43 | 49 | 74 |
| 5/6/2019 | 0.17 | 346 | 453 | 85 | 58 | 45 | 90 |
| 5/6/2019 | 0.33 | 385 | 370 | 71 | 80 | 65 | 82 |
| 5/7/2019 | 1 | 190 | 341 | 146 | 124 | 98 | 71 |
| 5/8/2019 | 2 | 234 | 172 | 137 | 40 | 36 | 79 |
| 5/9/2019 | 3 | 245 | 177 | 38 | 50 | 44 | 75 |
| 5/10/2019 | 4 | 269 | 183 | 30 | 29 | 45 | 75 |
| 5/13/2019 | 7 | 181 | 158 | 121 | 42 | 23 | 85 |

| NH3 (mg/L) in Phi-6 experiment 2 | | | | | | | |
| --- | --- | --- | --- | --- | --- | --- | --- |
|  |  | SP-01 | SP-03 | SP-04 | SP-05 | SP-06 | % Removal |
| Sampling Date | Sampling Time (d) | Influent | Primary Clarifier Effluent | Aeration Basin Effluent | Return Activated Sludge | Effluent | B/w SP3 & SP6 |
| 5/6/2019 | 0 | 21.2 | 22.6 | 9.32 | 9.45 | 9.17 | 59 |
| 5/6/2019 | 0.17 | 35.4 | 34.3 | 10.2 | 10.9 | 9.58 | 72 |
| 5/6/2019 | 0.33 | 20.1 | 25.1 | 13.9 | 14 | 12.2 | 51 |
| 5/7/2019 | 1 | 16.7 | 25.3 | 12.5 | 11.8 | 12 | 53 |
| 5/8/2019 | 2 | 19.6 | 21.6 | 10 | 9.19 | 9.2 | 57 |
| 5/9/2019 | 3 | 21 | 22.8 | 9.62 | 9.17 | 9.21 | 60 |
| 5/10/2019 | 4 | 21 | 22.3 | 8.92 | 9.06 | 8.91 | 60 |
| 5/13/2019 | 7 | 19.5 | 20.7 | 8.6 | 7.77 | 7.54 | 64 |

| DO & Temperature in Phi-6 experiment 2 | | | |
| --- | --- | --- | --- |
| Sampling Date | Time in hours | DO (mg/L) | Temp (°C) |
| 5/6/2019 | 0.17 | 5.4 | 26.0 |
| 5/7/2019 | 1 | 5.1 | 26.3 |
| 5/8/2019 | 2 | 5.9 | 25.8 |
| 5/9/2019 | 3 | 5.9 | 25.5 |
| 5/10/2019 | 4 | 6.5 | 24.4 |
| 5/13/2019 | 7 | 6.1 | 24.1 |
